# Examining the Association Between Internet Use and Perceived Stress in Adults: Longitudinal Observational Study Combining Web Tracking Data With Questionnaires

**DOI:** 10.2196/78775

**Published:** 2026-01-09

**Authors:** Mohammad Belal, Nguyen Luong, Talayeh Aledavood, Juhi Kulshrestha

**Affiliations:** 1 Department of Computer Science, Aalto University, PL 1400, Konemiehentie 2, Aalto, 00076, Finland, 358 504135540

**Keywords:** online behavior, stress, internet use, web browsing traces, sociodemographic differences, longitudinal design

## Abstract

**Background:**

In today’s digital era, the internet plays a pervasive role in daily life, influencing everyday activities such as communication, work, and leisure. This online engagement intertwines with offline experiences, shaping individuals’ overall well-being. Despite its significance, existing research often falls short in capturing the relationship between internet use and well-being, relying primarily on isolated studies and self-reported data. One major contributor to deteriorated well-being is stress. While some research has examined the relationship between internet use and stress, both positive and negative associations have been reported.

**Objective:**

This study aimed to identify the associations between an individual’s internet use and their stress.

**Methods:**

We conducted a 7-month longitudinal study. We combined fine-grained URL-level web browsing traces of 1490 German internet users with their sociodemographics and monthly measures of stress. Further, we developed a conceptual framework that allows us to simultaneously explore different contextual dimensions, including how, where, when, and by whom the internet is used. We applied linear mixed-effects models to examine these associations.

**Results:**

Our analysis revealed several associations between internet use and stress, varying by context. Increased time spent on social media, online shopping, and gaming platforms was associated with higher stress. For example, the time spent by individuals on shopping-related internet use (aggregated over the 30 days before their stress was measured via questionnaires) was positively associated with stress on both mobile (*β*=.04, 95% CI 0.00‐0.08; *P*=.04) and desktop devices (*β*=.03, 95% CI −0.00 to 0.06; *P*=.09). In contrast, time spent on productivity or news websites was associated with lower stress. Specifically, in the last 30 days of mobile usage, productivity-related use showed a negative association with stress (*β*=−.03, 95% CI −0.06 to −0.00; *P*=.04). In addition, in the last 2 days of data, news usage was negatively associated with stress on both mobile (*β*=−.54, 95% CI −1.08 to 0.00; *P*=.048) and desktop devices (*β*=−.50, 95% CI−0.90 to −0.11; *P*=.01). Further analysis showed that total time spent online (*β*=.01, 95% CI 0.00‐0.02; *P*<.001), social-media usage (*β*=.02, 95% CI 0.00‐0.03; *P*=.02), and gaming usage (*β*=.01, 95% CI 0.00‐0.02; *P*=.02) were all positively associated with stress in high-stress Perceived Stress Scale (PSS>26) individuals on mobile devices.

**Conclusions:**

The findings indicate that internet use is associated with stress, and these associations differ across various usage contexts. In the future, the behavioral markers we identified can pave the way for designing individualized tools for people to self-monitor and self-moderate their online behaviors to enhance their well-being, reducing the burden on already overburdened mental health services.

## Introduction

Stress is an unavoidable part of human life, arising from the demands and challenges we face daily. It is a significant factor in health issues such as cardiovascular disease, weakened immune function, and mental health challenges [1,2]. The internet, now an integral part of modern life, has sparked debates about its impact on stress levels and psychological well-being [3,4], as well as whether this influence is predominantly positive or negative. As our online and offline lives become increasingly interconnected, understanding the relationship between internet use and stress has gained considerable attention.

While the internet offers numerous advantages, such as enhanced connectivity and easy access to information, excessive or problematic use has been linked to various stress-related factors [3,5,6]. For example, heavy internet use has been associated with higher levels of anxiety [5], while the amount of time spent online has been linked to sleep loss and withdrawal [6]. On the other hand, past research suggests that not all forms of internet use are detrimental; certain online activities have been associated with reduced stress and improved psychological well-being [7-9].

Despite the internet’s widespread influence, research on psychological well-being, including stress, has primarily focused on offline activities, leaving a critical gap in understanding how online behaviors impact stress and well-being [10]. For instance, a comprehensive review of 99 commonly used psychological well-being scales identified 196 dimensions, yet none explicitly addressed online activities or behaviors [10]. Moreover, studies on online engagement have often faced limitations, including short study durations, small sample sizes, and an over-reliance on questionnaires to capture internet use patterns. These approaches can introduce biases and fail to provide a complete picture of the connection between online and offline experiences [11,12].

In this paper, we first review previous research on the association between internet use and stress and examine the methodologies used in these studies. We then outline our longitudinal multimodal study design, which integrates actual internet usage data with monthly questionnaires to measure stress, and discuss our study’s potential impact.

### Conflicting Findings on Associations Between Stress and Internet Use

The relationship between internet use and stress is complex, with previous research showing contrasting associations depending on the type and context of digital engagement, as well as individual characteristics. High levels of internet and smartphone use have been linked to increased stress [13,14], often due to digital overload (ie, the cognitive strain caused by constant notifications and an endless stream of information) [15,16]. In contrast, internet use through computers has been associated with less burnout compared to smartphone use [17]. However, these associations are not consistent. For instance, age influences the impact of digital multitasking: younger users report higher stress than older adults when handling multiple digital tasks, yet they appear less affected by communication overload [15]. Experimental evidence shows that multitasking increases perceived stress levels in both younger and older adults, with no significant differences between the age groups [18]. Other studies have shown no association [19] or even a negative association between time spent online and stress, particularly in young adults [20].

Moreover, the type of online activity plays a crucial role in stress outcomes. Social networking and entertainment-related use have been associated with higher stress levels, while internet use for work-related tasks has been linked to greater life satisfaction in the middle-aged population [21]. Research also indicates that communication overload from emails and messages is positively associated with perceived stress in the age group of 50‐85 years [15]. Studies on social media show similarly nuanced findings. While Pew Research found no association between social media use and stress in men, a negative association was observed in women [22]. A large-scale study also showed slightly higher perceived stress among high social media users than nonusers [23]. Other digital behaviors, such as problematic news consumption [24] and adult content addiction [25], have also been linked to heightened stress and emotional distress. Similarly, concerns such as cyberbullying, online harassment [26], work-life boundary erosion [27], and data privacy issues [28] have been widely documented as stress-inducing. Further studies have found positive associations between stress and various digital behaviors, such as online shopping addiction in young people [29], negative information seeking [30], interpersonal communication in older adults [7], misinformation sharing [31], and excessive gaming in adolescents [32,33].

Conversely, the internet can also act as a buffer against stress [20], offering access to supportive communities [9], relaxation tools, and leisure activities [7]. Online entertainment and social interaction, in particular, have been shown to reduce stress and enhance well-being among older adults [7,8,34]. In addition, internet use has been recognized as a coping mechanism. Several online activities, including social media [35], entertainment [36,37], shopping [38], and gaming [35,39], have been identified in previous studies as strategies for managing stress.

### User Characteristics Shape the Relationship Between Online Activity and Stress

Studies show that age, gender, income, and baseline stress levels influence how online activities relate to stress [15,18,22]. Social media use has been negatively associated with stress among females [22], though females overall report higher stress than males [40,41]. Older adults tend to report lower stress than younger groups but experience stronger associations between communication load, frequent messaging, and stress [42,43]. Higher income is generally linked to lower stress levels [44], though findings differ by context—for instance, higher income was associated with fewer mental health issues in Germany but with more issues in China [45]. Social media use has also been linked to slower recovery from real-world stressors, suggesting possible stress maintenance in already stressed individuals [46].

### Methods for Identifying Connections Between Internet Use and Stress

Past research on internet use and stress has used various methodologies. Many studies rely on cross-sectional designs and self-reported surveys [7,15,16,21,24,32]. These studies often focus on specific populations, such as university or medical students [13,14]. Some have used larger samples [23,24,32] but still rely on questionnaires to capture internet use. A smaller number of experimental studies are available [30,46], and some have adopted alternative approaches, such as analyzing social media data to infer psychological states [31]. Studies using actual web browsing data [20,47] are limited and tend to capture general metrics, such as total time spent online [20], and are based on relatively small samples (92 and 107 participants), indicating how difficult it is to conduct such studies.

### Contributions and Impact of Our Study

Previous research on the relationship between internet use and stress has shown mixed findings, revealing both negative and positive associations depending on the type of internet activity. However, much of this evidence remains fragmented, as previous studies have largely relied on self-reported internet use data (which often lacks granularity) and have focused on limited aspects of internet use.

**Figure 1.**
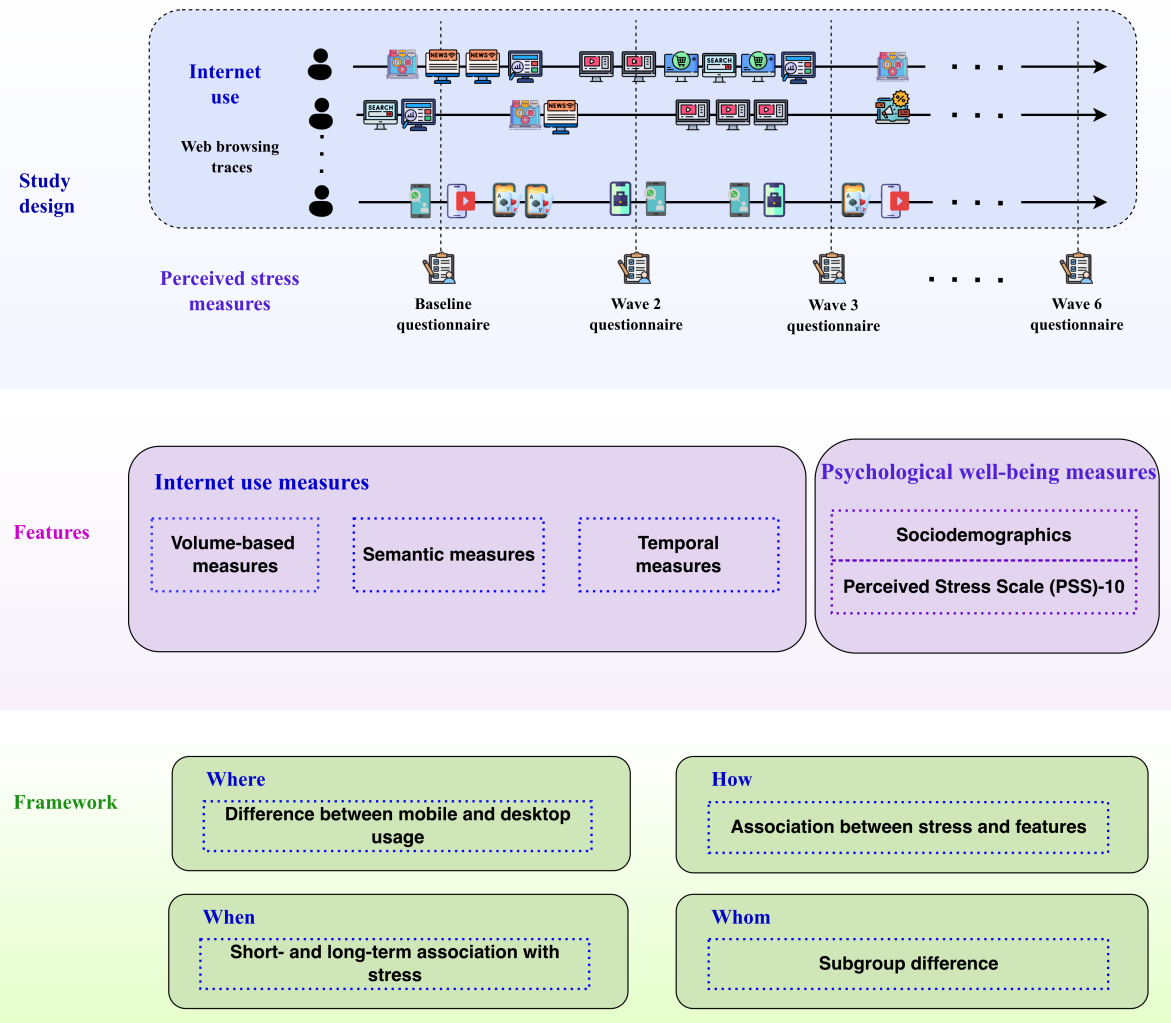
Overview of the study design and contextual dimensions. The top panel shows our longitudinal study design combining desktop and mobile web‐trace data with monthly stress questionnaires. The middle panel depicts the internet use and well-being features extracted from web browsing traces and monthly questionnaires. The bottom panel shows the contextual dimensions we consider for examining associations between internet use and stress.

To address these limitations and to provide a more comprehensive understanding of how internet use relates to stress, we conducted a longitudinal multimodal study involving 1490 internet users in Germany over 7 months. Our study integrates fine-grained, passively collected web trace data from both desktop and mobile devices with participants’ monthly responses to a validated stress scale (refer to Figure 1). Using objective behavioral data, we move beyond subjective self-reports and introduce a data-driven framework for revealing long-term usage patterns and identifying digital markers of stress.

Building on existing research, we further identify 4 key dimensions that shape the relationship between digital behavior and stress:

1. How: the type and pattern of internet behaviors—such as usage volume [14,48], temporal rhythms [49], and content categories (eg, social media, productivity, entertainment, or shopping)—may influence stress in different ways [21,50-52].
2. Where: the device context plays a significant role. Previous research suggests that desktop use is often more goal-oriented and structured, whereas mobile use tends to be more fragmented and reactive [53].
3. When: the timing of online behaviors in relation to stress assessments is important, as short-term engagement with digital content may have immediate effects on stress responses [54,55].
4. By whom: individual differences—such as age, gender, income, and baseline stress levels—can moderate the impact of digital engagement on stress, with some groups being more susceptible than others [40-44].

By adopting this multidimensional perspective, our study seeks to bring coherence to the scattered evidence in existing research and provide a more thorough understanding of the link between digital behaviors and stress. In addition, identifying behavioral markers of stress in internet use may inform the design of future tools for real-time stress monitoring, complementing traditional self-reported measures. This approach contributes to a deeper understanding of digital well-being and supports the development of targeted interventions for healthier online behaviors.

## Methods

In this section, we describe our study design, participants, collected data, extracted features, and analysis models that allowed us to overcome the challenges of previous work described in the “Contributions and Impact of Our Study” section.

### Study Design

We conducted a longitudinal multimodal study over 7 months that combined passively collected fine-grained web browsing traces with repeated monthly online questionnaires (as provided in Figure 1). The web browsing traces for desktop users included URL-level traces, while for mobile users, both URL-level and application-level traces were included (throughout the paper, we will use “app” to mean mobile app). We measured the perceived stress of our panelists using the validated Perceived Stress Scale (PSS)-10 in 6 monthly waves. In the first wave, we also collected the sociodemographic characteristics of the panelists, including age, gender, and income.

### Participants

The study was conducted on a sample of German internet users, recruited through a General Data Protection Regulation–compliant panel company (Bilendi GmbH), which provided access to participants who had already installed tracking software on their devices to capture their internet use. The company also managed survey coding and distribution, sending email notifications to relevant participants each time a survey was launched. Participation was voluntary; panelists were informed about the study and chose to take part. Compensation ranged from €1‐€3 (US $1.10-US $3.30) US $3.30) per month, depending on the number of devices tracked, plus an additional amount based on the company’s standard rate (€6/h or US $6.60/h) for each survey completion. All currency values in this study are reported in euros. The exchange rate at the time of the study was €1=US $1.10. All panelists from the company were invited via email for the first wave of questionnaires, yielding 1490 completed responses. In the subsequent 5 waves, which were approximately 1 month apart, all these 1490 respondents were invited via email to participate in each wave. Across all waves, the average range between the earliest and latest survey completion dates was 15 days, and the average completion time for the baseline survey was about 37 minutes (90% CI 32.7-42.3), while for the remaining 5 waves, it was approximately 19.6 minutes (90% CI 17.8-21.5). Table 1 reports the number of participants with completed responses for each wave that we retained for further analysis. We excluded 1 panelist who reported their gender as nonbinary and 52 panelists who reported their income as “other,” because these categories had too few respondents. The sample for Wave 1 is therefore 1437. We observe that about 23% of the panelists dropped out by the sixth wave. In Table 1, we also depict the distribution of the panelists across age, gender, and income for the 6 waves of questionnaires.

Next, we examined how closely our sample’s sociodemographic distributions match the German population margins for gender, age, and income (provided in Table 2). We observe that our sample’s gender distribution matches closely with that of the German population (Destatis [56]). However, for both age and income, the middle ranges are overrepresented in our sample, while the extremes are underrepresented.

**Table 1.** Descriptive characteristics of participants across 6 survey waves. The table presents the number of participants, gender distribution, age groups, income categories, and mean perceived stress scores (with SDs) for each wave. Percentages are shown in parentheses for categorical variables.

Wave	1	2	3	4	5	6
Participants (n)	1437	1314	1212	1198	1205	1107
Gender, n (%)						
Male	738 (51.35)	688 (52.36)	639 (52.72)	635 (53.01)	628 (52.12)	593 (53.57)
Female	699 (48.65)	626 (47.64)	573 (47.28)	563 (46.99)	577 (47.88)	514 (46.43)
Age group (years), n (%)						
18‐30	119 (8.28)	101 (7.67)	91 (7.51)	84 (7.01)	92 (7.63)	76 (6.67)
31‐45	462 (32.15)	414 (31.51)	385 (31.77)	382 (31.89)	379 (31.45)	330 (29.81)
46‐60	569 (39.60)	528 (40.18)	483 (39.85)	483 (40.32)	483 (40.08)	460 (41.55)
>60	287 (19.97)	271 (20.62)	253 (20.87)	249 (20.78)	251 (20.83)	241 (21.77)
Income (euros/month), n (%)						
<1000 (Tier I)	126 (8.77)	119 (9.06)	116 (9.57)	110 (9.18)	103 (8.55)	94 (8.49)
1000‐2000 (Tier II)	300 (20.88)	271 (20.62)	244 (20.13)	244 (20.37)	249 (20.66)	240 (21.68)
2001‐3000 (Tier III)	364 (25.33)	339 (25.80)	319 (26.32)	310 (25.88)	309 (25.64)	281 (25.38)
3001‐4000 (Tier IV)	294 (20.46)	271 (20.62)	243 (20.05)	245 (20.45)	251 (20.83)	229 (20.69)
>4000 (Tier V)	353 (24.57)	314 (23.90)	290 (23.93)	289 (24.12)	293 (24.32)	263 (23.76)
Perceived Stress Score, mean (SD)	16.19 (7.19)	15.83 (7.43)	15.76 (7.56)	15.65 (7.41)	15.54 (7.50)	14.89 (7.58)

**Table 2.** Distribution of the adult population in Germany (2023) by gender, age group, and monthly income level.

Category	Adult population, n (%)
Sex	
Male	41.2 (48.8)
Female	42.3 (51.2)
Age group (years)	
18‐30	14.2 (17)
31‐45	19.2 (23)
46‐60	21.7 (26)
>60	28.4 (34)
Monthly income level (euros)	
<€1250 (<US $1375)	21.1 (25.3)
€1250-€2080 (US $1375-US $2288)	13 .7 (16.4)
€2080-€2920 (US $2288-US $3212)	12.4 (14.8)
€2920-€4170 (US $3212-US $4587)	13.6 (16.3)
>€4170 (>US $4587)	22.6 (27.1)

### Data Collection

The panelists of the panel company had already consented to install tracking software on their desktops or mobile devices. Some participants consented to install it on both devices. Through this tracking software, the company provided fine-grained traces of visited URLs and mobile apps, including the time of visit and duration of each visit. During the 7 months, we recorded 47,100,701 URL visits from both desktop and mobile users, covering 236,955 unique web domains. For mobile apps, we captured 13,553,645 app visits across 13,476 unique apps.

### Data Cleaning and Preprocessing

First, we removed the bottom 20% of panelists in each wave, ranked by total time spent browsing, since they did not have sufficient data to extract meaningful internet use patterns. Second, we identified a group of panelists who appeared to be “professional survey takers,” spending more than 25% of their online time on survey domains. To focus on users with more typical internet use, we excluded these individuals from the sample. Notably, applying the above time threshold to their nonsurvey activities would have led to the removal of more than 29% of these panelists. Finally, to ensure that our internet use measures accurately capture user behavior, we only included panelists for whom we could categorize at least 80% of their web visits (refer to the “Data Enrichment” section for details). Table 3 summarizes the number of participants excluded at each step, resulting in a set of distinct panelists across waves comprising 656 mobile users and 526 desktop users. Table 4 presents the sociodemographic characteristics of the remaining users included in the analysis. In the mobile data, the proportion of users aged 31‐45 years increased compared to the baseline questionnaire, as provided in Table 3. In the desktop data, the proportion of males and users aged 46‐60 years increased, while the proportion of users aged 31‐45 years decreased relative to the baseline questionnaire.

**Table 3.** Overview of panelists with matched passive web data from desktop and mobile devices across 6 survey waves. The table shows the number of users before and after data cleaning for both device types. The final row indicates the total number of distinct users retained in the cleaned dataset.

Survey wave	Number of panelists	Desktop users	Desktop users (cleaned)	Mobile users	Mobile users (cleaned)
1	1437	981	359	907	519
2	1314	848	321	806	470
3	1212	762	284	728	426
4	1198	717	257	717	418
5	1205	714	265	697	399
6	1107	656	227	649	368
Total distinct users	—^a^	—	526	—	656

a Not applicable.

**Table 4.** Sociodemographic characteristics of users included in the analysis after data cleaning for both mobile and desktop datasets.

Characteristic	Mobile (n=656)	Desktop (n=526)
Gender, n (%)		
Male	334 (50.91)	289 (54.94)
Female	322 (49.09)	237 (45.06)
Age group (years), n (%)		
18‐30	53 (8.08)	38 (7.22)
31‐45	246 (37.5)	137 (26.05)
46‐60	247 (37.65)	232 (44.11)
>60	110 (16.77)	119 (22.62)
Income (euros/month), n (%)		
<1000 (Tier I)	60 (9.15)	59 (11.22)
1000‐2000 (Tier II)	129 (19.66)	121 (23)
2001‐3000 (Tier III)	166 (25.30)	128 (24.33)
3001‐4000 (Tier IV)	137 (20.88)	87 (16.54)
>4000 (Tier V)	164 (25)	131 (24.9)

### Data Enrichment

To understand “how” the panelists are using the internet, we categorized their online visits into semantic categories. The goal was to group domains, subdomains, and apps based on their primary function into categories such as “social media” (eg, facebook.com [Meta Platforms, Inc] and TikTok app [ByteDance]) and “productivity” (eg, Gmail [Google LLC] and calendar.google.com [Google LLC]). We derived the set of categories (provided in Table 5) by combining categories used by app stores and web domain classification services such as Webshrinker.com. For platform domains such as google.com, we also categorized their subdomains. For instance, google.com was categorized as “search,” while mail.google.com was classified as “productivity.” Table 5 provides the complete list of semantic categories we considered, along with some examples. Two researchers from our team first independently annotated the categories for all domains and apps that constituted around 85% of web visits made by our panelists. Later, disagreements were resolved collaboratively. We observed a substantial interannotator agreement with a Cohen ĸ agreement [57] score of 0.7, based on annotations for a random subset of 200 domains. Following this process, we classified 3777 domains and 989 apps into semantic categories, capturing 85% of visits from mobile devices and 84% from desktops.

**Table 5.** Categorization of web domains and mobile apps based on semantic usage type. The table lists representative examples of domains and apps across various categories, grouped separately for desktop web domains and mobile apps.

Category	Example of domains, subdomains, and apps in the category
Domains	
Entertainment	youtube.com, twitch.tv, disneyplus.com, and netflix.com
Shopping	amazon.de, ebay.de, kleinanzeigen.de, and temu.com
Social media	facebook.com, twitter.com, and instagram.com
Messaging	whatsapp.com, knuddels.de, and fdating.com
Productivity	mail.google.com, outlook.live.com, navigator.web.de, and docs.google.com
Games	gameduell.de, anocris.com, forgeofempires.com, and spielaffe.de
Adult	pornhub.com, xvideos.com, xnxx.com, and romeo.com
News	bild.de, focus.de, welt.de, and wunderweib.de
Apps	
Entertainment	YouTube (Google LLC), Netflix (Netflix, Inc), and Spotify Music (Spotify Technology)
Shopping	Amazon Shopping (Amazon.com, Inc), eBay (eBay, Inc), Vinted.fr (Vinted Group)**,** and Lidl Plus (Schwarz Group)
Social media	Facebook (Meta Platforms, Inc), Instagram (Meta Platforms, Inc), and Twitter (X Corp), TikTok – Make Your Day (ByteDance)
Messaging	WhatsApp (Meta Platforms, Inc), Facebook Messenger (Meta Platforms, Inc), and Telegram (Telegram FZ-LLC)
Productivity	Gmail (Google LLC), GMX Mail (Global Mail eXchange), WEB.DE Mail (United Internet Group), and Google Calendar (Google LLC)
Games	Candy Crush Saga (King), Coin Master (Moon Active), Royal Match (Dream Games), and Pokémon GO (Niantic)
News	n-tv Nachrichten (RTL Group), kicker online (Olympia -Verlag GmbH), AOL – News (AOL Media LLC), and BILD: Immer aktuell informiert (Axel Springer SE)

### Measures

As described in the “Study Design” section, we combined repeated monthly stress questionnaires with web browsing data. Perceived stress was measured through PSS-10 questionnaire responses, and internet use features were derived from passively collected web traces. For each panelist in each survey wave, we calculated internet usage features based on their activity during the period “T” preceding the stress measurement (ie, the time of questionnaire response). To address the question of “when” the internet is used, we extracted features for either 30 or 2 days to examine both long-term and short-term effects. The resulting measures were then used to examine the associations between internet use and stress.

#### Measures From Web Traces

To measure “how” individuals use the internet, we created features that span from coarse- to fine-grained measures, as provided in Table 6. We captured overall web activity at the coarse-grained level, such as total time spent online. We also accounted for the time of the day when panelists were browsing the web by including the difference between the time spent online during daytime (6 AM-6 PM) and nighttime (6 PM-6 AM) hours. At a finer granularity, we analyzed how panelists distributed their time across online activities such as social media, entertainment, and news. For each survey wave, if a participant completed the survey on a given date (eg, July 31), we summed their time spent on each activity over the period (T=30 or 2 days; eg, July 1‐30 or July 29‐30) preceding the day of completing the survey. For instance, time spent on news represents the aggregated time on news domains (desktop) and news domains and apps (mobile) during that period. Each participant had up to 6 time points, 1 per wave.

**Table 6.** Features and their descriptions. Time spent online is measured in hours in the period T (30 days or 2 days) before the measurement of stress. These features are computed for online activity on each device (desktop or mobile) separately.

Features	Description
Coarse-grained	
Total time spent online	Total time spent online in period T
Daytime nighttime difference	Difference of time spent online during daytime (6 AM-6 PM) and nighttime hours (6 PM-6 AM)
Fine-grained	
Time spent on entertainment Time spent on social media Time spent on messaging Time spent on news Time spent on adult content Time spent on games Time spent on shopping Time spent on productivity	Time spent on different semantic classes of online activities. For instance, time spent on entertainment domains or apps such as YouTube.com or Amazon Prime is classified as entertainment use.
Control variables	
Gender Age Income Survey wave	Sociodemographic characteristics of individuals and seasonality

#### Measures From Questionnaires

We used the PSS-10 [58] in our monthly questionnaires to measure the stress levels of our panelists. The PSS-10 is a widely used, validated scale designed to assess how stressed individuals feel. It captures aspects such as the unpredictability of life, perceived control over situations, and general stress levels over the past month. Participants rate their responses on a scale from 0 (never) to 4 (very often), producing a total score between 0 and 40 across the 10 items. Higher scores indicate greater perceived stress, with scores typically grouped into 3 levels: 0‐13 (low stress), 14‐26 (moderate stress), and 27‐40 (high stress) [59,60]. In addition, we collected each participant’s self-reported sociodemographics, including age, gender, and income, in the first wave of the questionnaires.

### Statistical Analysis

We used linear mixed-effects models (LMMs) [61] to examine the relationship between internet use and stress. We chose LMMs for analyzing data from our longitudinal study since they account for repeated measurements of individuals and incorporate fixed and random effects. Fixed effects included internet use features provided in Table 6. Random intercepts were added to account for individual-specific differences in baseline stress levels across participants.

We formally describe the models as follows. For an individual i at questionnaire wave j∈ {1,2...6}, we denote $Y_{ij}$ as the variable of interest, $x_{ij}$ as the covariate, and the intercept for the random effect as $u_{j}$. Therefore, we consider the following LMM:


$$Y_{ij}=\beta_{0}+\beta_{1}x_{ij1}+\beta_{2}x_{ij2}+\ldots +\beta_{n}x_{ijn}+u_{j}+\epsilon_{ij}$$

where:

$Y_{ij}$ is the perceived stress level of the individual i measured at the questionnaire wave j.
$\beta_{0}$ is the fixed intercept.
$\beta_{1},\ldots ,\beta_{n}$ are the fixed effect coefficients for each covariate $x_{ijn}$.
$x_{ij1}$=(total time spent online, $x_{ij2}$=daytime-nighttime difference, $x_{ij3}$=time spent on entertainment ... $x_{ijn}$=survey wave), where $x_{ijn}$ corresponds to each feature provided in Table 6.
$u_{j}$ is the random effect for individual i, capturing individual-level variability.
$\epsilon_{ij}$ is the residual error term for the individual i at wave j.

We conducted model diagnostics to validate the assumptions of LMMs, including checks for multicollinearity (Variance Inflation Factor (VIF) <2.0). All statistical analyses were implemented using Python’s statsmodels package (version 0.14.1; Python Software Foundation*)* [62].

To understand whether the granularity of the extracted features affects model performance and the associations identified, we developed 2 models. The first model (Model 1) focused on coarse-grained measures of internet use such as total time spent online and daytime-nighttime difference. The second model (Model 2) extended the first model and also incorporated finer-grained measures of internet use across semantic classes. For Model 2, we dropped the total time spent online feature to avoid multicollinearity.

Previous work has shown that both sociodemographics [40-44] and seasonal variations [63,64] can significantly influence individuals’ stress levels. Accordingly, we included the sociodemographics and seasonality measures as control variables for both models, as provided in Table 6.

### Ethical Considerations

Our study was approved by Aalto University’s Research Ethics Committee (approval ID D/894/03.04/2023). Data collection was conducted via a General Data Protection Regulation–compliant European company, and informed consent was obtained from participants for both the surveys and web-trace datasets, with the option to withdraw consent at any time during or after the study. To protect participants’ privacy, we implemented strict data privacy measures. The web dataset was anonymized by the panel company by removing personal information such as email addresses and usernames to prevent participant identification. In addition, the dataset was stored and analyzed solely on the university’s secure server, with access restricted to the research team. We will make the anonymized data and code available to support the open-source community and to spur further research at the intersection of internet use and well-being.

## Results

### Overview

Our study examined various internet use behaviors associated with stress, and in this section, we present our results across 4 key contextual dimensions (as outlined in the “Contributions and Impact of Our Study” section). We first analyzed “how*”* internet-based features relate to stress. We then explored the remaining dimensions: device-based differences (where) by comparing desktop and mobile usage, temporal patterns (when) using internet activity from the 2 days before the survey, and individual differences (by whom) through subgroup analyses based on age, gender, income, and baseline stress levels.

### Behavioral Patterns (How)

To understand how internet usage is associated with stress, we ran LMMs on 2 sets of features, progressing from coarse-grained (amount and timing of usage) to fine-grained (also including semantic category usage) measures. An ANOVA test was conducted to determine whether the more complex model explained significantly more variance than the simpler model. The results showed no statistically significant improvement when using the more complex model, although the more complex model provided important information on the nuanced relationship between internet use and stress.

Analysis of 30-day mobile data (number of panelists, N=656) and observations, n=2600), as provided in Table 7, revealed that Model 2—which includes both timing and semantic category usage—identified significant associations with stress. Specifically, shopping-related usage was positively associated with stress (β=.04,, 95% CI 0.00‐0.08; *P*=.04), while productivity usage showed a negative association (β=–.03, 95% CI −0.06 to −0.00; *P*=.04). In contrast, Model 1, which included only total usage and timing, did not show any significant associations.

**Table 7.** Results from linear mixed-effects models for all participants, based on 30-day mobile data. Model 1 includes coarse-grained features, while Model 2 incorporates fine-grained usage categories (described in the “Statistical Analysis” section). Estimates, CIs, and *P* values are reported for each predictor. Statistically significant *P* values are in bold.

Predictors	Model 1^a^, estimate (95% CI)	*P* value	Model 2^b^, estimate (95% CI)	*P* value
Intercept	20.77^c^ (19.09‐22.44)	<.001	20.69^c^ (19.01‐22.37)	<.001
Survey wave	−0.10^d^ (−0.19 to −0.02)	.01	−0.11^d^ (−0.19 to −0.02)	.01
Gender	1.79^c^ (0.80‐2.77)	<.001	1.68^c^ (0.68‐2.68)	<.001
Age	−1.62^c^ (−2.20 to −1.04)	<.001	−1.57^c^ (−2.16 to −0.99)	<.001
Income	−1.11^c^ (−1.50 to −0.73)	<.001	−1.08^c^ (−1.47 to −0.69)	<.001
Total time spent online	0.00 (−0.00 to 0.01)	.39	—^e^	—
Daytime nighttime difference	−0.01 (−0.02 to −0.00)	.12	−0.01 (−0.02 to 0.00)	.18
Time spent on entertainment	—	—	0.01 (−0.01 to 0.02)	.37
Time spent on social media	—	—	0.00 (−0.01 to 0.02)	.71
Time spent on messaging	—	—	0.00 (−0.02 to 0.02)	.70
Time spent on games	—	—	0.00 (−0.00 to 0.01)	.30
Time spent on shopping	—	—	0.04^d^ (0.00‐0.08)	.04
Time spent on productivity	—	—	−0.03^d^ (−0.06 to −0.00)	.04
Time spent on news	—	—	−0.03 (−0.09 to 0.03)	.33

a σ²=11.67; τ₀₀=36.54_pid_; ICC=0.76; N=656_pid_; Observations=2600.

b σ²=11.65; τ₀₀=36.48_pid_; ICC=0.76; N=656_pid_; Observations=2600.

c *P*<.001.

d *P*<.05.

e Not applicable.

In addition, sociodemographic factors such as age, gender, and income consistently predicted stress across both models. Age (β=–1.57, 95% CI −2.16 to −0.99; *P*<.001) and income (β=–1.08, 95% CI −1.47 to −0.69; *P*<.001) were negatively associated with stress, while women reported higher stress levels (β=1.68, 95% CI 0.68‐2.68; *P*=.001).

### Device Matters (Where)

To observe device differences, we analyzed 30-day desktop data. For desktop data (N=526 and n=1713), the results are shown in Table 8. Model 2, which incorporates semantic and temporal features, showed a weaker positive association between shopping usage and stress (β=.03, 95% CI −0.0 to 0.06; *P*=.09). As observed with mobile data, the simpler Model 1 did not reveal any significant associations with internet usage features. Similarly, the sociodemographic results were consistent with those observed in the mobile data.

**Table 8.** Results from linear mixed-effects models for all participants, based on 30-day desktop data. Model 1 includes coarse-grained features, while Model 2 incorporates fine-grained usage categories (described in the “Statistical Analysis” section). Estimates, CIs, and *P* values are reported for each predictor. Statistically significant *P* values are in bold. Random effects, intraclass correlation coefficient (ICC), number of participants (N), and total observations are also provided.

Predictors	Model 1^a^, estimate (95% CI)	*P* value	Model 2^b^, estimate (95% CI)	*P* value
Intercept	20.62^c^ (18.70‐22.55)	<.001	20.49^c^ (18.58‐22.41)	<.001
Survey wave	−0.14^d^ (−0.24 to −0.03)	.009	−0.15^d^ (−0.26 to −0.04)	.006
Gender	1.83^d^ (0.66‐3.00)	.002	1.73^d^ (0.55‐2.90)	.004
Age	−1.72^c^ (−2.41 to −1.04)	<.001	−1.74^c^ (−2.43 to −1.05)	<.001
Income	−0.94^c^ (−1.38 to −0.51)	<.001	−0.92^c^ (−1.36 to −0.48)	<.001
Total time spent online	−0.00 (−0.01 to 0.00)	.40	—^e^	—
Daytime nighttime difference	0.00 (−0.01 to 0.01)	.46	0.00 (−0.01 to 0.01)	.46
Time spent on entertainment	—	—	−0.00 (−0.01 to 0.01)	.64
Time spent on adult content	—	—	−0.01 (−0.03 to 0.00)	.11
Time spent on social media	—	—	0.00 (−0.02 to 0.02)	.94
Time spent on messaging	—	—	0.01 (−0.04 to 0.06)	.61
Time spent on games	—	—	0.00 (−0.03 to 0.03)	.84
Time spent on shopping	—	—	0.03 (−0.00 to 0.06)	.09
Time spent on productivity	—	—	−0.00 (−0.03 to 0.02)	.86
Time spent on news	—	—	−0.02 (−0.06 to 0.02)	.28

a σ²=11.10; τ₀₀=40.62_pid_; ICC=0.79; N=526_pid_; Observations=1713.

b σ²=11.09; τ₀₀=40.77_pid_; ICC=0.79; N=526_pid_; Observations=1713.

c P<.001

d *P*<.01.

e Not available.

### Time Period of Data (When)

To investigate the relationship between short-term versus long-term internet usage patterns and stress, we analyzed associations between various online activities performed on mobile and desktop devices in 2 time periods—30 days and 2 days—and individual stress levels. We used the same features and models as in our previous 30-day data analyses. Here, we specifically focused on web activity recorded during the 2 days immediately preceding the PSS-10 survey.

For both mobile and desktop data (as provided in Tables S1 and S2 in Multimedia Appendix 1), news usage showed a negative association with stress (β=–.54, 95% CI −1.08 to 0.00; *P*=.048) and (β=–.50, 95% CI −0.90 to −0.11; *P*=.01), respectively, in Model 2. In addition, in desktop data, messaging usage demonstrated a weak negative association with stress (β=–.59, 95% CI −1.24 to 0.06; *P*=.07) in model 2.

### Individual Differences (By Whom)

To explore how internet usage varies by individual characteristics, we conducted subgroup analyses, running models separately for categories such as gender (male and female) to understand how associations differ based on these characteristics. In the following subsections, we first examined the relationship between internet use and stress based on baseline stress levels by analyzing high-stress and low-stress groups. We then explored differences by gender, age, and income categories.

### Stress Levels

We identified 2 groups of panelists from our data—high-stress and low-stress—based on their reported PSS-10 scores in the online questionnaires. Panelists who scored more than 26 in any wave they participated in were included in the high-stress group, and panelists who scored below 14 in any wave were included in the low-stress group.

For the high-stress population (PSS-10 score > 26), several notable results were observed (as provided in Tables S3-S6 in Multimedia Appendix 1). In the 30-day mobile data, time spent (β=.01, 95% CI 0.0‐0.02; *P*<.001) in Model 1, and social media usage (β=.02, 95% CI 0.0‐0.03; *P*=.02), and gaming usage (β=.01, 95% CI 0.0‐0.02; *P*=.02) in Model 2 were positively associated with stress. In the 2 days of data, the daytime-nighttime difference showed a weak positive association (β=.11, 95% CI −0.01 to 0.23, *P*=.08) in Model 1. For desktop data, no significant variables, including sociodemographics, were found to be associated with stress in the high-stress subgroup.

In the low-stress population (PSS-10 score <14 in the baseline survey), as provided in Tables S7-S10 in Multimedia Appendix 1, adult-content usage was negatively associated with stress in 30-day desktop data (β=–.02, 95% CI −0.04 to 0.0; *P*=.07). Similarly, for the low-stress group, in the 2 days data, time spent (β=–.07, 95% CI −0.14 to 0.0; *P*=.04) was significant for desktop, while gaming usage was weakly significant for mobile data (β=.08, 95% CI −0.01 to 0.17; *P*=.1).

When analyzing the 30-day data for all participants, sociodemographic factors were strongly associated with stress in both mobile and desktop settings. However, within the high-stress group, income was the only sociodemographic variable that remained significant in mobile data, showing a negative association with stress in both the general population (β=–1.08, 95% CI −1.47 to −0.69; *P*<.001) and the high-stress subgroup (β=–.52, 95% CI −0.91 to −0.12; *P*=.01). In contrast, gender and age—which were significant predictors in the overall population—did not show statistical significance in the highly stressed subgroup for either mobile or desktop data.

### Gender Differences

Subgroup analysis by gender revealed distinct patterns in feature significance for both desktop and mobile data. In the 30-day mobile data (as provided in Table S11 in Multimedia Appendix 1), shopping usage (β=.07, 95% CI 0.01‐0.14; *P*=.02) and productivity features (β=–.05, 95% CI −0.09 to −0.01; *P*=.02) were significant only for male users (N=334 panelists and n=1334 observations) in Model 2. In contrast, these features were not significant for female users (N=322 panelists and n=1266 observations) as provided in Table S12 in Multimedia Appendix 1. No features were significant for males and females in the 30-day desktop data.

In the 2-day data (as provided in Tables S15-S16 in Multimedia Appendix 1), news consumption was negatively associated with stress for males in both desktop (β=–.52, 95% CI −1.02 to −0.01; *P*=.04) and mobile (β=–.58, 95% CI −1.23 to 0.07; *P*=.08) data. For females, in mobile data, daytime-nighttime difference (β=.10, 95% CI −0.0 to 0.21; *P*=.06) and messaging (β=.23, CI −0.02 to 0.48; *P*=.08) were weakly positively associated.

### Age Differences

Subgroup analysis by age revealed distinct patterns in web-based associations. For the 30-day mobile data (as provided in Tables S19-S22 in Multimedia Appendix 1), shopping was positively associated with stress in age groups of 18‐30 years (β=.12, 95% CI −0.02 to 0.25; *P*=.09) and older than 60 years (β=.01, 95% CI 0.02‐0.19; *P*=.02). In the age group of 30‐45 years, weak positive associations were found for entertainment (β=.02, 95% CI −0.00 to 0.04; *P*=.08) and messaging (β=.03, 95% CI −0.00 to 0.06; *P*=.07), whereas productivity was negatively associated (β=–.08, 95% CI −0.14 to −0.02; *P*=.007). In the older than 60 years group, time spent (β=.01, 95% CI 0.00‐0.03; *P*=.03) and messaging (β=.05, CI −0.00 to 0.09; *P*=.06) were positively associated, while the daytime-nighttime difference (β=–.03, 95% CI −0.05 to −0.01; *P*=.01) and shopping (β=.10, 95% CI 0.02‐0.19; *P*=.02) were negatively associated.

In the 30-day desktop data (as provided in Tables S23-S26 in Multimedia Appendix 1), adult content usage was negatively associated in 18‐30 years (β=–.87, 95% CI −1.77 to 0.03; *P*=.06) and older than 60 years (β=–.19, 95% CI −0.38 to 0.00; *P*=.06) age groups. In addition, in the 18‐30 age group, the daytime-nighttime difference (β=.07, CI −0.00 to 0.14; *P*=.06) was positively associated, while news usage (β=.026, CI −0.42 to −0.10; *P*=.002) was negatively associated. Shopping was positively associated in the age group of 45‐60 years (β=.04, 95% CI −0.00 to 0.09; *P*=.06).

For the 2-day mobile data (as provided in Tables S27-S30 in Multimedia Appendix 1), news was negatively associated in both the 30‐45 years (β=–1.01, 95% CI −2.22 to 0.19; *P*=.10) and 45‐60 years (β=–.85, 95% CI −1.76 to 0.06; *P*=.07) age groups. In addition, in the age group of 30‐45 years, the daytime-nighttime difference (β=.14, 95% CI 0.02‐0.25; *P*=.02) and entertainment usage (β=.22, 95% CI 0.01‐0.43; *P*=.04) were positively associated, whereas shopping was negatively associated (β=–.54, 95% CI −1.08 to −0.0; *P*=.05). In the older than 60 years of age group, time spent on gaming (β=.26, 95% CI 0.01‐0.50; *P*=.04) was positively associated.

Similarly, for the 2-day desktop data (as provided in Tables S31-S34 in Multimedia Appendix 1), messaging was negatively associated in the 45‐60 years age group (β=–.78, 95% CI −1.48 to −0.07; *P*=.03), but positively associated for the older than 60 years age group (β=7.63, 95% CI 0.39‐14.87; *P*=.04). In the age group of 18‐30 years, entertainment (β=1.14, 95% CI −0.18 to 2.46; *P*=.09) was positively associated, and social media (β=1.22, 95% CI 0.15‐2.29; *P*=.03) was positive for the 30‐45 age group. In addition, time spent (β=–.14, 95% CI 0.28‐0.0; *P*=.06) and news usage (β=.46, 95% CI −0.96 to 0.04; *P*=.07) were negatively associated in the older than 60 years age group.

### Income Differences

For the 30-day mobile data (as provided in Tables S35-S39 in Multimedia Appendix 1), messaging (β=–.06, 95% CI −0.12 to −0.01; *P*=.03) was negatively associated with stress in participants earning less than €1000 (US $ 1100) per month (Tier I). In the €2001-€3000 (US $2201.10-US $3300) income group (Tier II), productivity (β=–.05, 95% CI −0.10 to 0.0; *P*=.06) and news usage (β=–.10, 95% CI −0.20 to 0.01; *P*=.08) were both negatively associated. Time spent (β=.01, CI 0.0‐0.02; *P*=.02) and shopping (β=.08, 95% CI 0.01‐0.16; *P*=.02) were positively associated with stress for participants earning €3001-€4000 (US $3301.10-US $4400; Tier IV). No other significant internet-based features were observed for other income categories.

For the 30-day desktop data (as provided in Tables S40-S44 in Multimedia Appendix 1), news usage was positively associated with stress in Tier I income group (β=.28, 95 CI 0.05‐0.50; *P*=.02), while productivity was negatively associated (β=–.07, 95% CI −0.13 to −0.01; *P*=.03). For participants in Tier III income, news usage (β=–.13, 95% CI −0.25 to 0.0; *P*=.06) was negatively associated. For participants in Tier IV income, shopping was positively associated with stress (β=.08, 95% CI 0.02‐0.15; *P*=.02). For Tier V participants, social media use was positively associated with stress (β=.06, 95% CI 0.01‐0.12; *P*=.03), while news use (β=–.16, 95% CI −0.25 to −0.07; *P*<.001) and time spent were negatively associated (β=–.01, 95% CI −0.03 to 0.0; *P*=.03). No significant associations were identified for other income categories.

For the 2-day mobile data (as provided in Tables S45-S49 in Multimedia Appendix 1), gaming (β=.21, 95% CI −0.02 to 0.43; *P*=.07) was positively associated in the Tier II income group and negatively associated (β=–.19, 95% CI −0.37 to 0.0; *P*=.047) in the Tier V income group. News use was negatively associated in the Tier III (β=–.75, 95% CI −1.58 to 0.09; *P*=.08) and Tier V (β=–.74, 95% CI −1.60 to 0.12; *P*=.09) income groups. In addition, messaging (β=, 95% CI 0.14‐0.82; *P*=.006) was positively associated in the Tier III income group, and daytime-nighttime difference (β=.18, 95% CI 0.0‐0.36; *P*=.04) was positively associated in the Tier IV income group.

For the 2-day desktop data (as provided in Tables S50-S54 in Multimedia Appendix 1), news usage was negatively associated in the Tier III income group (β=–1.13, 95% CI −2.33 to 0.06; *P*=.06) and the Tier V income group (β=–1.04, 95% CI −1.76 to −0.32; *P*=.005). In addition, in the Tier V income group, productivity (β=.64, 95% CI 0.07‐1.21; P=.03) was positively associated with stress. In the Tier IV income group, time spent (β=.23, 95% CI 0.04‐0.42; *P*=.0192) and daytime-nighttime difference (β=.23, 95% CI −0.00 to 0.46; *P*=.05) were positively associated.

## Discussion

### Principal Findings

**Figure 2.**
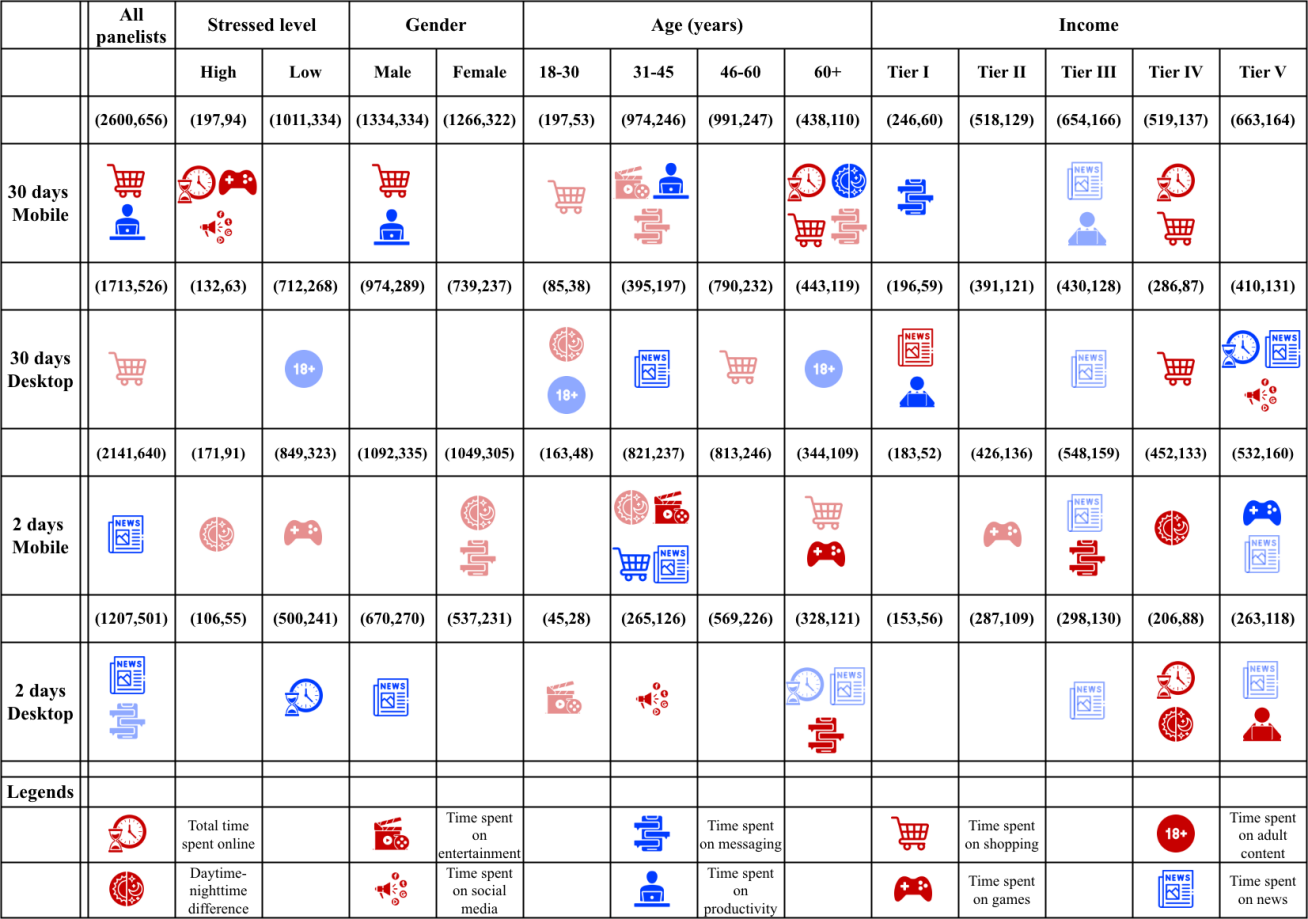
Overview of significant internet usage behaviors across various contextual dimensions. The icons represent different internet use features. The rows correspond to a combination of the time frame (30 days or 2 days) and device type (desktop or mobile), and the columns correspond to factors pertaining to individual differences (stress levels, gender, age, and income). Red icons denote positive associations, blue icons indicate negative associations, and empty cells show no significant internet features. The intensity of the color reflects the strength of the significance, with lighter icons denoting weak significance (*P*=.05 to *P*<.10) and darker icons representing high significance (*P*<.05).

Our results show that various internet usage behaviors are associated with stress, both positively and negatively. These associations differ across device type, time frame, and sociodemographic groups. Figure 2 summarizes these patterns and their variation across panelist subgroups. In the following sections, we discuss the internet use features that have shown positive (social media, entertainment, shopping, games, and time of internet use), negative (adult content, productivity, and news), and mixed (messaging and screen time) associations with stress.

### Positive Associations

#### Social Media

People spend a substantial portion of their time online on social media platforms [65]. In our dataset, social media accounted for approximately 23% of total usage in mobile data and 15% in desktop data. Therefore, understanding social media’s relationship with stress is increasingly important as it continues to occupy a large share of individuals’ internet activity.

Previous research shows that social media can contribute to stress, act as a resource, or function as a coping tool [50]. Factors such as fear of missing out [66], appearance-related pressure [67], and communication overload [68] have been linked to increased distress. At the same time, other studies have highlighted its potential to buffer stress and offer social support in specific contexts [22,35,69].

In our results, when significant, social media was consistently positively associated with stress across subgroups and device types. This association was significant for the high-stress subgroup in the 30-day mobile data, the Tier I income group in the 30-day desktop data, and the 30‐45 age group in the 2-day desktop data (Figure 2). Although we cannot definitively determine whether social media contributes to or alleviates stress, these patterns suggest that it may be used as a coping strategy for these groups.

Previous work has identified social media as a space for various coping mechanisms [35]. Our findings in the middle-aged group potentially reflect this pattern, aligning with earlier research [70], possibly due to the support accessed through these platforms. This is consistent with studies linking social media use, particularly Facebook, to support-seeking behavior [71].

Overall, our results highlight the role of social media in shaping stress experiences. While our study, combining fine-grained web data with a longitudinal design and contextual framework, strengthens this interpretation, further research is needed to disentangle the causal directions of the observed associations and social media’s role—whether as stressor, resource, or coping tool.

#### Entertainment

According to a recent survey in Germany, people spend an average of 203 minutes per day watching content online [65]. In our data, entertainment usage accounted for 9% of total usage on mobile devices and 14% on desktop devices.

Previous studies have reported a positive correlation between entertainment usage and stress [21,72-74]. Further, activities such as watching content or listening to music have also been identified as common coping strategies for managing stress [36,37,75]. In our findings, entertainment usage was positively associated with stress in both desktop and mobile data. For mobile users, this association was consistent across both the 30-day and 2-day periods for the 30‐45 age group. In the 2-day desktop data, a weak positive association was observed for the age group of 18‐30 years.

These results suggest that entertainment usage (similar to social media) may serve as a coping mechanism, especially among younger users in our data, compared with older individuals. Previous research indicates that younger individuals are more likely to engage in binge-watching as a way to regulate emotions [36]. However, there is a lack of detailed research on this association [74], and future studies should further explore the types of content consumed and their relationship to mental health.

#### Online Shopping

Online shopping has grown substantially in recent years, particularly with the rise of smartphones, which now account for 80% of all retail visits [76]. Previous studies have linked compulsive buying-shopping disorder to higher levels of stress [29,38,52,77-79]. At the same time, shopping has also been identified as a way to relieve stress [80,81].

In our results, shopping was predominantly positively associated with stress across mobile and desktop data, in various time frames and subgroups (refer to Figure 2), aligning with previous findings. A negative association was observed only for the age group of 30‐45 years in the 2-day mobile data, suggesting that, for this group, shopping may serve as a short-term stress reliever, or that individuals under stress may avoid shopping. The latter behavior aligns with previous research showing that, in middle-aged adults, stress can lead to increased saving behavior [82].

This association was more pronounced in mobile data, suggesting that people may use mobile phones to cope with stress due to their easier accessibility. It was also consistent across both mobile and desktop data for the Tier IV income group, supporting earlier findings that higher-income individuals may use shopping as a way to cope with stress [38]. In the 30-day mobile data, this positive association was observed among male participants but not among females. Previous studies show that males are more likely to experience negative emotions related to shopping [83], while some studies report no gender differences in online shopping addiction tendencies [29].

These findings highlight how shopping has become embedded in daily life and its potential influence on stress levels. Future research should explore different types of shopping (hedonic vs utilitarian) and their impact on mental health. Another direction could be to examine the time spent on shopping compared with actual purchases completed after the payment process, and how these different shopping behaviors are associated with stress levels.

#### Gaming

Gaming has become a widespread daily habit, with the global market projected to reach US $522.46 billion by 2025 [84]. Previous research presents mixed findings: while gaming has been positively linked to stress and lower psychosocial well-being, with stress being a known precursor to pathological gaming [32,33,85]. It has also been identified as a stress reliever and coping mechanism [39,86-88].

In our results, gaming usage was positively associated with stress in the 30-day mobile data for the high-stress subgroup. In the 2-day mobile data, positive associations were observed for the low-stress group, users aged older than 60 years, and those in the Tier II income range. A negative association was found for the Tier V income group. These findings align with previous research suggesting that gaming may intensify stress in already stressed individuals [89], whereas older adults often use gaming as a way to relieve stress [90]. The contrasting patterns across income groups may reflect differences in usage intensity, as lower-income individuals tend to engage more frequently in gaming than those with higher incomes [91].

We did not find any significant associations in the desktop data, which may be due to lower gaming activity on desktop browsers compared with mobile apps—gaming accounted for only 4.7% of desktop usage versus 16.8% in mobile data. Future research should examine the type of gaming, such as games that require active cognitive engagement (eg, first-person shooter games) versus low cognitive requirement arcade games, and how these different types of games relate to stress.

#### Timing of Online Activity

Individuals tend to have preferences for the timing of both online and offline activities throughout the day [92,93]. Previous research has shown that increased nighttime smartphone use is linked to higher perceived stress [49]. Nighttime use has also been associated with reduced sleep duration [48,94] and poorer sleep quality [95,96], both of which are shown to impact mental health negatively [97-99].

In our results, the daytime–nighttime difference feature was mainly positively associated with perceived stress across various groups. A negative association was observed only in the age group of older than 60 years in the 30-day mobile data. Positive associations were more common in the shorter time frame, particularly in the 2-day mobile data, for groups including high-stress individuals, females, those aged 30‐45 years, and the Tier IV income group (refer to Figure 2).

Our results indicate that daytime internet use, compared with nighttime use, is positively associated with stress, contrasting with earlier studies that reported a stronger link between nighttime use and stress in young adults [49]. Longitudinal research has shown that it is sleep loss due to time spent online, rather than internet use itself, that is associated with poorer mental health outcomes [6]. Internet use patterns can also be influenced by individuals’ chronotypes, which in turn will affect preferred active hours [100]. Moreover, future studies could investigate panelists’ bedtime and examine postbedtime usage, as previous studies have shown that postbedtime use—rather than nighttime use in general—has the most harmful effects on sleep quality [95]. The negative association observed in the age group of older than 60 years may suggest that older individuals are more sensitive to late-night use and its link to stress. Further research is needed to better understand how daily temporal patterns of internet use relate to stress, particularly by considering users’ chronotype and bedtime.

### Negative Associations

#### Adult Content

A recent study estimates that around 90 million people may be affected by problematic adult content usage [101]. Research has shown that problematic adult content consumption can impact mental health, including associations with higher stress [102,103].

In our results, however, adult content consumption was negatively associated with stress levels. This negative relationship was observed in the low-stress group (PSS score < 14), and in the age groups of 18‐30 years and older than 60 years within the 30-day desktop data. Some studies have reported no significant link between adult content use and psychological health [104], while others suggest it is often consumed as a form of leisure [105]. Our results may suggest that adult content serves as a stress buffer for our participants, as previous research has indicated stress and stress relief as common motivations for its use [106,107]. Another possible explanation is that users with lower stress levels might engage in this activity as a form of leisure or a means to alleviate boredom [105]. Previous research has identified boredom proneness and its positive association with the frequency of pornography use [108]. However, the positive associations observed in past studies were tied to problematic adult content consumption or addiction. This implies that while limited consumption may offer stress relief, excessive use could have detrimental effects. Future research should explore both the positive and negative impacts of adult content consumption on mental health, particularly in relation to the amount of consumption.

#### Productivity

Information and communication technology has become a central part of daily work and study routines, particularly in the 21st century. Most of the work we do on information and communication technology devices is connected to the internet, and their use for work and productivity has been linked to improved workplace efficiency [109,110]. However, the effects of internet use on psychological health and stress are dual in nature. While it has been associated with increased productivity, higher internet use for work-related tasks has also been linked to higher stress levels [51,111-114]. Some interventional studies report no significant effects [113], while others suggest that using the internet for work and study can have a positive impact on mental health [6].

In our results, increased use of productivity-related apps and domains was generally associated with lower perceived stress. In the 30-day mobile data, negative associations were observed among all participants, as well as within the male subgroup, the age group of 31‐45 years, and the Tier III income group. In the 30-day desktop data, a similar negative association was found in the Tier I income group. A positive association appeared only once—in the 2-day desktop data for the Tier V income group.

Our findings suggest that stressed individuals may avoid productivity-related tasks by shifting their focus to other activities. This is supported by previous research showing a positive relationship between perceived stress and avoidant coping styles [115]. Previous research has reported a negative relationship between work-related online tasks and stress among middle-aged adults [21]. At the same time, communication overload from emails and messages has been linked to higher perceived stress in adults aged 50‐85 years [15]. In our results, we found a similar negative association between productivity-related use and stress in the age group of 31‐45 years, suggesting that increased productivity use may be linked to lower stress in this group. However, no significant association was observed in the older age group. For higher-income groups, the observed positive association may reflect greater work responsibilities that are more difficult to avoid, contributing to increased stress.

Overall, our findings highlight the need for further research into how productivity-related internet use influences stress. As previous studies have shown, avoidance behaviors can increase the risk of prolonged stress and other mental health challenges [116]. Future studies should explore how online interventions can effectively help users mitigate stress.

#### News

Informed citizens are a cornerstone of a well-functioning democracy and good governance. However, recent studies suggest that news, especially news with highly negative content, can adversely affect mental health and increase stress levels [24,117-119].

Our findings reveal a counterintuitive relationship between news engagement and stress: participants who spent more time-consuming news tended to report lower stress levels. This association was more pronounced in the 2-day data for both mobile and desktop users, and overall, it was stronger in desktop data across both time periods. One possible explanation is that individuals experiencing stress may avoid the news altogether in the short term. Previous research supports this, showing that people under stress often disengage from news consumption [120-122]. Other studies have found no significant link between news consumption and stress [123,124], while some suggest that positive and soft news content may improve mood [30] and well-being [125].

Since our news category includes a range of sources, such as entertainment, sports, and politics, it is important to consider how different types of news relate to stress in the future. Future studies could examine the effects of specific news genres, as well as the influence of low-quality news sources and misinformation on mental health. Examining how consistent exposure to different news categories, sources, and misinformation influences stress responses over time could provide insights into mental health risks and how media consumption habits shape psychological well-being.

### Mixed Associations

#### Messaging

In 2021, an estimated 3.09 billion mobile phone users accessed the top messaging apps for communication, with this number projected to reach 3.51 billion by 2025 [126]. The younger generation, in particular, remains connected with friends and family through messaging apps such as WhatsApp (Meta Platforms, Inc) and Telegram (Telegram FZ-LLC). Research has shown that messaging apps can be both positively associated with stress [7,127-130] and serve as stress reducers [131-134].

In our results, messaging usage was mostly positively associated with stress in mobile data. It was positively associated in the age groups of 30‐45 years and older than 60 years and negatively associated in the Tier I income group in the 30-day mobile data. In the 2-day mobile data, it was positively associated with females and those in the Tier III income group. In the 2-day desktop data, it was negatively associated with all participants and the age group of 45‐60 years, while it was positively associated with the age group of older than 60 years.

The key finding is that messaging use was predominantly positively associated with stress in mobile data and more negatively associated in desktop data. Previous research suggests that “checking behavior” (ie, brief and repeated usage sessions) is more common in mobile use compared to desktop devices [53], and perhaps this type of repeated checking on mobile devices adds to the stress. We found that in the age group of older than 60 years, messaging was positively associated with stress in both mobile and desktop data, which aligns with previous studies showing that older individuals experience higher stress from interpersonal communication [7]. In the Tier I income group for mobile data, messaging was negatively associated with stress. Studies show that people in lower-income groups tend to send more messages [135]. Previous research has also shown that messaging can be an effective tool for reducing depression, particularly among low-income individuals [136]. Finally, in the 2-day mobile data, messaging was positively associated with stress in females but not males, reflecting previous research suggesting that frequent messaging is linked to mental health symptoms (externalizing and inattention) in females, but not in males [137].

Our results highlight how messaging can have a dual impact on stress, depending on the device, time period, and individual differences. Future work could examine how messaging with friends and family, interactions with strangers in chatrooms, and repeated checking behavior on mobile devices relate to stress, loneliness, and overall well-being.

#### Screen Time

The internet plays a pervasive role in our daily lives, and the amount of time we spend online may have a detrimental impact on stress levels [48,138]. Research has shown that smartphone addiction is significantly linked to higher stress [139,140], and the amount of time spent online is positively associated with stress [48] and other mental health issues [141,142].

In our results, total time spent online was positively associated with stress in the 30-day mobile data, aligning with previous studies [48,139,140]. This association was significant for the high-stress group, individuals older than 60 years, and those in income Tier IV. However, the relationship was mainly negative in both the 2-day and 30-day desktop data across different groups, with the exception of a positive association for the Tier IV income group in the 2-day desktop data, which mirrored the mobile data results.

Our findings suggest that increased time spent online on mobile devices may amplify stress in different contexts. For high-stress individuals, extended mobile phone usage is positively associated with stress, likely due to the constant accessibility of smartphones, making it harder to disconnect from them. Previous studies have also found that smartphone users tend to experience higher levels of digital burnout compared to those using desktops or laptops [17]. In addition, older individuals in our study showed an increase in stress with more time spent online, consistent with previous research [141].

In contrast, desktop usage generally showed negative associations with stress, which may be due to the differences in accessibility of smartphones and desktop devices. Our data show that time spent on desktop devices has higher variability, as reflected by the higher SD (71.43 for desktop vs 66.86 for mobile) and coefficient of variation (desktop: 0.97, CI 0.93‐1.02 vs mobile: 0.70, CI 0.67‐0.73), compared to time spent on mobile devices.

Future research should further investigate device differences and the role mobile devices play in predicting stress levels.

#### User Characteristics and Stress

**Figure 3.**
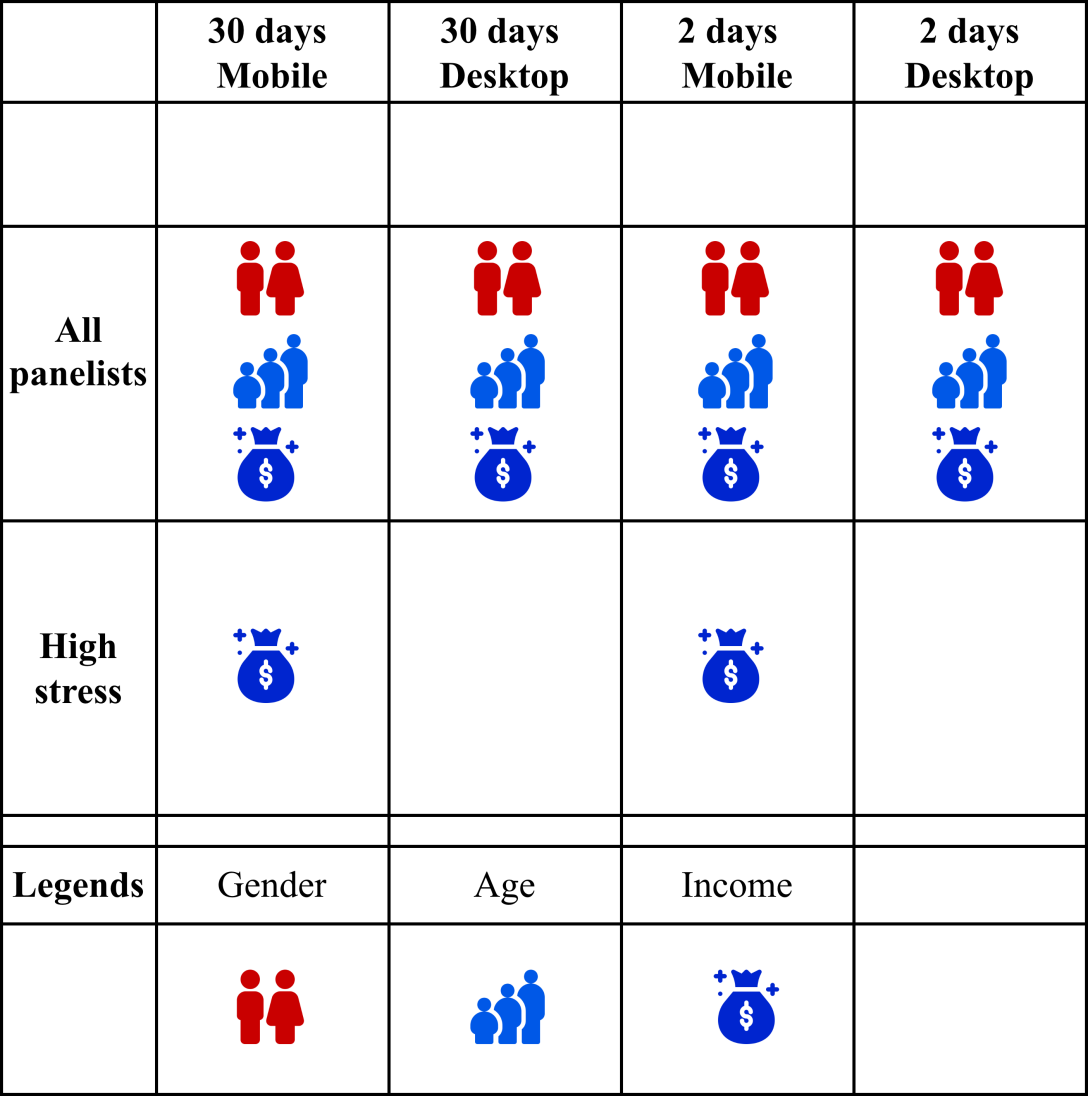
Overview of significant sociodemographic associations across time frames (30 days vs 2 days) and device types (mobile vs desktop). Rows distinguish model results between all participants and high-stress participants. Red icons indicate positive associations, blue icons indicate negative associations, and empty cells denote no significant effects.

User characteristics such as gender, age, and income influence both stress levels and their association with internet use, as provided in Figure 3. In our results, female users reported significantly higher stress than males across both mobile and desktop data and for both the 30-day and 2-day periods (refer to Figure 3). Age and income showed consistent negative associations with stress across contexts. Similar findings have been reported in earlier research on sociodemographics and stress [40-44]. Interestingly, in our analysis, income was the only sociodemographic factor significantly associated with stress in the high-stress group for mobile data, while no other sociodemographic features were significant across platforms or time frames. Previous research also suggests that income, rather than age or gender, shows the strongest association with stress [45]. This highlights the need for further investigation into the role of income in stress, as such insights can inform policies aimed at reducing income-related health disparities. Future studies should also incorporate measures to incorporate resilience of individuals (trait stress measures) [143] and personality traits alongside sociodemographics to better understand these associations.

#### Implications

Building on these findings, it is important to consider the implications for various stakeholders, including digital platform designers, health care professionals, and end-users.

For digital platform designers, these findings suggest opportunities to promote healthier use patterns. Features such as reminders to take breaks, tools to visualize usage habits, and reduced notifications during evening/nights could help users avoid stress-inducing behaviors. In addition, adaptive designs that encourage daytime work engagement over late-night use could be particularly effective. Finally, different design considerations may be needed for mobile versus desktop devices to encourage healthier internet use.

For health care professionals, understanding how digital behaviors relate to stress could offer new ways to support patients. For example, excessive gaming on mobile devices might indicate elevated stress. Health workers could include questions about internet habits in assessments and recommend tools that encourage healthier online behaviors. Notably, the low cost and high availability of web data could provide efficient tools to complement traditional monthly surveys for monitoring stress, helping to alleviate the burden on the already strained health care sector.

For individuals, these findings emphasize the importance of timing and purpose in digital habits. Being mindful of potentially stress-inducing activities, such as excessive shopping or gaming, can foster a healthier balance. Tools that track and suggest healthier patterns of internet use could assist users in managing their habits.

Overall, the relationship between stress and internet use is influenced by factors such as the type of activity, timing, and individual circumstances. This suggests that small, intentional changes in digital habits can help manage stress effectively.

### Limitations

The panelists we collected data from are gig workers who regularly participate in surveys, which may lead to behavioral differences compared to the general population. To address this issue, we used rigorous preprocessing steps, including removing users who spent more than 25% of their time on survey websites and excluding the bottom 20% of users based on time spent. Moreover, browsing data from these panels has been shown to prominently feature the most visited domains in Germany [144]. Another limitation is that users may change their online behavior when they are aware of being tracked. However, previous work has demonstrated that the privacy attitudes of web-tracked panelists are comparable to those of nonweb-tracked panelists in Germany [145], though they may vary across countries. These findings support the reliability and suitability of the web-tracking data for capturing individuals’ internet usage behavior. In addition, due to budget constraints, all panelists were from a single country, which may limit the generalizability of our findings because of cultural and behavioral differences. Previous research has demonstrated differences in cultural, social, and technological access contexts, variations in usage patterns, and mental health prevalence across countries [146,147]. Replicating this study in other countries or with a larger and more diverse sample could help further improve the generalizability and provide a broader cultural context. Further, 23% of participants dropped out between the first and final survey waves. To mitigate this attrition, we initially used a larger sample and invited all panelists from the panel company to participate in our baseline survey. Furthermore, since our tracker only tracks browsing behavior through the browser, we lack complete data on desktop usage through desktop apps, which may limit our ability to fully capture differences between mobile and desktop use. Finally, we used a validated self-report measure to assess stress, as inviting all participants to a lab setting was not feasible. Alternative approaches, such as physiological or real-time assessments, could provide more objective and detailed measures of stress.

### Conclusion

Our study examines the relationship between internet use and perceived stress through a novel contextual framework that considers how, where, when, and by whom the internet is used. The findings indicate that internet use is associated with stress, and these associations differ across various usage contexts. Specifically, engagement with social media, online shopping, entertainment, and gaming is positively linked to higher stress levels. Notably, these activities have been identified in previous research as common coping mechanisms for stress, highlighting the need for future studies to examine whether such coping strategies alleviate stress or, possibly, exacerbate it over time. In contrast, productivity-related activities, news consumption, and adult content use are negatively associated with stress, suggesting they may either function as stress buffers or indicate avoidance behavior. Associations inferred from desktop data across different contextual dimensions are weaker than those inferred from mobile data, indicating that device type plays an important role. In the short term, news consumption is negatively associated with both mobile and desktop data. For individuals already experiencing high stress, increased time online on mobile phones—particularly on social media and gaming—is correlated with higher stress levels. In addition, sociodemographic factors, especially income, have significant associations with stress. These findings have important implications for the design of digital platforms, the development of mental health interventions, and the formation of healthier online habits. Future research should focus more specifically on particular web-based behaviors, such as news consumption and online shopping, and their effects on psychological well-being. It should also aim to establish causal links between internet use and stress and further investigate the mechanisms underlying sociodemographic differences in these associations.

## Supplementary material

10.2196/78775 Multimedia Appendix 1 Mixed-effects models results across contextual dimensions.

## Abbreviations

LMM: linear mixed-effects model
PSS: Perceived Stress Scale

## References

[1] Schneiderman N, Ironson G, Siegel SD (2005). Stress and health: psychological, behavioral, and biological determinants. Annu Rev Clin Psychol.

[2] Bui T, Zackula R, Dugan K, Ablah E (2021). Workplace stress and productivity: a cross-sectional study. Kans J Med.

[3] Huang C (2010). Internet use and psychological well-being: a meta-analysis. Cyberpsychol Behav Soc Netw.

[4] Çikrıkci Ö (2016). The effect of internet use on well-being: meta-analysis. Comput Human Behav.

[5] Jenaro C, Flores N, Gómez-Vela M, González-Gil F, Caballo C (2007). Problematic internet and cell-phone use: psychological, behavioral, and health correlates. Addict Res Theory.

[6] Hökby S, Hadlaczky G, Westerlund J (2016). Are mental health effects of internet use attributable to the web-based content or perceived consequences of usage? A longitudinal study of european adolescents. JMIR Ment Health.

[7] Nimrod G (2020). Changes in internet use when coping with stress: older adults during the COVID-19 pandemic. Am J Geriatr Psychiatry.

[8] Luo Y, Yip PSF, Zhang Q (2025). Positive association between Internet use and mental health among adults aged ≥50 years in 23 countries. Nat Hum Behav.

[9] Heo J, Chun S, Lee S, Lee KH, Kim J (2015). Internet use and well-being in older adults. Cyberpsychol Behav Soc Netw.

[10] Linton MJ, Dieppe P, Medina-Lara A (2016). Review of 99 self-report measures for assessing well-being in adults: exploring dimensions of well-being and developments over time. BMJ Open.

[11] Kraut R, Burke M (2015). Internet use and psychological well-being: effects of activity and audience. Commun ACM.

[12] Yetton BD, Revord J, Margolis S, Lyubomirsky S, Seitz AR (2019). Cognitive and physiological measures in well-being science: limitations and lessons. Front Psychol.

[13] Patel VK (2019). Study of internet use characteristics, perceived stress, and internet addiction among first-year medical students of Jamnagar, Gujarat, India. Ind J Priv Psychiatry.

[14] Nikolic A, Bukurov B, Kocic I (2023). Smartphone addiction, sleep quality, depression, anxiety, and stress among medical students. Front Public Health.

[15] Reinecke L, Aufenanger S, Beutel ME (2017). Digital stress over the life span: the effects of communication load and internet multitasking on perceived stress and psychological health impairments in a German probability sample. Media Psychol.

[16] Barley SR, Meyerson DE, Grodal S (2011). E-mail as a source and symbol of stress. Organ Sci.

[17] Göldağ B (2022). An investigation of the relationship between university students’ digital burnout levels and perceived stress levels. J learn teach digit age.

[18] Becker L, Martin T, Rohleder N, Nieding G, Wannagat W (2025). Physiological stress responses to digital single- and multitasking demands in younger and older adults. Psychoneuroendocrinology.

[19] Campbell AJ, Cumming SR, Hughes I (2006). Internet use by the socially fearful: addiction or therapy?. Cyberpsychol Behav.

[20] Stanković M, Nešić M, Čičević S, Shi Z (2021). Association of smartphone use with depression, anxiety, stress, sleep quality, and internet addiction. Empirical evidence from a smartphone application. Pers Individ Dif.

[21] Khalili-Mahani N, Smyrnova A, Kakinami L (2019). To each stress its own screen: a cross-sectional survey of the patterns of stress and various screen uses in relation to self-admitted screen addiction. J Med Internet Res.

[22] Hampton K, Rainie L, Lu W, Shin I, Purcell K (2015). Psychological stress and social media use. Pew Research Center.

[23] Nygaard M, Andersen TO, Rod NH (2024). Can social connections become stressful? Exploring the link between social media use and perceived stress in cross-sectional and longitudinal analyses of 25,053 adults. J Ment Health.

[24] McLaughlin B, Gotlieb MR, Mills DJ (2023). Caught in a dangerous world: problematic news consumption and its relationship to mental and physical ill-being. Health Commun.

[25] Grubbs JB, Volk F, Exline JJ, Pargament KI (2015). Internet pornography use: perceived addiction, psychological distress, and the validation of a brief measure. J Sex Marital Ther.

[26] Bezinović P, Roviš D, Rončević N, Bilajac L (2015). Patterns of internet use and mental health of high school students in Istria County Croatia: cross-sectional study. Croat Med J.

[27] Büchler N, ter Hoeven CL, van Zoonen W (2020). Understanding constant connectivity to work: how and for whom is constant connectivity related to employee well-being?. Inf Organ.

[28] Elhai JD, Hall BJ (2016). Anxiety about internet hacking: results from a community sample. Comput Human Behav.

[29] Li H, Ma X, Fang J (2022). Student stress and online shopping addiction tendency among college students in Guangdong Province, China: the mediating effect of the social support. IJERPH.

[30] Kelly CA, Sharot T (2025). Web-browsing patterns reflect and shape mood and mental health. Nat Hum Behav.

[31] Verma G, Bhardwaj A, Aledavood T, De Choudhury M, Kumar S (2022). Examining the impact of sharing COVID-19 misinformation online on mental health. Sci Rep.

[32] Mun IB (2023). Academic stress and first-/third-person shooter game addiction in a large adolescent sample: a serial mediation model with depression and impulsivity. Comput Human Behav.

[33] Park K, Son M, Chang H, Lee SK (2024). The roles of stress, non-digital hobbies, and gaming time in adolescent problematic game use: a focus on sex differences. Comput Human Behav.

[34] Rosell J, Vergés A, Miranda-Castillo C, Sepúlveda-Caro S, Gómez M (2022). Predictors, types of internet use, and the psychological well-being of older adults: a comprehensive model. J Gerontol B Psychol Sci Soc Sci.

[35] van Ingen E, Utz S, Toepoel V (2016). Online coping after negative life events: measurement, prevalence, and relation with internet activities and well-being. Soc Sci Comput Rev.

[36] Boursier V, Musetti A, Gioia F, Flayelle M, Billieux J, Schimmenti A (2021). Corrigendum: is watching tv series an adaptive coping strategy during the COVID-19 pandemic? Insights from an Italian community sample. Front Psychiatry.

[37] Nabi RL, Torres DP, Prestin A (2017). Guilty pleasure no more: the relative importance of media use for coping with stress. Journal of Media Psychology: Theories, Methods, and Applications.

[38] Maraz A, Yi S (2022). Compulsive buying gradually increased during the first six months of the Covid-19 outbreak. J Behav Addict.

[39] Šporčić B, Glavak-Tkalić R (2018). The relationship between online gaming motivation, self-concept clarity and tendency toward problematic gaming. CP.

[40] Graves BS, Hall ME, Dias-Karch C, Haischer MH, Apter C (2021). Gender differences in perceived stress and coping among college students. PLOS ONE.

[41] Matud MP (2004). Gender differences in stress and coping styles. Pers Individ Dif.

[42] Almeida DM, Rush J, Mogle J, Piazza JR, Cerino E, Charles ST (2023). Longitudinal change in daily stress across 20 years of adulthood: results from the national study of daily experiences. Dev Psychol.

[43] Johnson MD, Krahn HJ, Galambos NL (2023). Perceived stress trajectories from age 25 to 50 years. Int J Behav Dev.

[44] de Miquel C, Domènech-Abella J, Felez-Nobrega M (2022). The mental health of employees with job loss and income loss during the COVID-19 pandemic: the mediating role of perceived financial stress. Int J Environ Res Public Health.

[45] Li R, Liu S, Huang C, Darabi D, Zhao M, Heinzel S (2023). The influence of perceived stress and income on mental health in China and Germany. Heliyon.

[46] Rus HM, Tiemensma J (2017). Social media under the skin: Facebook use after acute stress impairs cortisol recovery. Front Psychol.

[47] Yang C, Mousavi S, Dash A, Gummadi KP, Weber I (2025). Studying behavioral addiction by combining surveys and digital traces: a case study of tiktok.

[48] Thomée S, Dellve L, Härenstam A, Hagberg M (2010). Perceived connections between information and communication technology use and mental symptoms among young adults - a qualitative study. BMC Public Health.

[49] Dissing AS, Andersen TO, Jensen AK, Lund R, Rod NH (2022). Nighttime smartphone use and changes in mental health and wellbeing among young adults: a longitudinal study based on high-resolution tracking data. Sci Rep.

[50] Wolfers LN, Utz S (2022). Social media use, stress, and coping. Curr Opin Psychol.

[51] Afifi TD, Zamanzadeh N, Harrison K, Acevedo Callejas M (2018). WIRED: the impact of media and technology use on stress (cortisol) and inflammation (interleukin IL-6) in fast paced families. Comput Human Behav.

[52] Thomas TA, Schmid AM, Kessling A (2024). Stress and compulsive buying-shopping disorder: a scoping review. Compr Psychiatry.

[53] Oulasvirta A, Rattenbury T, Ma L, Raita E (2012). Habits make smartphone use more pervasive. Pers Ubiquit Comput.

[54] Baryshnikov I, Aledavood T, Rosenström T (2023). Relationship between daily rated depression symptom severity and the retrospective self-report on PHQ-9: a prospective ecological momentary assessment study on 80 psychiatric outpatients. J Affect Disord.

[55] Hu Q, Li A, Heng F, Li J, Zhu T Predicting depression of social media user on different observation windows.

[56] (2023). Current population of germany. Statistisches Bundesamt (Destatis).

[57] Cohen J (1960). A coefficient of agreement for nominal scales. Educ Psychol Meas.

[58] Cohen S, Kamarck T, Mermelstein R (1983). A global measure of perceived stress. J Health Soc Behav.

[59] Philpott LF, Leahy-Warren P, FitzGerald S, Savage E (2022). Prevalence and associated factors of paternal stress, anxiety, and depression symptoms in the early postnatal period. Glob Ment Health (Camb).

[60] Biswas B, Saha R, Haldar D, Saha I (2019). Level of stress perception and predictors of higher stress perception among informal primary caregivers of Eastern Indian people living with HIV/AIDS. Int J Community Med Public Health.

[61] Bryk AS, Raudenbush SW (2002). Hierarchical Linear Models: Applications and Data Analysis Methods.

[62] Seabold S, Perktold J (2010). Statsmodels: econometric and statistical modeling with python.

[63] Thorn L, Evans P, Cannon A, Hucklebridge F, Clow A (2011). Seasonal differences in the diurnal pattern of cortisol secretion in healthy participants and those with self-assessed seasonal affective disorder. Psychoneuroendocrinology.

[64] Gassen J, Mengelkoch S, Slavich GM (2024). Human immune and metabolic biomarker levels, and stress-biomarker associations, differ by season: Implications for biomedical health research. Brain, Behavior, & Immunity - Health.

[65] (2024). Average daily time spent on internet and online media use in germany in the 3rd quarter of 2023, by device. Statista.

[66] Beyens I, Frison E, Eggermont S (2016). “I don’t want to miss a thing”: adolescents’ fear of missing out and its relationship to adolescents’ social needs, Facebook use, and Facebook related stress. Comput Human Behav.

[67] Åberg E, Koivula A, Kukkonen I (2020). A feminine burden of perfection? Appearance-related pressures on social networking sites. Telematics and Informatics.

[68] Chen W, Lee KH (2013). Sharing, liking, commenting, and distressed? The pathway between Facebook interaction and psychological distress. Cyberpsychol Behav Soc Netw.

[69] Rus HM, Tiemensma J (2018). Social media as a shield: Facebook buffers acute stress. Physiol Behav.

[70] Wolfers LN, Festl R, Utz S (2020). Do smartphones and social network sites become more important when experiencing stress? Results from longitudinal data. Comput Human Behav.

[71] Brailovskaia J, Rohmann E, Bierhoff HW, Schillack H, Margraf J (2019). The relationship between daily stress, social support and Facebook addiction disorder. Psychiatry Res.

[72] Shen X, Wang JL (2019). Loneliness and excessive smartphone use among Chinese college students: moderated mediation effect of perceived stressed and motivation. Comput Human Behav.

[73] Aghababian AH, Sadler JR, Jansen E, Thapaliya G, Smith KR, Carnell S (2021). Binge watching during COVID-19: associations with stress and body weight. Nutrients.

[74] Alimoradi Z, Jafari E, Potenza MN, Lin CY, Wu CY, Pakpour AH (2022). Binge-watching and mental health problems: a systematic review and meta-analysis. Int J Environ Res Public Health.

[75] Hunter IR, Gillen MC (2009). Stress coping mechanisms in elderly adults: an initial study of recreational and other coping behaviors in nursing home patients. Adultspan Journal.

[76] van Gelder K E-commerce worldwide - statistics & facts. Statista.

[77] Tarka P, Kukar-Kinney M (2022). Compulsive buying among young consumers in Eastern Europe: a two-study approach to scale adaptation and validation. JCM.

[78] Zheng Y, Yang X, Liu Q, Chu X, Huang Q, Zhou Z (2020). Perceived stress and online compulsive buying among women: a moderated mediation model. Comput Human Behav.

[79] Singh R, Nayak JK (2015). Life stressors and compulsive buying behaviour among adolescents in India. SAJGBR.

[80] Hama Y (2001). Shopping as a coping behavior for stress. Jpn Psychol Res.

[81] Maharani SAD, Utami NP (2023). Coping mechanisms of stress: the impact on online purchase impulsivity. JBMS.

[82] Durante KM, Laran J (2016). The effect of stress on consumer. Journal of Marketing Research.

[83] Gallagher CE, Watt MC, Weaver AD, Murphy KA (2017). “I fear, therefore, I shop!” exploring anxiety sensitivity in relation to compulsive buying. Pers Individ Dif.

[84] (2025). Games - worldwide. Statista.

[85] Lemmens JS, Valkenburg PM, Peter J (2011). Psychosocial causes and consequences of pathological gaming. Comput Human Behav.

[86] Whitbourne SK, Ellenberg S, Akimoto K (2013). Reasons for playing casual video games and perceived benefits among adults 18 to 80 years old. Cyberpsychol Behav Soc Netw.

[87] Desai V, Gupta A, Andersen L, Ronnestrand B, Wong M (2021). Stress-reducing effects of playing a casual video game among undergraduate students. Trends Psychol.

[88] Lee YH, Chen M (2023). Seeking a sense of control or escapism? The role of video games in coping with unemployment. Games and Culture.

[89] Snodgrass JG, Lacy MG, Dengah HJF, Eisenhauer S, Batchelder G, Cookson RJ (2014). A vacation from your mind: problematic online gaming is a stress response. Comput Human Behav.

[90] Seçer İ, Us EÖ (2023). Digital gaming trends of middle-aged and older adults: a sample from Turkey. Simul Gaming.

[91] Engelstätter B, Ward MR (2022). Video games become more mainstream. Entertain Comput.

[92] Aledavood T, Lehmann S, Saramäki J (2015). Digital daily cycles of individuals. Front Phys.

[93] Luong N, Barnett I, Aledavood T (2023). The impact of the COVID-19 pandemic on daily rhythms. J Am Med Inform Assoc.

[94] Schrempft S, Baysson H, Chessa A (2024). Associations between bedtime media use and sleep outcomes in an adult population-based cohort. Sleep Med.

[95] Siebers T, Beyens I, Baumgartner SE, Valkenburg PM (2024). Adolescents’ digital nightlife: the comparative effects of day- and nighttime smartphone use on sleep quality. Communic Res.

[96] Luqman A, Masood A, Shahzad F, Shahbaz M, Feng Y (2021). Untangling the adverse effects of late-night usage of smartphone-based SNS among university students. Behav Inf Technol.

[97] Thomée S, Härenstam A, Hagberg M (2011). Mobile phone use and stress, sleep disturbances, and symptoms of depression among young adults - a prospective cohort study. BMC Public Health.

[98] Thomée S, Härenstam A, Hagberg M (2012). Computer use and stress, sleep disturbances, and symptoms of depression among young adults – a prospective cohort study. BMC Psychiatry.

[99] Price GD, Heinz MV, Song SH, Nemesure MD, Jacobson NC (2023). Using digital phenotyping to capture depression symptom variability: detecting naturalistic variability in depression symptoms across one year using passively collected wearable movement and sleep data. Transl Psychiatry.

[100] Kortesoja L, Vainikainen MP, Hotulainen R, Merikanto I (2023). Late-night digital media use in relation to chronotype, sleep and tiredness on school days in adolescence. J Youth Adolesc.

[101] Bőthe B, Nagy L, Koós M (2024). Problematic pornography use across countries, genders, and sexual orientations: insights from the International Sex Survey and comparison of different assessment tools. Addiction.

[102] Laier C, Brand M (2017). Mood changes after watching pornography on the Internet are linked to tendencies towards Internet-pornography-viewing disorder. Addict Behav Rep.

[103] Altin M, De Leo D, Tribbia N, Ronconi L, Cipolletta S (2024). Problematic pornography use, mental health, and suicidality among young adults. Int J Environ Res Public Health.

[104] Harper C, Hodgins DC (2016). Examining correlates of problematic internet pornography use among university students. J Behav Addict.

[105] McCormack M, Wignall L (2017). Enjoyment, exploration and education: understanding the consumption of pornography among young men with non-exclusive sexual orientations. Sociology.

[106] Bőthe B, Tóth-Király I, Bella N, Potenza MN, Demetrovics Z, Orosz G (2021). Why do people watch pornography? The motivational basis of pornography use. Psychol Addict Behav.

[107] Mubeen B, Ashraf D (2022). Psychological predictors of adult content consumption: a qualitative analysis by grounded theory. Asian J Psychiatr.

[108] Moynihan AB, Igou ER, van Tilburg WAP (2022). Pornography consumption as existential escape from boredom. Pers Individ Dif.

[109] Federico B (2013). ICT and productivity: a review of the literature.

[110] Buhari A, A. AA (2024). Influence of internet and its connectivity in workplace - a comprehensive analysis. RRRJ.

[111] Mark G, Voida S, Cardello A A pace not dictated by electrons": an empirical study of work without email. CHI ’12: Proceedings of the SIGCHI Conference on Human Factors in Computing Systems.

[112] Ninaus K, Diehl S, Terlutter R, Chan K, Huang A, Erlandsson S (2015). Benefits and stressors - perceived effects of ICT use on employee health and work stress: an exploratory study from Austria and Hong Kong. Int J Qual Stud Health Well-being.

[113] Berg-Beckhoff G, Nielsen G, Ladekjær Larsen E (2017). Use of information communication technology and stress, burnout, and mental health in older, middle-aged, and younger workers - results from a systematic review. Int J Occup Environ Health.

[114] Malott L, Vishwanathan SP, Chellappan S Differences in internet usage patterns with stress and anxiety among college students.

[115] Allen MT (2021). Explorations of avoidance and approach coping and perceived stress with a computer-based avatar task: detrimental effects of resignation and withdrawal. PeerJ.

[116] Holahan CJ, Moos RH, Holahan CK, Brennan PL, Schutte KK (2005). Stress generation, avoidance coping, and depressive symptoms: a 10-year model. J Consult Clin Psychol.

[117] Shaffer SR, Dolovich C, El-Gabalawy R (2024). The impact of source and consumption of news on mental distress among inflammatory bowel disease patients during the COVID-19 pandemic. J Can Assoc Gastroenterol.

[118] Ladis I, Gao C, Scullin MK (2023). COVID-19-related news consumption linked with stress and worry, but not sleep quality, early in the pandemic. Psychol Health Med.

[119] Kellerman JK, Hamilton JL, Selby EA, Kleiman EM (2022). The mental health impact of daily news exposure during the COVID-19 pandemic: Ecological Momentary Assessment study. JMIR Ment Health.

[120] Nguyen A, Smith A, Jackson D, Zhao X (2021). Pandemic news experience: COVID-19, news consumption, mental health, and the demand for positive news. SSRN Journal.

[121] Lindell J, Mikkelsen Båge E (2023). Disconnecting from digital news: news avoidance and the ignored role of social class. Journalism.

[122] Mannell K, Meese J (2022). From doom-scrolling to news avoidance: limiting news as a wellbeing strategy during COVID lockdown. Journal Stud.

[123] McNaughton-cassill ME (2001). The news media and psychological distress. Anxiety, Stress & Coping.

[124] Lavelle B (2022). Investigating whether the consumption of news impacts measures for anxiety, stress, and well-being. Https://norma.ncirl.ie/id/eprint/5657.

[125] Boukes M, Vliegenthart R (2017). News consumption and its unpleasant side effect. J Media Psychol.

[126] (2021). Mobile messaging users worldwide 2025. Statista.

[127] Coccia C, Darling CA (2016). Having the time of their life: college student stress, dating and satisfaction with life. Stress Health.

[128] Thomée S, Eklöf M, Gustafsson E, Nilsson R, Hagberg M (2007). Prevalence of perceived stress, symptoms of depression and sleep disturbances in relation to information and communication technology (ICT) use among young adults – an explorative prospective study. Comput Human Behav.

[129] Hurbean L, Dospinescu O, Munteanu V, Danaiata D (2022). Effects of instant messaging related technostress on work performance and well-being. Electronics (Basel).

[130] Lin XY, Lachman ME (2021). Daily stress and affect across adulthood: the role of social interactions via different communication modes. Technol Mind Behav.

[131] Melumad S, Pham MT (2020). The smartphone as a pacifying technology. J Consum Res.

[132] Yau JC, Reich SM, Lee TY (2021). Coping with stress through texting: an experimental study. J Adolesc Health.

[133] Holtzman S, DeClerck D, Turcotte K, Lisi D, Woodworth M (2017). Emotional support during times of stress: Can text messaging compete with in-person interactions?. Comput Human Behav.

[134] Hooker ED, Campos B, Pressman SD (2018). It just takes a text: partner text messages can reduce cardiovascular responses to stress in females. Comput Human Behav.

[135] Smith A How Americans use text messaging. Pew Research Center.

[136] Aguilera A, Muñoz RF (2011). Text messaging as an adjunct to CBT in low-income populations: a usability and feasibility pilot study. Prof Psychol Res Pr.

[137] George MJ, Beron K, Vollet JW, Burnell K, Ehrenreich SE, Underwood MK (2021). Frequency of text messaging and adolescents’ mental health symptoms across 4 years of high school. J Adolesc Health.

[138] Yu CC, Tou NX, Low JA (2024). Internet use and effects on mental well-being during the lockdown phase of the COVID-19 pandemic in younger versus older adults: observational cross-sectional study. JMIR Form Res.

[139] Elamin NO, Almasaad JM, Busaeed RB, Aljafari DA, Khan MA (2024). Smartphone addiction, stress, and depression among university students. Clin Epidemiol Glob Health.

[140] Khan A, McLeod G, Hidajat T, Edwards EJ (2023). Excessive smartphone use is associated with depression, anxiety, stress, and sleep quality of Australian adults. J Med Syst.

[141] Ding L, Li Z, Jiang H, Zhang X, Xiong Z, Zhu X (2024). Mobile phone problem use and depressive symptoms: the mediating role of social support and attitude to aging among Chinese older adults. BMC Psychiatry.

[142] Yue C, Ware S, Morillo R (2020). Automatic depression prediction using Internet traffic characteristics on smartphones. Smart Health (2014).

[143] Bartone PT, Ursano RJ, Wright KM, Ingraham LH (1989). The impact of a military air disaster on the health of assistance workers. A prospective study. J Nerv Ment Dis.

[144] Kulshrestha J, Oliveira M, Karaçalık O, Bonnay D, Wagner C (2021). Web routineness and limits of predictability: investigating demographic and behavioral differences using web tracking data. ICWSM.

[145] Stier S, Kirkizh N, Froio C, Schroeder R (2020). Populist attitudes and selective exposure to online news: a cross-country analysis combining web tracking and surveys. Int J Press Polit.

[146] Grothaus C, Dolch C, Zawacki-Richter O (2021). Use of digital media in higher education across country contexts: a comparison between Germany and Thailand. Int J Emerg Technol Learn.

[147] Lopez-Fernandez O, Romo L, Kern L (2023). Problematic internet use among adults: a cross-cultural study in 15 countries. J Clin Med.

