# Supplementary material for: Examining the Association Between Internet Use and Perceived Stress in Adults: Longitudinal Observational Study Combining Web Tracking Data With Questionnaires
Source: J Med Internet Res. 2026 Jan 9;28:e78775. doi: 10.2196/78775 (PMC12792356; doi:10.2196/78775)
Supplement: Multimedia Appendix 1 [file jmir-v28-e78775-s001.docx]

**Multimedia Appendix 1.** Mixed-effect models results across contextual dimensions.

The following subsections present the results of both models across different groups and contexts. Model 1 includes coarse-grained features, while Model 2 incorporates fine-grained usage categories. For each predictor, we report estimates, confidence intervals (CI), and p-values, with statistically significant p-values bolded. Additionally, random effects, intraclass correlation coefficient (ICC), number of participants (N), and total observations are provided.

**Section 1.** Time period of data (When).

**Table S1:** Results from mixed-effects models for All Participants, based on 2 days of mobile usage data.

| **Predictors** | Model 1 | | | Model 2 | | |
| --- | --- | --- | --- | --- | --- | --- |
|  | Estimate | CI | p | Estimate | CI | p |
| Intercept | 20.67*** | 18.89–22.44 | **<0.001** | 20.57*** | 18.79–22.35 | **<0.001** |
| Survey wave | -0.11* | -0.21– -0.01 | **0.024** | -0.11* | -0.20– -0.01 | **0.030** |
| Gender | 2.02*** | 0.97–3.06 | **<0.001** | 1.95*** | 0.90–2.99 | **<0.001** |
| Age | -1.60*** | -2.22– -0.98 | **<0.001** | -1.56*** | -2.18– -0.93 | **<0.001** |
| Income | -1.17*** | -1.58– -0.76 | **<0.001** | -1.14*** | -1.56– -0.73 | **<0.001** |
| Total time spent online | 0.00 | -0.04–0.05 | 0.855 |  |  |  |
| Daytime nighttime difference | 0.05 | -0.03–0.12 | 0.203 | 0.05 | -0.03–0.12 | 0.223 |
| Time spent on entertainment |  |  |  | 0.01 | -0.12–0.15 | 0.840 |
| Time spent on social media |  |  |  | 0.02 | -0.11–0.15 | 0.811 |
| Time spent on messaging |  |  |  | 0.11 | -0.05–0.28 | 0.171 |
| Time spent on games |  |  |  | 0.01 | -0.08–0.10 | 0.780 |
| Time spent on shopping |  |  |  | -0.04 | -0.35–0.28 | 0.816 |
| Time spent on productivity |  |  |  | -0.05 | -0.29–0.18 | 0.648 |
| Time spent on the news |  |  |  | -0.54* | -1.08– -0.00 | **0.048** |
| **Random Effects** | | | | | | |
| σ^2^ | 12.02 | | | 12.02 | | |
| τ_00_ | 39.49_pid_ | | | 39.43_pid_ | | |
| ICC | 0.77 | | | 0.77 | | |
| N | 640 _pid_ | | | 640_pid_ | | |
| Observations | 2141 | | | 2141 | | |
| **** p<0.05 ** p<0.01 *** p<0.001*** | | | | | | |

**Table S2.** Results from mixed-effects models for All Participants, based on 2 days of desktop usage data.

| **Predictors** | Model 1 | | | Model 2 | | |
| --- | --- | --- | --- | --- | --- | --- |
|  | Estimate | CI | p | Estimate | CI | p |
| Intercept | 21.26*** | 19.19–23.32 | **<0.001** | 21.23*** | 19.17–23.29 | **<0.001** |
| Survey wave | -0.14* | -0.27– -0.01 | **0.036** | -0.12 | -0.25–0.01 | 0.074 |
| Gender | 1.53* | 0.33–2.74 | **0.013** | 1.47* | 0.26–2.68 | **0.017** |
| Age | -1.96*** | -2.68– -1.24 | **<0.001** | -1.92*** | -2.65– -1.19 | **<0.001** |
| Income | -1.04*** | -1.50– -0.58 | **<0.001** | -1.02*** | -1.48– -0.56 | **<0.001** |
| Total time spent online | -0.01 | -0.08–0.05 | 0.695 |  |  |  |
| Daytime nighttime difference | 0.05 | -0.04–0.14 | 0.288 | 0.05 | -0.04–0.14 | 0.244 |
| Time spent on entertainment |  |  |  | 0.05 | -0.05–0.15 | 0.356 |
| Time spent on adult content |  |  |  | -0.12 | -0.32–0.09 | 0.253 |
| Time spent on social media |  |  |  | 0.07 | -0.16–0.29 | 0.563 |
| Time spent on messaging |  |  |  | -0.59 | -1.24–0.06 | 0.073 |
| Time spent on games |  |  |  | -0.23 | -0.56–0.09 | 0.160 |
| Time spent on shopping |  |  |  | -0.10 | -0.41–0.20 | 0.507 |
| Time spent on productivity |  |  |  | 0.03 | -0.22–0.28 | 0.799 |
| Time spent on news |  |  |  | -0.50* | -0.90– -0.11 | **0.012** |
| **Random Effects** | | | | | | |
| σ^2^ | 10.75 | | | 10.63 | | |
| τ_00_ | 40.38_pid_ | | | 40.52 _pid_ | | |
| ICC | 0.79 | | | 0.79 | | |
| N | 501 _pid_ | | | 501 _pid_ | | |
| Observations | 1207 | | | 1207 | | |
| **** p<0.05 ** p<0.01 *** p<0.001*** | | | | | | |

**Section 2.** Stress levels.

**Table S3.** Results from mixed-effects models for High Stress, based on 30 days of mobile.

| **Predictors** | Model 1 | | | Model 2 | | |
| --- | --- | --- | --- | --- | --- | --- |
|  | Estimate | CI | p | Estimate | CI | p |
| Intercept | 29.70*** | 27.97–31.43 | **<0.001** | 29.75*** | 27.97–31.52 | **<0.001** |
| Survey wave | 0.09 | -0.08–0.27 | 0.305 | 0.10 | -0.09–0.28 | 0.298 |
| Gender | -0.42 | -1.32–0.48 | 0.357 | -0.30 | -1.23–0.64 | 0.533 |
| Age | -0.35 | -0.90–0.20 | 0.212 | -0.36 | -0.92–0.21 | 0.217 |
| Income | -0.50** | -0.88– -0.12 | **0.010** | -0.52* | -0.91– -0.12 | **0.010** |
| Total time spent online | 0.01*** | 0.00–0.02 | **<0.001** |  |  |  |
| Daytime nighttime difference | 0.01 | -0.01–0.02 | 0.241 | 0.01 | -0.01–0.02 | 0.424 |
| Time spent on entertainment |  |  |  | 0.00 | -0.02–0.02 | 0.826 |
| Time spent on social media |  |  |  | 0.02* | 0.00–0.03 | **0.021** |
| Time spent on messaging |  |  |  | 0.00 | -0.03–0.03 | 0.846 |
| Time spent on games |  |  |  | 0.01* | 0.00–0.02 | **0.021** |
| Time spent on shopping |  |  |  | -0.01 | -0.07–0.05 | 0.697 |
| Time spent on productivity |  |  |  | 0.02 | -0.02–0.07 | 0.339 |
| Time spent on news |  |  |  | 0.09 | -0.02–0.20 | 0.102 |
| **Random Effects** | | | | | | |
| σ^2^ | 3.49 | | | 3.55 | | |
| τ_00_ | 2.33_pid_ | | | 2.27 _pid_ | | |
| ICC | 0.40 | | | 0.39 | | |
| N | 94 _pid_ | | | 94 _pid_ | | |
| Observations | 197 | | | 197 | | |
| **** p<0.05 ** p<0.01 *** p<0.001*** | | | | | | |

**Table S4.** Results from mixed-effects models for High Stress, based on 30 days of desktop usage data.

| **Predictors** | Model 1 | | | Model 2 | | |
| --- | --- | --- | --- | --- | --- | --- |
|  | Estimate | CI | p | Estimate | CI | p |
| Intercept | 32.53*** | 30.04–35.01 | **<0.001** | 32.48*** | 29.89–35.07 | **<0.001** |
| Survey wave | 0.04 | -0.21–0.30 | 0.756 | 0.05 | -0.21–0.32 | 0.703 |
| Gender | -0.72 | -2.11–0.67 | 0.309 | -0.99 | -2.49–0.52 | 0.199 |
| Age | -0.80 | -1.67–0.08 | 0.074 | -0.68 | -1.62–0.26 | 0.156 |
| Income | -0.43 | -0.99–0.12 | 0.127 | -0.37 | -0.95–0.21 | 0.211 |
| Total time spent online | -0.00 | -0.01–0.01 | 0.593 |  |  |  |
| Daytime nighttime difference | -0.01 | -0.03–0.01 | 0.453 | -0.00 | -0.02–0.02 | 0.946 |
| Time spent on entertainment |  |  |  | 0.02 | -0.01–0.05 | 0.205 |
| Time spent on adult content |  |  |  | -0.03 | -0.13–0.08 | 0.593 |
| Time spent on social media |  |  |  | 0.00 | -0.02–0.02 | 0.975 |
| Time spent on messaging |  |  |  | 0.01 | -0.27–0.29 | 0.931 |
| Time spent on games |  |  |  | 0.00 | -0.03–0.04 | 0.839 |
| Time spent on shopping |  |  |  | -0.01 | -0.06–0.05 | 0.785 |
| Time spent on productivity |  |  |  | -0.04 | -0.09–0.01 | 0.133 |
| Time spent on the news |  |  |  | -0.00 | -0.11–0.10 | 0.959 |
| **Random Effects** | | | | | | |
| σ^2^ | 4.05 | | | 4.20 | | |
| τ_00_ | 4.82_pid_ | | | 4.96 _pid_ | | |
| ICC | 0.54 | | | 0.54 | | |
| N | 63 _pid_ | | | 63 _pid_ | | |
| Observations | 132 | | | 132 | | |
| **** p<0.05 ** p<0.01 *** p<0.001*** | | | | | | |

**Table S5**. Results from mixed-effects models for High Stress, based on 2 days of mobile usage data.

| **Predictors** | Model 1 | | | Model 2 | | |
| --- | --- | --- | --- | --- | --- | --- |
|  | Estimate | CI | p | Estimate | CI | p |
| Intercept | 31.83*** | 30.02–33.63 | **<0.001** | 31.68*** | 29.88–33.48 | **<0.001** |
| Survey wave | -0.03 | -0.22–0.17 | 0.799 | -0.02 | -0.22–0.18 | 0.868 |
| Gender | -0.73 | -1.75–0.30 | 0.166 | -0.66 | -1.70–0.38 | 0.215 |
| Age | -0.27 | -0.88–0.34 | 0.389 | -0.28 | -0.90–0.34 | 0.373 |
| Income | -0.67** | -1.11– -0.24 | **0.002** | -0.64** | -1.07– -0.21 | **0.004** |
| Total time spent online | 0.01 | -0.06–0.08 | 0.797 |  |  |  |
| Daytime nighttime difference | 0.11 | -0.01–0.23 | 0.075 | 0.09 | -0.04–0.22 | 0.154 |
| Time spent on entertainment |  |  |  | -0.04 | -0.24–0.17 | 0.730 |
| Time spent on social media |  |  |  | 0.04 | -0.15–0.23 | 0.686 |
| Time spent on messaging |  |  |  | -0.03 | -0.21–0.16 | 0.784 |
| Time spent on games |  |  |  | 0.06 | -0.09–0.20 | 0.447 |
| Time spent on shopping |  |  |  | 0.01 | -0.59–0.61 | 0.972 |
| Time spent on productivity |  |  |  | -0.14 | -0.63–0.35 | 0.577 |
| Time spent on news |  |  |  | 0.83 | -0.86–2.51 | 0.338 |
| **Random Effects** | | | | | | |
| σ^2^ | 2.92 | | | 3.11 | | |
| τ_00_ | 3.92_pid_ | | | 3.68 _pid_ | | |
| ICC | 0.57 | | | 0.54 | | |
| N | 91 _pid_ | | | 91 _pid_ | | |
| Observations | 171 | | | 171 | | |
| **** p<0.05 ** p<0.01 *** p<0.001*** | | | | | | |

**Table S6.** Results from mixed-effects models for High Stress, based on 2 days of desktop usage data.

| **Predictors** | Model 1 | | | Model 2 | | |
| --- | --- | --- | --- | --- | --- | --- |
|  | Estimate | CI | p | Estimate | CI | p |
| Intercept | 33.56*** | 30.68–36.44 | **<0.001** | 33.81*** | 30.76–36.85 | **<0.001** |
| Survey wave | -0.05 | -0.32–0.23 | 0.749 | -0.07 | -0.38–0.23 | 0.634 |
| Gender | -1.29 | -2.84–0.27 | 0.105 | -1.47 | -3.20–0.26 | 0.096 |
| Age | -1.04* | -2.04– -0.03 | **0.043** | -1.02 | -2.06–0.03 | 0.057 |
| Income | -0.26 | -0.89–0.36 | 0.407 | -0.31 | -0.99–0.36 | 0.365 |
| Total time spent online | -0.06 | -0.17–0.05 | 0.278 |  |  |  |
| Daytime nighttime difference | 0.13 | -0.05–0.32 | 0.155 | 0.15 | -0.05–0.36 | 0.145 |
| Time spent on entertainment |  |  |  | -0.04 | -0.21–0.14 | 0.692 |
| Time spent on adult content |  |  |  | -0.73 | -1.81–0.35 | 0.183 |
| Time spent on social media |  |  |  | -0.02 | -0.33–0.29 | 0.898 |
| Time spent on messaging |  |  |  | 1.02 | -1.74–3.78 | 0.468 |
| Time spent on games |  |  |  | -0.00 | -0.43–0.43 | 0.996 |
| Time spent on shopping |  |  |  | -0.33 | -0.89–0.23 | 0.254 |
| Time spent on productivity |  |  |  | -0.12 | -0.65–0.40 | 0.648 |
| Time spent on news |  |  |  | 0.16 | -0.81–1.14 | 0.741 |
| **Random Effects** | | | | | | |
| σ^2^ | 4.49 | | | 4.71 | | |
| τ_00_ | 5.17_pid_ | | | 5.33 _pid_ | | |
| ICC | 0.54 | | | 0.53 | | |
| N | 55 _pid_ | | | 55 _pid_ | | |
| Observations | 106 | | | 106 | | |
| **** p<0.05 ** p<0.01 *** p<0.001*** | | | | | | |

**Table S7.** Results from mixed-effects models for Low Stress, based on 30 days of mobile usage data.

| **Predictors** | Model 1 | | | Model 2 | | |
| --- | --- | --- | --- | --- | --- | --- |
|  | Estimate | CI | p | Estimate | CI | p |
| Intercept | 10.63*** | 9.39–11.88 | **<0.001** | 10.39*** | 9.13–11.65 | **<0.001** |
| Survey wave | -0.20*** | -0.30– -0.11 | **<0.001** | -0.20*** | -0.29– -0.10 | **<0.001** |
| Gender | 1.19*** | 0.55–1.83 | **<0.001** | 1.08** | 0.42–1.74 | **0.001** |
| Age | -0.55** | -0.94– -0.17 | **0.005** | -0.54** | -0.93– -0.15 | **0.007** |
| Income | -0.12 | -0.37–0.13 | 0.345 | -0.11 | -0.36–0.15 | 0.414 |
| Total time spent online | -0.00 | -0.01–0.00 | 0.335 |  |  |  |
| Daytime nighttime difference | 0.00 | -0.01–0.01 | 0.737 | -0.00 | -0.01–0.01 | 0.923 |
| Time spent on entertainment |  |  |  | -0.01 | -0.03–0.00 | 0.124 |
| Time spent on social media |  |  |  | 0.01 | -0.01–0.02 | 0.223 |
| Time spent on messaging |  |  |  | 0.00 | -0.02–0.02 | 0.808 |
| Time spent on games |  |  |  | -0.00 | -0.01–0.01 | 0.995 |
| Time spent on shopping |  |  |  | 0.02 | -0.02–0.06 | 0.288 |
| Time spent on productivity |  |  |  | -0.02 | -0.05–0.01 | 0.141 |
| Time spent on the news |  |  |  | 0.00 | -0.04–0.05 | 0.866 |
| **Random Effects** | | | | | | |
| σ^2^ | 5.66 | | | 5.65 | | |
| τ_00_ | 6.12_pid_ | | | 6.14 _pid_ | | |
| ICC | 0.52 | | | 0.52 | | |
| N | 334 _pid_ | | | 334 _pid_ | | |
| Observations | 1011 | | | 1011 | | |
| **** p<0.05 ** p<0.01 *** p<0.001*** | | | | | | |

**Table S8.** Results from mixed-effects models for Low Stress, based on 30 days of desktop usage data.

| **Predictors** | Model 1 | | | Model 2 | | |
| --- | --- | --- | --- | --- | --- | --- |
|  | Estimate | CI | p | Estimate | CI | p |
| Intercept | 10.27*** | 8.80–11.75 | **<0.001** | 10.25*** | 8.77–11.73 | **<0.001** |
| Survey wave | -0.14** | -0.25– -0.04 | **0.009** | -0.15** | -0.26– -0.04 | **0.008** |
| Gender | 0.37 | -0.41–1.15 | 0.355 | 0.36 | -0.43–1.15 | 0.376 |
| Age | -0.56* | -1.05– -0.07 | **0.024** | -0.57* | -1.07– -0.07 | **0.026** |
| Income | 0.02 | -0.28–0.33 | 0.882 | 0.01 | -0.30–0.33 | 0.935 |
| Total time spent online | -0.00 | -0.01–0.00 | 0.119 |  |  |  |
| Daytime nighttime difference | 0.00 | -0.00–0.01 | 0.263 | 0.01 | -0.00–0.01 | 0.232 |
| Time spent on entertainment |  |  |  | -0.00 | -0.01–0.00 | 0.341 |
| Time spent on adult content |  |  |  | -0.02 | -0.04–0.00 | 0.065 |
| Time spent on social media |  |  |  | -0.01 | -0.02–0.01 | 0.409 |
| Time spent on messaging |  |  |  | 0.02 | -0.01–0.05 | 0.276 |
| Time spent on games |  |  |  | -0.01 | -0.04–0.01 | 0.414 |
| Time spent on shopping |  |  |  | -0.00 | -0.04–0.03 | 0.936 |
| Time spent on productivity |  |  |  | 0.00 | -0.02–0.02 | 0.951 |
| Time spent on news |  |  |  | -0.00 | -0.03–0.03 | 0.861 |
| **Random Effects** | | | | | | |
| σ^2^ | 4.93 | | | 4.93 | | |
| τ_00_ | 7.31_pid_ | | | 7.42 _pid_ | | |
| ICC | 0.60 | | | 0.60 | | |
| N | 268 _pid_ | | | 268 _pid_ | | |
| Observations | 712 | | | 712 | | |
| **** p<0.05 ** p<0.01 *** p<0.001*** | | | | | | |

**Table S9.** Results from mixed-effects models for Low Stress, based on 2 days of mobile usage data.

| **Predictors** | Model 1 | | | Model 2 | | |
| --- | --- | --- | --- | --- | --- | --- |
|  | Estimate | CI | p | Estimate | CI | p |
| Intercept | 10.25*** | 8.95–11.55 | **<0.001** | 10.30*** | 8.98–11.62 | **<0.001** |
| Survey wave | -0.24*** | -0.34– -0.13 | **<0.001** | -0.23*** | -0.34– -0.12 | **<0.001** |
| Gender | 1.18*** | 0.51–1.84 | **<0.001** | 1.10** | 0.43–1.78 | **0.001** |
| Age | -0.52* | -0.93– -0.12 | **0.011** | -0.55** | -0.97– -0.14 | **0.008** |
| Income | -0.12 | -0.38–0.15 | 0.379 | -0.10 | -0.37–0.17 | 0.460 |
| Total time spent online | 0.01 | -0.05–0.06 | 0.813 |  |  |  |
| Daytime nighttime difference | 0.00 | -0.08–0.09 | 0.971 | -0.02 | -0.11–0.06 | 0.606 |
| Time spent on entertainment |  |  |  | -0.13 | -0.29–0.04 | 0.136 |
| Time spent on social media |  |  |  | -0.03 | -0.20–0.13 | 0.684 |
| Time spent on messaging |  |  |  | 0.09 | -0.14–0.32 | 0.453 |
| Time spent on games |  |  |  | 0.08 | -0.01–0.17 | 0.098 |
| Time spent on shopping |  |  |  | 0.04 | -0.28–0.37 | 0.799 |
| Time spent on productivity |  |  |  | 0.04 | -0.15–0.23 | 0.696 |
| Time spent on news |  |  |  | -0.30 | -0.81–0.22 | 0.257 |
| **Random Effects** | | | | | | |
| σ^2^ | 5.96 | | | 5.92 | | |
| τ_00_ | 6.03_pid_ | | | 6.13 _pid_ | | |
| ICC | 0.50 | | | 0.51 | | |
| N | 323 _pid_ | | | 323 _pid_ | | |
| Observations | 849 | | | 849 | | |
| **** p<0.05 ** p<0.01 *** p<0.001*** | | | | | | |

**Table S10**. Results from mixed-effects models for Low Stress, based on 2 days of desktop usage data.

| **Predictors** | Model 1 | | | Model 2 | | |
| --- | --- | --- | --- | --- | --- | --- |
|  | Estimate | CI | p | Estimate | CI | p |
| Intercept | 10.04*** | 8.32–11.77 | **<0.001** | 10.01*** | 8.28–11.75 | **<0.001** |
| Survey wave | -0.20** | -0.33– -0.07 | **0.003** | -0.21** | -0.35– -0.07 | **0.003** |
| Gender | 0.35 | -0.48–1.18 | 0.411 | 0.35 | -0.49–1.19 | 0.413 |
| Age | -0.10 | -0.66–0.46 | 0.720 | -0.10 | -0.67–0.47 | 0.729 |
| Income | -0.21 | -0.54–0.12 | 0.211 | -0.22 | -0.56–0.11 | 0.193 |
| Total time spent online | -0.07* | -0.14– -0.00 | **0.038** |  |  |  |
| Daytime nighttime difference | 0.04 | -0.05–0.12 | 0.367 | 0.04 | -0.05–0.13 | 0.438 |
| Time spent on entertainment |  |  |  | -0.04 | -0.16–0.08 | 0.516 |
| Time spent on adult content |  |  |  | -0.11 | -0.31–0.10 | 0.310 |
| Time spent on social media |  |  |  | -0.10 | -0.31–0.10 | 0.316 |
| Time spent on messaging |  |  |  | -0.21 | -0.56–0.15 | 0.253 |
| Time spent on games |  |  |  | -0.24 | -0.57–0.09 | 0.155 |
| Time spent on shopping |  |  |  | -0.13 | -0.44–0.18 | 0.408 |
| Time spent on productivity |  |  |  | 0.07 | -0.14–0.28 | 0.504 |
| Time spent on news |  |  |  | -0.16 | -0.52–0.20 | 0.387 |
| **Random Effects** | | | | | | |
| σ^2^ | 4.86 | | | 4.88 | | |
| τ_00_ | 7.31_pid_ | | | 7.43 _pid_ | | |
| ICC | 0.60 | | | 0.60 | | |
| N | 241 _pid_ | | | 241 _pid_ | | |
| Observations | 500 | | | 500 | | |
| **** p<0.05 ** p<0.01 *** p<0.001*** | | | | | | |

**Section 3.** Gender differences.

**Table S11.** Results from mixed-effects models for Male, based on 30 days of mobile usage data.

| **Predictors** | Model 1 | | | Model 2 | | |
| --- | --- | --- | --- | --- | --- | --- |
|  | Estimate | CI | p | Estimate | CI | p |
| Intercept | 21.43*** | 19.22–23.65 | **<0.001** | 21.14*** | 18.92–23.35 | **<0.001** |
| Survey wave | -0.12* | -0.24– -0.01 | **0.035** | -0.13* | -0.25– -0.01 | **0.028** |
| Age | -1.81*** | -2.65– -0.97 | **<0.001** | -1.74*** | -2.59– -0.89 | **<0.001** |
| Income | -1.19*** | -1.74– -0.64 | **<0.001** | -1.12*** | -1.67– -0.57 | **<0.001** |
| Total time spent online | 0.00 | -0.01–0.01 | 0.803 |  |  |  |
| Daytime nighttime difference | -0.01 | -0.02–0.01 | 0.365 | -0.01 | -0.02–0.01 | 0.462 |
| Time spent on entertainment |  |  |  | -0.00 | -0.02–0.02 | 0.901 |
| Time spent on social media |  |  |  | 0.01 | -0.01–0.03 | 0.416 |
| Time spent on messaging |  |  |  | 0.01 | -0.02–0.03 | 0.615 |
| Time spent on games |  |  |  | 0.00 | -0.01–0.02 | 0.602 |
| Time spent on shopping |  |  |  | 0.07* | 0.01–0.14 | **0.022** |
| Time spent on productivity |  |  |  | -0.05* | -0.09– -0.01 | **0.021** |
| Time spent on news |  |  |  | -0.04 | -0.11–0.03 | 0.241 |
| **Random Effects** | | | | | | |
| σ^2^ | 11.93 | | | 11.89 | | |
| τ_00_ | 37.51_pid_ | | | 37.17 _pid_ | | |
| ICC | 0.76 | | | 0.76 | | |
| N | 334 _pid_ | | | 334 _pid_ | | |
| Observations | 1334 | | | 1334 | | |
| **** p<0.05 ** p<0.01 *** p<0.001*** | | | | | | |

**Table S12**. Results from mixed-effects models for Female, based on 30 days of mobile usage data.

| **Predictors** | Model 1 | | | Model 2 | | |
| --- | --- | --- | --- | --- | --- | --- |
|  | Estimate | CI | p | Estimate | CI | p |
| Intercept | 21.80*** | 19.58–24.02 | **<0.001** | 21.94*** | 19.68–24.19 | **<0.001** |
| Survey wave | -0.08 | -0.20–0.04 | 0.192 | -0.08 | -0.20–0.04 | 0.171 |
| Age | -1.41*** | -2.21– -0.62 | **<0.001** | -1.44*** | -2.25– -0.62 | **<0.001** |
| Income | -1.00*** | -1.54– -0.46 | **<0.001** | -1.01*** | -1.56– -0.45 | **<0.001** |
| Total time spent online | 0.00 | -0.00–0.01 | 0.324 |  |  |  |
| Daytime nighttime difference | -0.01 | -0.02–0.00 | 0.214 | -0.01 | -0.02–0.01 | 0.279 |
| Time spent on entertainment |  |  |  | 0.02 | -0.01–0.04 | 0.140 |
| Time spent on social media |  |  |  | -0.00 | -0.02–0.01 | 0.711 |
| Time spent on messaging |  |  |  | -0.00 | -0.03–0.02 | 0.889 |
| Time spent on games |  |  |  | 0.00 | -0.01–0.02 | 0.389 |
| Time spent on shopping |  |  |  | 0.02 | -0.03–0.07 | 0.466 |
| Time spent on productivity |  |  |  | -0.01 | -0.05–0.03 | 0.610 |
| Time spent on news |  |  |  | 0.03 | -0.10–0.16 | 0.676 |
| **Random Effects** | | | | | | |
| σ^2^ | 11.41 | | | 11.41 | | |
| τ_00_ | 35.76 _pid_ | | | 36.19 _pid_ | | |
| ICC | 0.76 | | | 0.76 | | |
| N | 322_pid_ | | | 322 _pid_ | | |
| Observations | 1266 | | | 1266 | | |
| **** p<0.05 ** p<0.01 *** p<0.001*** | | | | | | |

**Table S13**. Results from mixed-effects models for Male, based on 30 days of desktop usage data.

| **Predictors** | Model 1 | | | Model 2 | | |
| --- | --- | --- | --- | --- | --- | --- |
|  | Estimate | CI | p | Estimate | CI | p |
| Intercept | 20.38*** | 17.91–22.85 | **<0.001** | 20.16*** | 17.70–22.62 | **<0.001** |
| Survey wave | -0.15* | -0.29– -0.02 | **0.029** | -0.17* | -0.31– -0.02 | **0.021** |
| Age | -1.52** | -2.47– -0.57 | **0.002** | -1.50** | -2.47– -0.54 | **0.002** |
| Income | -0.93** | -1.50– -0.35 | **0.002** | -0.92** | -1.51– -0.34 | **0.002** |
| Total time spent online | -0.00 | -0.01–0.00 | 0.359 |  |  |  |
| Daytime nighttime difference | -0.00 | -0.01–0.01 | 0.797 | -0.00 | -0.01–0.01 | 0.910 |
| Time spent on entertainment |  |  |  | -0.00 | -0.01–0.01 | 0.973 |
| Time spent on adult content |  |  |  | -0.01 | -0.03–0.01 | 0.230 |
| Time spent on social media |  |  |  | 0.01 | -0.02–0.04 | 0.677 |
| Time spent on messaging |  |  |  | 0.03 | -0.06–0.12 | 0.524 |
| Time spent on games |  |  |  | -0.03 | -0.07–0.02 | 0.276 |
| Time spent on shopping |  |  |  | 0.02 | -0.02–0.07 | 0.334 |
| Time spent on productivity |  |  |  | -0.00 | -0.03–0.03 | 0.762 |
| Time spent on news |  |  |  | -0.01 | -0.06–0.03 | 0.595 |
| **Random Effects** | | | | | | |
| σ^2^ | 11.23 | | | 11.27 | | |
| τ_00_ | 39.79_pid_ | | | 39.96 _pid_ | | |
| ICC | 0.78 | | | 0.78 | | |
| N | 289 _pid_ | | | 289 _pid_ | | |
| Observations | 974 | | | 974 | | |
| **** p<0.05 ** p<0.01 *** p<0.001*** | | | | | | |

**Table S14**. Results from mixed-effects models for Female, based on 30 days of desktop usage data.

| **Predictors** | Model 1 | | | Model 2 | | |
| --- | --- | --- | --- | --- | --- | --- |
|  | Estimate | CI | p | Estimate | CI | p |
| Intercept | 22.76*** | 20.10–25.43 | **<0.001** | 22.68*** | 20.01–25.36 | **<0.001** |
| Survey wave | -0.11 | -0.28–0.05 | 0.162 | -0.12 | -0.28–0.04 | 0.146 |
| Age | -1.97*** | -2.96– -0.98 | **<0.001** | -1.99*** | -3.00– -0.98 | **<0.001** |
| Income | -0.98** | -1.65– -0.31 | **0.004** | -0.98** | -1.66– -0.30 | **0.005** |
| Total time spent online | -0.00 | -0.01–0.01 | 0.692 |  |  |  |
| Daytime nighttime difference | 0.01 | -0.00–0.03 | 0.152 | 0.01 | -0.00–0.03 | 0.105 |
| Time spent on entertainment |  |  |  | -0.01 | -0.04–0.01 | 0.226 |
| Time spent on adult content |  |  |  | -0.02 | -0.05–0.01 | 0.226 |
| Time spent on social media |  |  |  | -0.00 | -0.03–0.02 | 0.699 |
| Time spent on messaging |  |  |  | 0.00 | -0.06–0.06 | 0.973 |
| Time spent on games |  |  |  | 0.03 | -0.02–0.07 | 0.233 |
| Time spent on shopping |  |  |  | 0.04 | -0.01–0.09 | 0.147 |
| Time spent on productivity |  |  |  | -0.00 | -0.05–0.05 | 0.978 |
| Time spent on news |  |  |  | -0.05 | -0.12–0.02 | 0.146 |
| **Random Effects** | | | | | | |
| σ^2^ | 10.95 | | | 10.89 | | |
| τ_00_ | 41.82_pid_ | | | 42.19 _pid_ | | |
| ICC | 0.79 | | | 0.79 | | |
| N | 237 _pid_ | | | 237 _pid_ | | |
| Observations | 739 | | | 739 | | |
| **** p<0.05 ** p<0.01 *** p<0.001*** | | | | | | |

**Table S15**. Results from mixed-effects models for Male, based on 2 days of mobile usage data.

| **Predictors** | Model 1 | | | Model 2 | | |
| --- | --- | --- | --- | --- | --- | --- |
|  | Estimate | CI | p | Estimate | CI | p |
| Intercept | 21.94*** | 19.55–24.32 | **<0.001** | 21.88*** | 19.50–24.27 | **<0.001** |
| Survey wave | -0.11 | -0.24–0.03 | 0.120 | -0.11 | -0.25–0.03 | 0.112 |
| Age | -2.12*** | -3.01– -1.23 | **<0.001** | -2.08*** | -2.97– -1.18 | **<0.001** |
| Income | -1.26*** | -1.85– -0.68 | **<0.001** | -1.23*** | -1.81– -0.64 | **<0.001** |
| Total time spent online | -0.02 | -0.09–0.05 | 0.576 |  |  |  |
| Daytime nighttime difference | 0.01 | -0.09–0.11 | 0.850 | 0.01 | -0.09–0.12 | 0.847 |
| Time spent on entertainment |  |  |  | -0.08 | -0.27–0.11 | 0.414 |
| Time spent on social media |  |  |  | 0.07 | -0.12–0.27 | 0.471 |
| Time spent on messaging |  |  |  | 0.03 | -0.19–0.25 | 0.789 |
| Time spent on games |  |  |  | 0.01 | -0.13–0.15 | 0.891 |
| Time spent on shopping |  |  |  | -0.23 | -0.74–0.27 | 0.365 |
| Time spent on productivity |  |  |  | -0.07 | -0.35–0.22 | 0.637 |
| Time spent on news |  |  |  | -0.58 | -1.23–0.07 | 0.079 |
| **Random Effects** | | | | | | |
| σ^2^ | 12.59 | | | 12.61 | | |
| τ_00_ | 41.22_pid_ | | | 41.08 _pid_ | | |
| ICC | 0.77 | | | 0.77 | | |
| N | 335 _pid_ | | | 335 _pid_ | | |
| Observations | 1092 | | | 1092 | | |
| **** p<0.05 ** p<0.01 *** p<0.001*** | | | | | | |

**Table S16**. Results from mixed-effects models for Female, based on 2 days of mobile usage data.

| **Predictors** | Model 1 | | | Model 2 | | |
| --- | --- | --- | --- | --- | --- | --- |
|  | Estimate | CI | p | Estimate | CI | p |
| Intercept | 21.31*** | 19.02–23.59 | **<0.001** | 21.19*** | 18.89–23.49 | **<0.001** |
| Survey wave | -0.11 | -0.24–0.03 | 0.124 | -0.10 | -0.24–0.03 | 0.136 |
| Age | -1.08* | -1.93– -0.22 | **0.014** | -1.07* | -1.93– -0.20 | **0.016** |
| Income | -1.03*** | -1.60– -0.45 | **<0.001** | -1.02*** | -1.60– -0.44 | **<0.001** |
| Total time spent online | 0.03 | -0.04–0.09 | 0.422 |  |  |  |
| Daytime nighttime difference | 0.09 | -0.02–0.19 | 0.095 | 0.10 | -0.00–0.21 | 0.060 |
| Time spent on entertainment |  |  |  | 0.14 | -0.06–0.34 | 0.167 |
| Time spent on social media |  |  |  | -0.05 | -0.23–0.13 | 0.567 |
| Time spent on messaging |  |  |  | 0.23 | -0.02–0.48 | 0.075 |
| Time spent on games |  |  |  | 0.00 | -0.11–0.12 | 0.944 |
| Time spent on shopping |  |  |  | 0.09 | -0.32–0.50 | 0.668 |
| Time spent on productivity |  |  |  | -0.00 | -0.41–0.41 | 0.996 |
| Time spent on news |  |  |  | -0.37 | -1.37–0.62 | 0.462 |
| **Random Effects** | | | | | | |
| σ^2^ | 11.43 | | | 11.43 | | |
| τ_00_ | 37.55_pid_ | | | 37.60 _pid_ | | |
| ICC | 0.77 | | | 0.77 | | |
| N | 305 _pid_ | | | 305 _pid_ | | |
| Observations | 1049 | | | 1049 | | |
| **** p<0.05 ** p<0.01 *** p<0.001*** | | | | | | |

**Table S17**. Results from mixed-effects models for Male, based on 2 days of desktop usage data.

| **Predictors** | Model 1 | | | Model 2 | | |
| --- | --- | --- | --- | --- | --- | --- |
|  | Estimate | CI | p | Estimate | CI | p |
| Intercept | 20.58*** | 17.90–23.27 | **<0.001** | 20.42*** | 17.73–23.11 | **<0.001** |
| Survey wave | -0.11 | -0.28–0.06 | 0.202 | -0.10 | -0.27–0.07 | 0.246 |
| Age | -1.67** | -2.68– -0.66 | **0.001** | -1.62** | -2.63– -0.60 | **0.002** |
| Income | -1.05*** | -1.66– -0.45 | **<0.001** | -1.02** | -1.64– -0.41 | **0.001** |
| Total time spent online | -0.00 | -0.09–0.08 | 0.916 |  |  |  |
| Daytime nighttime difference | 0.07 | -0.04–0.19 | 0.195 | 0.07 | -0.04–0.19 | 0.230 |
| Time spent on entertainment |  |  |  | 0.06 | -0.08–0.19 | 0.412 |
| Time spent on adult content |  |  |  | -0.09 | -0.33–0.16 | 0.484 |
| Time spent on social media |  |  |  | 0.13 | -0.22–0.48 | 0.458 |
| Time spent on messaging |  |  |  | -0.63 | -1.64–0.38 | 0.224 |
| Time spent on games |  |  |  | -0.32 | -0.73–0.09 | 0.128 |
| Time spent on shopping |  |  |  | -0.16 | -0.58–0.26 | 0.461 |
| Time spent on productivity |  |  |  | 0.21 | -0.12–0.53 | 0.211 |
| Time spent on news |  |  |  | -0.52* | -1.02– -0.01 | **0.044** |
| **Random Effects** | | | | | | |
| σ^2^ | 10.43 | | | 10.29 | | |
| τ_00_ | 39.39_pid_ | | | 39.67 _pid_ | | |
| ICC | 0.79 | | | 0.79 | | |
| N | 270 _pid_ | | | 270 _pid_ | | |
| Observations | 670 | | | 670 | | |
| **** p<0.05 ** p<0.01 *** p<0.001*** | | | | | | |

**Table S18**. Results from mixed-effects models for Female, based on 2 days of desktop usage data.

| **Predictors** | Model 1 | | | Model 2 | | |
| --- | --- | --- | --- | --- | --- | --- |
|  | Estimate | CI | p | Estimate | CI | p |
| Intercept | 23.48*** | 20.66–26.30 | **<0.001** | 23.44*** | 20.62–26.26 | **<0.001** |
| Survey wave | -0.18 | -0.38–0.03 | 0.088 | -0.14 | -0.35–0.07 | 0.187 |
| Age | -2.23*** | -3.27– -1.19 | **<0.001** | -2.16*** | -3.22– -1.10 | **<0.001** |
| Income | -1.04** | -1.75– -0.34 | **0.004** | -1.03** | -1.75– -0.31 | **0.005** |
| Total time spent online | -0.02 | -0.12–0.09 | 0.759 |  |  |  |
| Daytime nighttime difference | 0.01 | -0.13–0.15 | 0.873 | 0.05 | -0.11–0.20 | 0.555 |
| Time spent on entertainment |  |  |  | 0.05 | -0.11–0.21 | 0.561 |
| Time spent on adult content |  |  |  | -0.20 | -0.59–0.19 | 0.307 |
| Time spent on social media |  |  |  | 0.02 | -0.27–0.32 | 0.874 |
| Time spent on messaging |  |  |  | -0.59 | -1.44–0.27 | 0.180 |
| Time spent on games |  |  |  | -0.10 | -0.65–0.45 | 0.729 |
| Time spent on shopping |  |  |  | -0.05 | -0.51–0.41 | 0.835 |
| Time spent on productivity |  |  |  | -0.20 | -0.61–0.21 | 0.330 |
| Time spent on news |  |  |  | -0.49 | -1.13–0.15 | 0.133 |
| **Random Effects** | | | | | | |
| σ^2^ | 11.21 | | | 11.25 | | |
| τ_00_ | 41.93_pid_ | | | 41.81 _pid_ | | |
| ICC | 0.79 | | | 0.79 | | |
| N | 231 _pid_ | | | 231 _pid_ | | |
| Observations | 537 | | | 537 | | |
| **** p<0.05 ** p<0.01 *** p<0.001*** | | | | | | |

**Section 4**. Age differences.

**Table S19**. Results from mixed-effects models for Age 18–30, based on 30 days of mobile usage data.

| **Predictors** | Model 1 | | | Model 2 | | |
| --- | --- | --- | --- | --- | --- | --- |
|  | Estimate | CI | p | Estimate | CI | p |
| Intercept | 18.88*** | 15.54–22.22 | **<0.001** | 19.18*** | 15.85–22.51 | **<0.001** |
| Survey wave | -0.06 | -0.36–0.23 | 0.678 | -0.13 | -0.43–0.17 | 0.399 |
| Gender | -0.39 | -3.21–2.43 | 0.785 | -0.70 | -3.63–2.22 | 0.638 |
| Income | 0.29 | -0.67–1.24 | 0.558 | 0.30 | -0.67–1.27 | 0.542 |
| Total time spent online | -0.00 | -0.01–0.01 | 0.970 |  |  |  |
| Daytime nighttime difference | -0.00 | -0.03–0.03 | 0.980 | -0.01 | -0.05–0.03 | 0.591 |
| Time spent on entertainment |  |  |  | -0.01 | -0.06–0.03 | 0.550 |
| Time spent on social media |  |  |  | -0.00 | -0.03–0.03 | 0.972 |
| Time spent on messaging |  |  |  | -0.04 | -0.09–0.02 | 0.200 |
| Time spent on games |  |  |  | 0.00 | -0.05–0.05 | 0.958 |
| Time spent on shopping |  |  |  | 0.12 | -0.02–0.25 | 0.085 |
| Time spent on productivity |  |  |  | 0.04 | -0.04–0.12 | 0.352 |
| Time spent on news |  |  |  | 0.06 | -0.23–0.36 | 0.667 |
| **Random Effects** | | | | | | |
| σ^2^ | 10.12 | | | 10.16 | | |
| τ_00_ | 20.13_pid_ | | | 20.18 _pid_ | | |
| ICC | 0.67 | | | 0.67 | | |
| N | 53 _pid_ | | | 53 _pid_ | | |
| Observations | 197 | | | 197 | | |
| **** p<0.05 ** p<0.01 *** p<0.001*** | | | | | | |

**Table S20**. Results from mixed-effects models for age 31-45, based on 30 days of mobile usage data.

| **Predictors** | Model 1 | | | Model 2 | | |
| --- | --- | --- | --- | --- | --- | --- |
|  | Estimate | CI | p | Estimate | CI | p |
| Intercept | 19.39*** | 17.24–21.53 | **<0.001** | 19.54*** | 17.39–21.69 | **<0.001** |
| Survey wave | -0.14* | -0.28– -0.01 | **0.040** | -0.13 | -0.27–0.00 | 0.054 |
| Gender | 1.64* | 0.10–3.19 | **0.037** | 1.60* | 0.03–3.17 | **0.046** |
| Income | -1.27*** | -1.89– -0.66 | **<0.001** | -1.24*** | -1.85– -0.63 | **<0.001** |
| Total time spent online | 0.01 | -0.00–0.01 | 0.204 |  |  |  |
| Daytime nighttime difference | -0.00 | -0.02–0.02 | 0.875 | 0.00 | -0.01–0.02 | 0.631 |
| Time spent on entertainment |  |  |  | 0.02 | -0.00–0.04 | 0.075 |
| Time spent on social media |  |  |  | -0.00 | -0.02–0.02 | 0.964 |
| Time spent on messaging |  |  |  | 0.03 | -0.00–0.06 | 0.066 |
| Time spent on games |  |  |  | 0.00 | -0.01–0.02 | 0.587 |
| Time spent on shopping |  |  |  | 0.02 | -0.04–0.09 | 0.539 |
| Time spent on productivity |  |  |  | -0.08** | -0.14– -0.02 | **0.007** |
| Time spent on news |  |  |  | -0.03 | -0.13–0.08 | 0.597 |
| **Random Effects** | | | | | | |
| σ^2^ | 12.02 | | | 11.94 | | |
| τ_00_ | 33.13_pid_ | | | 33.04 _pid_ | | |
| ICC | 0.73 | | | 0.73 | | |
| N | 246 _pid_ | | | 246 _pid_ | | |
| Observations | 974 | | | 974 | | |
| **** p<0.05 ** p<0.01 *** p<0.001*** | | | | | | |

**Table S21**. Results from mixed-effects models for Age 46–60, based on 30 days of mobile usage data.

| **Predictors** | Model 1 | | | Model 2 | | |
| --- | --- | --- | --- | --- | --- | --- |
|  | Estimate | CI | p | Estimate | CI | p |
| Intercept | 18.13*** | 15.96–20.30 | **<0.001** | 18.05*** | 15.87–20.24 | **<0.001** |
| Survey wave | -0.09 | -0.23–0.05 | 0.198 | -0.09 | -0.23–0.05 | 0.219 |
| Gender | 2.31** | 0.63–3.98 | **0.007** | 2.23** | 0.54–3.92 | **0.010** |
| Income | -1.12** | -1.79– -0.45 | **0.001** | -1.05** | -1.73– -0.37 | **0.002** |
| Total time spent online | -0.01 | -0.01–0.00 | 0.215 |  |  |  |
| Daytime nighttime difference | -0.00 | -0.02–0.01 | 0.551 | -0.00 | -0.02–0.01 | 0.566 |
| Time spent on entertainment |  |  |  | -0.00 | -0.03–0.03 | 0.978 |
| Time spent on social media |  |  |  | 0.00 | -0.02–0.02 | 0.774 |
| Time spent on messaging |  |  |  | -0.02 | -0.05–0.01 | 0.158 |
| Time spent on games |  |  |  | -0.00 | -0.01–0.01 | 0.965 |
| Time spent on shopping |  |  |  | 0.01 | -0.06–0.08 | 0.788 |
| Time spent on productivity |  |  |  | -0.03 | -0.08–0.02 | 0.244 |
| Time spent on news |  |  |  | -0.04 | -0.14–0.05 | 0.384 |
| **Random Effects** | | | | | | |
| σ^2^ | 12.53 | | | 12.55 | | |
| τ_00_ | 39.64_pid_ | | | 39.97 _pid_ | | |
| ICC | 0.76 | | | 0.76 | | |
| N | 247 _pid_ | | | 247 _pid_ | | |
| Observations | 991 | | | 991 | | |
| **** p<0.05 ** p<0.01 *** p<0.001*** | | | | | | |

**Table S22**. Results from mixed-effects models for Age 60+, based on 30 days of mobile usage data.

| **Predictors** | Model 1 | | | Model 2 | | |
| --- | --- | --- | --- | --- | --- | --- |
|  | Estimate | CI | p | Estimate | CI | p |
| Intercept | 15.59*** | 12.33–18.84 | **<0.001** | 15.58*** | 12.29–18.87 | **<0.001** |
| Survey wave | -0.07 | -0.25–0.11 | 0.442 | -0.09 | -0.26–0.09 | 0.348 |
| Gender | 1.45 | -1.24–4.15 | 0.291 | 1.12 | -1.65–3.89 | 0.428 |
| Income | -1.44** | -2.49– -0.39 | **0.007** | -1.42** | -2.48– -0.35 | **0.009** |
| Total time spent online | 0.01* | 0.00–0.03 | **0.029** |  |  |  |
| Daytime nighttime difference | -0.03* | -0.05– -0.01 | **0.012** | -0.03* | -0.05– -0.01 | **0.012** |
| Time spent on entertainment |  |  |  | -0.00 | -0.06–0.06 | 0.986 |
| Time spent on social media |  |  |  | 0.01 | -0.03–0.04 | 0.722 |
| Time spent on messaging |  |  |  | 0.05 | -0.00–0.09 | 0.057 |
| Time spent on games |  |  |  | 0.02 | -0.01–0.04 | 0.169 |
| Time spent on shopping |  |  |  | 0.10* | 0.02–0.19 | **0.021** |
| Time spent on productivity |  |  |  | -0.01 | -0.07–0.05 | 0.769 |
| Time spent on news |  |  |  | -0.02 | -0.15–0.10 | 0.700 |
| **Random Effects** | | | | | | |
| σ^2^ | 9.67 | | | 9.59 | | |
| τ_00_ | 42.62_pid_ | | | 43.17 _pid_ | | |
| ICC | 0.82 | | | 0.82 | | |
| N | 110 _pid_ | | | 110 _pid_ | | |
| Observations | 438 | | | 438 | | |
| **** p<0.05 ** p<0.01 *** p<0.001*** | | | | | | |

**Table S23**. Results from mixed-effects models for age 18-30, based on 30 days of desktop usage data.

| **Predictors** | Model 1 | | | Model 2 | | |
| --- | --- | --- | --- | --- | --- | --- |
|  | Estimate | CI | p | Estimate | CI | p |
| Intercept | 16.85*** | 10.82–22.89 | **<0.001** | 17.39*** | 10.99–23.79 | **<0.001** |
| Survey wave | -0.08 | -0.66–0.49 | 0.773 | 0.18 | -0.44–0.81 | 0.565 |
| Gender | 2.88 | -1.80–7.56 | 0.228 | 1.83 | -3.26–6.92 | 0.480 |
| Income | 0.02 | -1.51–1.55 | 0.976 | -0.12 | -1.76–1.53 | 0.891 |
| Total time spent online | 0.01 | -0.03–0.05 | 0.724 |  |  |  |
| Daytime nighttime difference | 0.06 | -0.00–0.13 | 0.063 | 0.07 | -0.00–0.14 | 0.058 |
| Time spent on entertainment |  |  |  | 0.05 | -0.05–0.16 | 0.316 |
| Time spent on adult content |  |  |  | -0.87 | -1.77–0.03 | 0.058 |
| Time spent on social media |  |  |  | -0.15 | -0.49–0.19 | 0.380 |
| Time spent on messaging |  |  |  | -0.25 | -0.83–0.33 | 0.394 |
| Time spent on games |  |  |  | 1.08 | -0.25–2.40 | 0.111 |
| Time spent on shopping |  |  |  | 0.11 | -0.34–0.56 | 0.645 |
| Time spent on productivity |  |  |  | -0.08 | -0.28–0.13 | 0.466 |
| Time spent on news |  |  |  | 1.19 | -0.65–3.04 | 0.205 |
| **Random Effects** | | | | | | |
| σ^2^ | 12.96 | | | 11.68 | | |
| τ_00_ | 33.07_pid_ | | | 36.07 _pid_ | | |
| ICC | 0.72 | | | 0.76 | | |
| N | 38 _pid_ | | | 38 _pid_ | | |
| Observations | 85 | | | 85 | | |
| **** p<0.05 ** p<0.01 *** p<0.001*** | | | | | | |

**Table S24**. Results from mixed-effects models for Age 31–45, based on 30 days of desktop usage data.

| **Predictors** | Model 1 | | | Model 2 | | |
| --- | --- | --- | --- | --- | --- | --- |
|  | Estimate | CI | p | Estimate | CI | p |
| Intercept | 19.49*** | 17.10–21.88 | **<0.001** | 19.29*** | 16.90–21.68 | **<0.001** |
| Survey wave | -0.42*** | -0.65– -0.19 | **<0.001** | -0.38** | -0.61– -0.14 | **0.002** |
| Gender | 2.92** | 0.85–4.98 | **0.006** | 2.49* | 0.37–4.60 | **0.021** |
| Income | -0.96* | -1.72– -0.20 | **0.013** | -0.87* | -1.63– -0.10 | **0.027** |
| Total time spent online | -0.01 | -0.01–0.00 | 0.207 |  |  |  |
| Daytime nighttime difference | -0.01 | -0.03–0.01 | 0.549 | 0.00 | -0.02–0.02 | 0.914 |
| Time spent on entertainment |  |  |  | 0.00 | -0.01–0.01 | 0.777 |
| Time spent on adult content |  |  |  | -0.01 | -0.03–0.02 | 0.615 |
| Time spent on social media |  |  |  | 0.01 | -0.06–0.09 | 0.736 |
| Time spent on messaging |  |  |  | 0.10 | -0.14–0.34 | 0.406 |
| Time spent on games |  |  |  | 0.00 | -0.05–0.06 | 0.852 |
| Time spent on shopping |  |  |  | -0.01 | -0.07–0.06 | 0.859 |
| Time spent on productivity |  |  |  | 0.01 | -0.04–0.07 | 0.665 |
| Time spent on news |  |  |  | -0.26** | -0.42– -0.10 | **0.002** |
| **Random Effects** | | | | | | |
| σ^2^ | 10.26 | | | 10.03 | | |
| τ_00_ | 31.95_pid_ | | | 32.95 _pid_ | | |
| ICC | 0.76 | | | 0.77 | | |
| N | 137 _pid_ | | | 137 _pid_ | | |
| Observations | 395 | | | 395 | | |
| **** p<0.05 ** p<0.01 *** p<0.001*** | | | | | | |

**Table S25**. Results from mixed-effects models for age 46-60, based on 30 days of desktop usage data.

| **Predictors** | Model 1 | | | Model 2 | | |
| --- | --- | --- | --- | --- | --- | --- |
|  | Estimate | CI | p | Estimate | CI | p |
| Intercept | 17.29*** | 15.05–19.54 | **<0.001** | 17.37*** | 15.11–19.63 | **<0.001** |
| Survey wave | -0.08 | -0.24–0.07 | 0.281 | -0.10 | -0.26–0.05 | 0.191 |
| Gender | 1.49 | -0.42–3.41 | 0.127 | 1.28 | -0.64–3.20 | 0.192 |
| Income | -1.04** | -1.76– -0.33 | **0.004** | -1.04** | -1.77– -0.31 | **0.005** |
| Total time spent online | 0.00 | -0.01–0.01 | 0.824 |  |  |  |
| Daytime nighttime difference | 0.01 | -0.01–0.02 | 0.397 | 0.01 | -0.01–0.02 | 0.332 |
| Time spent on entertainment |  |  |  | -0.01 | -0.04–0.01 | 0.348 |
| Time spent on adult content |  |  |  | -0.02 | -0.05–0.01 | 0.162 |
| Time spent on social media |  |  |  | 0.01 | -0.02–0.04 | 0.448 |
| Time spent on messaging |  |  |  | 0.00 | -0.06–0.06 | 0.958 |
| Time spent on games |  |  |  | -0.02 | -0.07–0.03 | 0.385 |
| Time spent on shopping |  |  |  | 0.04 | -0.00–0.09 | 0.062 |
| Time spent on productivity |  |  |  | -0.01 | -0.05–0.03 | 0.582 |
| Time spent on news |  |  |  | 0.02 | -0.04–0.08 | 0.504 |
| **Random Effects** | | | | | | |
| σ^2^ | 11.36 | | | 11.36 | | |
| τ_00_ | 49.33_pid_ | | | 49.04 _pid_ | | |
| ICC | 0.81 | | | 0.81 | | |
| N | 232 _pid_ | | | 232 _pid_ | | |
| Observations | 790 | | | 790 | | |
| **** p<0.05 ** p<0.01 *** p<0.001*** | | | | | | |

**Table S26**. Results from mixed-effects models for individuals older than 60 years, based on 30 days of desktop usage data.

| **Predictors** | Model 1 | | | Model 2 | | |
| --- | --- | --- | --- | --- | --- | --- |
|  | Estimate | CI | p | Estimate | CI | p |
| Intercept | 16.15*** | 13.35–18.94 | **<0.001** | 16.37*** | 13.54–19.20 | **<0.001** |
| Survey wave | -0.06 | -0.26–0.13 | 0.536 | -0.07 | -0.26–0.13 | 0.523 |
| Gender | 1.34 | -1.07–3.76 | 0.276 | 1.03 | -1.43–3.48 | 0.413 |
| Income | -1.31** | -2.24– -0.38 | **0.006** | -1.33** | -2.29– -0.37 | **0.006** |
| Total time spent online | -0.00 | -0.02–0.01 | 0.722 |  |  |  |
| Daytime nighttime difference | -0.01 | -0.03–0.01 | 0.435 | -0.01 | -0.04–0.01 | 0.264 |
| Time spent on entertainment |  |  |  | -0.04 | -0.09–0.01 | 0.144 |
| Time spent on adult content |  |  |  | -0.19 | -0.38–0.00 | 0.055 |
| Time spent on social media |  |  |  | -0.01 | -0.03–0.02 | 0.652 |
| Time spent on messaging |  |  |  | 0.07 | -0.04–0.18 | 0.220 |
| Time spent on games |  |  |  | 0.04 | -0.02–0.10 | 0.193 |
| Time spent on shopping |  |  |  | 0.03 | -0.04–0.11 | 0.404 |
| Time spent on productivity |  |  |  | 0.02 | -0.03–0.06 | 0.505 |
| Time spent on news |  |  |  | -0.04 | -0.09–0.01 | 0.150 |
| **Random Effects** | | | | | | |
| σ^2^ | 11.00 | | | 10.81 | | |
| τ_00_ | 35.97_pid_ | | | 36.63 _pid_ | | |
| ICC | 0.77 | | | 0.77 | | |
| N | 119 _pid_ | | | 119 _pid_ | | |
| Observations | 443 | | | 443 | | |
| **** p<0.05 ** p<0.01 *** p<0.001*** | | | | | | |

**Table S27**. Results from mixed-effects models for age 18-30 years, based on 2 days of mobile usage data.

| **Predictors** | Model 1 | | | Model 2 | | |
| --- | --- | --- | --- | --- | --- | --- |
|  | Estimate | CI | p | Estimate | CI | p |
| Intercept | 18.96*** | 15.32–22.61 | **<0.001** | 19.09*** | 15.33–22.85 | **<0.001** |
| Survey wave | -0.21 | -0.56–0.14 | 0.236 | -0.24 | -0.62–0.13 | 0.205 |
| Gender | -0.16 | -3.20–2.88 | 0.919 | -0.16 | -3.29–2.98 | 0.923 |
| Income | 0.22 | -0.85–1.28 | 0.688 | 0.16 | -0.93–1.26 | 0.767 |
| Total time spent online | 0.00 | -0.12–0.13 | 0.944 |  |  |  |
| Daytime nighttime difference | 0.08 | -0.19–0.36 | 0.547 | 0.12 | -0.17–0.42 | 0.421 |
| Time spent on entertainment |  |  |  | -0.11 | -0.55–0.32 | 0.612 |
| Time spent on social media |  |  |  | -0.02 | -0.40–0.36 | 0.904 |
| Time spent on messaging |  |  |  | 0.15 | -0.40–0.69 | 0.597 |
| Time spent on games |  |  |  | 0.06 | -0.44–0.56 | 0.800 |
| Time spent on shopping |  |  |  | 1.18 | -0.43–2.79 | 0.150 |
| Time spent on productivity |  |  |  | -0.72 | -1.79–0.35 | 0.187 |
| Time spent on news |  |  |  | -0.06 | -2.69–2.56 | 0.962 |
| **Random Effects** | | | | | | |
| σ^2^ | 10.97 | | | 11.02 | | |
| τ_00_ | 20.90_pid_ | | | 21.79 _pid_ | | |
| ICC | 0.66 | | | 0.66 | | |
| N | 48 _pid_ | | | 48 _pid_ | | |
| Observations | 163 | | | 163 | | |
| **** p<0.05 ** p<0.01 *** p<0.001*** | | | | | | |

**Table S28.** Results from mixed-effects models for age 31-45 years, based on 2 days of mobile usage data.

| **Predictors** | Model 1 | | | Model 2 | | |
| --- | --- | --- | --- | --- | --- | --- |
|  | Estimate | CI | p | Estimate | CI | p |
| Intercept | 19.86*** | 17.61–22.11 | **<0.001** | 19.89*** | 17.65–22.12 | **<0.001** |
| Survey wave | -0.13 | -0.29–0.02 | 0.085 | -0.15 | -0.30–0.01 | 0.062 |
| Gender | 1.20 | -0.48–2.87 | 0.162 | 1.03 | -0.65–2.72 | 0.230 |
| Income | -1.33*** | -1.99– -0.66 | **<0.001** | -1.25*** | -1.91– -0.58 | **<0.001** |
| Total time spent online | 0.01 | -0.07–0.08 | 0.892 |  |  |  |
| Daytime nighttime difference | 0.14* | 0.02–0.25 | **0.018** | 0.14* | 0.02–0.25 | **0.020** |
| Time spent on entertainment |  |  |  | 0.22* | 0.01–0.43 | **0.039** |
| Time spent on social media |  |  |  | 0.08 | -0.14–0.30 | 0.497 |
| Time spent on messaging |  |  |  | 0.11 | -0.15–0.36 | 0.418 |
| Time spent on games |  |  |  | 0.00 | -0.14–0.14 | 1.000 |
| Time spent on shopping |  |  |  | -0.54* | -1.08– -0.00 | **0.050** |
| Time spent on productivity |  |  |  | -0.16 | -0.60–0.28 | 0.468 |
| Time spent on news |  |  |  | -1.01 | -2.22–0.19 | 0.098 |
| **Random Effects** | | | | | | |
| σ^2^ | 11.79 | | | 11.72 | | |
| τ_00_ | 37.92_pid_ | | | 37.46 _pid_ | | |
| ICC | 0.76 | | | 0.76 | | |
| N | 237 _pid_ | | | 237 _pid_ | | |
| Observations | 821 | | | 821 | | |
| **** p<0.05 ** p<0.01 *** p<0.001*** | | | | | | |

**Table S29**. Results from mixed-effects models for Age 46–60, based on 2 days of mobile usage data.

| **Predictors** | Model 1 | | | Model 2 | | |
| --- | --- | --- | --- | --- | --- | --- |
|  | Estimate | CI | p | Estimate | CI | p |
| Intercept | 17.35*** | 15.19–19.50 | **<0.001** | 17.29*** | 15.13–19.45 | **<0.001** |
| Survey wave | -0.12 | -0.28–0.04 | 0.151 | -0.11 | -0.28–0.05 | 0.189 |
| Gender | 2.69** | 1.00–4.39 | **0.002** | 2.66** | 0.95–4.36 | **0.002** |
| Income | -1.10** | -1.78– -0.42 | **0.002** | -1.07** | -1.75– -0.38 | **0.002** |
| Total time spent online | -0.03 | -0.11–0.05 | 0.467 |  |  |  |
| Daytime nighttime difference | -0.03 | -0.15–0.09 | 0.589 | -0.03 | -0.16–0.09 | 0.622 |
| Time spent on entertainment |  |  |  | -0.11 | -0.34–0.11 | 0.326 |
| Time spent on social media |  |  |  | -0.01 | -0.22–0.20 | 0.955 |
| Time spent on messaging |  |  |  | 0.06 | -0.22–0.34 | 0.678 |
| Time spent on games |  |  |  | -0.03 | -0.17–0.10 | 0.626 |
| Time spent on shopping |  |  |  | -0.04 | -0.53–0.46 | 0.888 |
| Time spent on productivity |  |  |  | 0.04 | -0.39–0.46 | 0.869 |
| Time spent on news |  |  |  | -0.85 | -1.76–0.06 | 0.068 |
| **Random Effects** | | | | | | |
| σ^2^ | 13.08 | | | 13.09 | | |
| τ_00_ | 40.25_pid_ | | | 40.43 _pid_ | | |
| ICC | 0.75 | | | 0.76 | | |
| N | 246 _pid_ | | | 246 _pid_ | | |
| Observations | 813 | | | 813 | | |
| **** p<0.05 ** p<0.01 *** p<0.001*** | | | | | | |

**Table S30.** Results from mixed-effects models for individuals older than 60 years, based on 2 days of mobile usage data.

| **Predictors** | Model 1 | | | Model 2 | | |
| --- | --- | --- | --- | --- | --- | --- |
|  | Estimate | CI | p | Estimate | CI | p |
| Intercept | 15.16*** | 11.84–18.47 | **<0.001** | 15.20*** | 11.89–18.51 | **<0.001** |
| Survey wave | 0.06 | -0.17–0.29 | 0.608 | 0.05 | -0.18–0.28 | 0.651 |
| Gender | 2.67 | -0.22–5.55 | 0.070 | 2.41 | -0.49–5.30 | 0.104 |
| Income | -1.60** | -2.74– -0.45 | **0.006** | -1.65** | -2.80– -0.50 | **0.005** |
| Total time spent online | 0.10 | -0.03–0.24 | 0.121 |  |  |  |
| Daytime nighttime difference | 0.01 | -0.16–0.19 | 0.870 | -0.02 | -0.20–0.16 | 0.818 |
| Time spent on entertainment |  |  |  | -0.10 | -0.55–0.36 | 0.671 |
| Time spent on social media |  |  |  | -0.19 | -0.58–0.20 | 0.337 |
| Time spent on messaging |  |  |  | 0.40 | -0.08–0.88 | 0.101 |
| Time spent on games |  |  |  | 0.26* | 0.01–0.50 | **0.043** |
| Time spent on shopping |  |  |  | 0.72 | -0.03–1.48 | 0.061 |
| Time spent on productivity |  |  |  | 0.05 | -0.32–0.43 | 0.790 |
| Time spent on news |  |  |  | 0.08 | -0.75–0.90 | 0.857 |
| **Random Effects** | | | | | | |
| σ^2^ | 10.59 | | | 10.47 | | |
| τ_00_ | 48.26_pid_ | | | 48.33 _pid_ | | |
| ICC | 0.82 | | | 0.82 | | |
| N | 109 _pid_ | | | 109 _pid_ | | |
| Observations | 344 | | | 344 | | |
| **** p<0.05 ** p<0.01 *** p<0.001*** | | | | | | |

**Table S31**. Results from mixed-effects models for age 18-30 years, based on 2 days of desktop usage data.

| **Predictors** | Model 1 | | | Model 2 | | |
| --- | --- | --- | --- | --- | --- | --- |
|  | Estimate | CI | p | Estimate | CI | p |
| Intercept | 18.99*** | 11.49–26.48 | **<0.001** | 18.15*** | 9.40–26.91 | **<0.001** |
| Survey wave | -0.25 | -1.16–0.65 | 0.581 | -0.59 | -1.62–0.44 | 0.261 |
| Gender | 2.53 | -2.85–7.92 | 0.356 | 3.51 | -2.66–9.68 | 0.265 |
| Income | -0.58 | -2.61–1.45 | 0.575 | -0.39 | -2.51–1.73 | 0.719 |
| Total time spent online | 0.36 | -0.35–1.07 | 0.321 |  |  |  |
| Daytime nighttime difference | -0.20 | -1.14–0.75 | 0.685 | -0.60 | -1.84–0.63 | 0.339 |
| Time spent on entertainment |  |  |  | 1.14 | -0.18–2.46 | 0.090 |
| Time spent on adult content |  |  |  | 1.10 | -6.74–8.94 | 0.783 |
| Time spent on social media |  |  |  | -3.17 | -10.47–4.14 | 0.395 |
| Time spent on messaging |  |  |  | 15.11 | -20.88–51.10 | 0.411 |
| Time spent on games |  |  |  | 139.88 | -31.83–311.59 | 0.110 |
| Time spent on shopping |  |  |  | -0.42 | -4.22–3.38 | 0.827 |
| Time spent on productivity |  |  |  | 0.82 | -2.02–3.66 | 0.571 |
| Time spent on news |  |  |  | 13.35 | -18.58–45.27 | 0.413 |
| **Random Effects** | | | | | | |
| σ^2^ | 9.88 | | | 11.20 | | |
| τ_00_ | 37.62_pid_ | | | 35.96 _pid_ | | |
| ICC | 0.79 | | | 0.76 | | |
| N | 28 _pid_ | | | 28 _pid_ | | |
| Observations | 45 | | | 45 | | |
| **** p<0.05 ** p<0.01 *** p<0.001*** | | | | | | |

**Table S32**. Results from mixed-effects models for age 31-45 years, based on 2 days of desktop usage data.

| **Predictors** | Model 1 | | | Model 2 | | |
| --- | --- | --- | --- | --- | --- | --- |
|  | Estimate | CI | p | Estimate | CI | p |
| Intercept | 17.10*** | 14.25–19.95 | **<0.001** | 16.70*** | 13.87–19.53 | **<0.001** |
| Survey wave | -0.14 | -0.39–0.12 | 0.293 | -0.11 | -0.37–0.15 | 0.414 |
| Gender | 3.20* | 0.76–5.63 | **0.010** | 3.33** | 0.92–5.75 | **0.007** |
| Income | -0.64 | -1.56–0.27 | 0.166 | -0.63 | -1.53–0.28 | 0.173 |
| Total time spent online | 0.05 | -0.05–0.16 | 0.296 |  |  |  |
| Daytime nighttime difference | 0.00 | -0.15–0.16 | 0.965 | -0.01 | -0.17–0.15 | 0.918 |
| Time spent on entertainment |  |  |  | 0.04 | -0.08–0.16 | 0.475 |
| Time spent on adult content |  |  |  | 0.01 | -0.26–0.27 | 0.954 |
| Time spent on social media |  |  |  | 1.22* | 0.15–2.29 | **0.025** |
| Time spent on messaging |  |  |  | 3.05 | -0.65–6.76 | 0.106 |
| Time spent on games |  |  |  | -0.09 | -0.73–0.55 | 0.789 |
| Time spent on shopping |  |  |  | -0.06 | -0.74–0.62 | 0.867 |
| Time spent on productivity |  |  |  | 0.05 | -0.55–0.65 | 0.876 |
| Time spent on news |  |  |  | -0.77 | -2.19–0.66 | 0.291 |
| **Random Effects** | | | | | | |
| σ^2^ | 8.33 | | | 8.58 | | |
| τ_00_ | 42.44_pid_ | | | 40.66 _pid_ | | |
| ICC | 0.84 | | | 0.83 | | |
| N | 126 _pid_ | | | 126 _pid_ | | |
| Observations | 265 | | | 265 | | |
| **** p<0.05 ** p<0.01 *** p<0.001*** | | | | | | |

**Table S33**. Results from mixed-effects models for age 46-60 years, based on 2 days of desktop usage data.

| **Predictors** | Model 1 | | | Model 2 | | |
| --- | --- | --- | --- | --- | --- | --- |
|  | Estimate | CI | p | Estimate | CI | p |
| Intercept | 18.09*** | 15.80–20.39 | **<0.001** | 18.19*** | 15.88–20.51 | **<0.001** |
| Survey wave | -0.09 | -0.29–0.11 | 0.385 | -0.09 | -0.29–0.11 | 0.377 |
| Gender | 0.43 | -1.47–2.33 | 0.657 | 0.42 | -1.48–2.32 | 0.662 |
| Income | -1.15** | -1.86– -0.43 | **0.002** | -1.14** | -1.86– -0.41 | **0.002** |
| Total time spent online | -0.02 | -0.13–0.09 | 0.737 |  |  |  |
| Daytime nighttime difference | 0.08 | -0.05–0.22 | 0.236 | 0.09 | -0.05–0.23 | 0.224 |
| Time spent on entertainment |  |  |  | 0.10 | -0.10–0.30 | 0.312 |
| Time spent on adult content |  |  |  | -0.25 | -0.57–0.07 | 0.127 |
| Time spent on social media |  |  |  | 0.21 | -0.16–0.59 | 0.266 |
| Time spent on messaging |  |  |  | -0.78* | -1.48– -0.07 | **0.031** |
| Time spent on games |  |  |  | -0.35 | -0.83–0.14 | 0.159 |
| Time spent on shopping |  |  |  | -0.20 | -0.64–0.23 | 0.364 |
| Time spent on productivity |  |  |  | 0.09 | -0.26–0.44 | 0.608 |
| Time spent on news |  |  |  | -0.53 | -1.25–0.19 | 0.148 |
| **Random Effects** | | | | | | |
| σ^2^ | 12.55 | | | 12.40 | | |
| τ_00_ | 44.89_pid_ | | | 44.55 _pid_ | | |
| ICC | 0.78 | | | 0.78 | | |
| N | 226 _pid_ | | | 226 _pid_ | | |
| Observations | 569 | | | 569 | | |
| **** p<0.05 ** p<0.01 *** p<0.001*** | | | | | | |

**Table S34**. Results from mixed-effects models for individuals older than 60 years, based on 2 days of desktop usage data.

| **Predictors** | Model 1 | | | Model 2 | | |
| --- | --- | --- | --- | --- | --- | --- |
|  | Estimate | CI | p | Estimate | CI | p |
| Intercept | 16.68*** | 14.09–19.26 | **<0.001** | 16.62*** | 14.03–19.22 | **<0.001** |
| Survey wave | -0.20 | -0.43–0.03 | 0.086 | -0.18 | -0.42–0.05 | 0.129 |
| Gender | 1.69 | -0.57–3.96 | 0.142 | 1.52 | -0.75–3.79 | 0.189 |
| Income | -1.44** | -2.32– -0.56 | **0.001** | -1.44** | -2.33– -0.55 | **0.002** |
| Total time spent online | -0.14 | -0.28–0.00 | 0.058 |  |  |  |
| Daytime nighttime difference | 0.10 | -0.08–0.27 | 0.286 | 0.10 | -0.09–0.28 | 0.305 |
| Time spent on entertainment |  |  |  | -0.04 | -0.36–0.29 | 0.829 |
| Time spent on adult content |  |  |  | -0.82 | -2.48–0.85 | 0.337 |
| Time spent on social media |  |  |  | -0.15 | -0.44–0.13 | 0.296 |
| Time spent on messaging |  |  |  | 7.63* | 0.39–14.87 | **0.039** |
| Time spent on games |  |  |  | -0.30 | -0.95–0.35 | 0.372 |
| Time spent on shopping |  |  |  | 0.02 | -0.61–0.65 | 0.957 |
| Time spent on productivity |  |  |  | -0.23 | -0.70–0.25 | 0.349 |
| Time spent on news |  |  |  | -0.46 | -0.96–0.04 | 0.071 |
| **Random Effects** | | | | | | |
| σ^2^ | 9.41 | | | 9.43 | | |
| τ_00_ | 33.35_pid_ | | | 33.02_pid_ | | |
| ICC | 0.78 | | | 0.78 | | |
| N | 121 _pid_ | | | 121 _pid_ | | |
| Observations | 328 | | | 328 | | |
| ** p<0.05 ** p<0.01 *** p<0.001* | | | | | | |

**Section 5**. Income differences.

**Table S35**. Results from mixed-effects models for the Tier I income group, based on 30 days of mobile usage data.

| **Predictors** | Model 1 | | | Model 2 | | |
| --- | --- | --- | --- | --- | --- | --- |
|  | Estimate | CI | p | Estimate | CI | p |
| Intercept | 20.27*** | 15.68–24.85 | **<0.001** | 20.63*** | 16.08–25.19 | **<0.001** |
| Survey wave | 0.01 | -0.25–0.26 | 0.969 | -0.05 | -0.31–0.21 | 0.699 |
| Gender | -0.07 | -3.96–3.83 | 0.974 | -0.62 | -4.44–3.20 | 0.749 |
| Age | -1.03 | -2.94–0.88 | 0.291 | -1.12 | -2.99–0.75 | 0.239 |
| Total time spent online | -0.00 | -0.01–0.01 | 0.844 |  |  |  |
| Daytime nighttime difference | 0.02 | -0.01–0.05 | 0.147 | 0.02 | -0.01–0.06 | 0.146 |
| Time spent on entertainment |  |  |  | 0.02 | -0.02–0.06 | 0.361 |
| Time spent on social media |  |  |  | 0.02 | -0.02–0.07 | 0.301 |
| Time spent on messaging |  |  |  | -0.06* | -0.12– -0.01 | **0.030** |
| Time spent on games |  |  |  | -0.00 | -0.02–0.02 | 0.930 |
| Time spent on shopping |  |  |  | 0.07 | -0.05–0.19 | 0.225 |
| Time spent on productivity |  |  |  | -0.04 | -0.13–0.04 | 0.305 |
| Time spent on news |  |  |  | 0.08 | -0.28–0.45 | 0.657 |
| **Random Effects** | | | | | | |
| σ^2^ | 9.85 | | | 9.87 | | |
| τ_00_ | 53.36 _pid_ | | | 50.26 _pid_ | | |
| ICC | 0.84 | | | 0.84 | | |
| N | 60 _pid_ | | | 60 _pid_ | | |
| Observations | 246 | | | 246 | | |
| **** p<0.05 ** p<0.01 *** p<0.001*** | | | | | | |

**Table S36**: Results from mixed-effects models for the Tier II income group, based on 30 days of mobile usage data.

| **Predictors** | Model 1 | | | Model 2 | | |
| --- | --- | --- | --- | --- | --- | --- |
|  | Estimate | CI | p | Estimate | CI | p |
| Intercept | 18.35*** | 15.25–21.45 | **<0.001** | 18.38*** | 15.18–21.58 | **<0.001** |
| Survey wave | 0.05 | -0.15–0.24 | 0.627 | 0.07 | -0.13–0.26 | 0.515 |
| Gender | 0.80 | -1.59–3.19 | 0.511 | 0.79 | -1.65–3.23 | 0.525 |
| Age | -0.76 | -2.05–0.53 | 0.247 | -0.89 | -2.22–0.43 | 0.185 |
| Total time spent online | 0.00 | -0.01–0.01 | 0.705 |  |  |  |
| Daytime nighttime difference | -0.01 | -0.03–0.01 | 0.597 | -0.01 | -0.03–0.01 | 0.388 |
| Time spent on entertainment |  |  |  | 0.01 | -0.02–0.05 | 0.461 |
| Time spent on social media |  |  |  | -0.01 | -0.03–0.01 | 0.240 |
| Time spent on messaging |  |  |  | 0.02 | -0.03–0.08 | 0.382 |
| Time spent on games |  |  |  | 0.02 | -0.00–0.04 | 0.118 |
| Time spent on shopping |  |  |  | -0.03 | -0.14–0.08 | 0.582 |
| Time spent on productivity |  |  |  | 0.00 | -0.09–0.09 | 0.979 |
| Time spent on news |  |  |  | 0.05 | -0.15–0.25 | 0.619 |
| **Random Effects** | | | | | | |
| σ^2^ | 13.25 | | | 13.31 | | |
| τ_00_ | 40.42 _pid_ | | | 40.21 _pid_ | | |
| ICC | 0.75 | | | 0.75 | | |
| N | 129 _pid_ | | | 129 _pid_ | | |
| Observations | 518 | | | 518 | | |
| **** p<0.05 ** p<0.01 *** p<0.001*** | | | | | | |

**Table S37**. Results from mixed-effects models for the Tier III income group, based on 30 days of mobile usage data.

| **Predictors** | Model 1 | | | Model 2 | | |
| --- | --- | --- | --- | --- | --- | --- |
|  | Estimate | CI | p | Estimate | CI | p |
| Intercept | 17.96*** | 15.24–20.68 | **<0.001** | 17.61*** | 14.88–20.33 | **<0.001** |
| Survey wave | -0.09 | -0.26–0.07 | 0.256 | -0.10 | -0.27–0.06 | 0.213 |
| Gender | 4.37*** | 2.34–6.41 | **<0.001** | 3.84*** | 1.76–5.91 | **<0.001** |
| Age | -1.92** | -3.15– -0.69 | **0.002** | -1.59* | -2.84– -0.35 | **0.012** |
| Total time spent online | -0.00 | -0.01–0.01 | 0.719 |  |  |  |
| Daytime nighttime difference | -0.01 | -0.03–0.00 | 0.125 | -0.01 | -0.03–0.00 | 0.131 |
| Time spent on entertainment |  |  |  | -0.01 | -0.04–0.02 | 0.343 |
| Time spent on social media |  |  |  | 0.01 | -0.02–0.04 | 0.631 |
| Time spent on messaging |  |  |  | 0.02 | -0.02–0.06 | 0.286 |
| Time spent on games |  |  |  | 0.00 | -0.01–0.02 | 0.800 |
| Time spent on shopping |  |  |  | 0.05 | -0.03–0.12 | 0.204 |
| Time spent on productivity |  |  |  | -0.05 | -0.10–0.00 | 0.059 |
| Time spent on news |  |  |  | -0.10 | -0.20–0.01 | 0.078 |
| **Random Effects** | | | | | | |
| σ^2^ | 10.73 | | | 10.75 | | |
| τ_00_ | 39.60 _pid_ | | | 38.38 _pid_ | | |
| ICC | 0.79 | | | 0.78 | | |
| N | 166 _pid_ | | | 166 _pid_ | | |
| Observations | 654 | | | 654 | | |
| **** p<0.05 ** p<0.01 *** p<0.001*** | | | | | | |

**Table S38**: Results from mixed-effects models for the Tier IV income group, based on 30 days of mobile usage data.

| **Predictors** | Model 1 | | | Model 2 | | |
| --- | --- | --- | --- | --- | --- | --- |
|  | Estimate | CI | p | Estimate | CI | p |
| Intercept | 19.94*** | 17.44–22.44 | **<0.001** | 20.20*** | 17.65–22.75 | **<0.001** |
| Survey wave | -0.21* | -0.39– -0.03 | **0.020** | -0.21* | -0.39– -0.03 | **0.024** |
| Gender | 0.28 | -1.57–2.14 | 0.765 | 0.14 | -1.78–2.05 | 0.889 |
| Age | -2.51*** | -3.62– -1.40 | **<0.001** | -2.57*** | -3.71– -1.43 | **<0.001** |
| Total time spent online | 0.01* | 0.00–0.02 | **0.015** |  |  |  |
| Daytime nighttime difference | -0.00 | -0.02–0.02 | 0.966 | 0.00 | -0.02–0.03 | 0.856 |
| Time spent on entertainment |  |  |  | 0.01 | -0.03–0.04 | 0.795 |
| Time spent on social media |  |  |  | 0.01 | -0.02–0.04 | 0.668 |
| Time spent on messaging |  |  |  | 0.03 | -0.01–0.07 | 0.158 |
| Time spent on games |  |  |  | 0.01 | -0.01–0.03 | 0.359 |
| Time spent on shopping |  |  |  | 0.08* | 0.01–0.16 | **0.022** |
| Time spent on productivity |  |  |  | -0.03 | -0.10–0.04 | 0.435 |
| Time spent on news |  |  |  | 0.03 | -0.10–0.15 | 0.681 |
| **Random Effects** | | | | | | |
| σ^2^ | 10.59 | | | 10.58 | | |
| τ_00_ | 26.70 _pid_ | | | 27.33 _pid_ | | |
| ICC | 0.72 | | | 0.72 | | |
| N | 137 _pid_ | | | 137 _pid_ | | |
| Observations | 519 | | | 519 | | |
| **** p<0.05 ** p<0.01 *** p<0.001*** | | | | | | |

**Table S39**. Results from mixed-effects models for the Tier V income group, based on 30 days of mobile usage data.

| **Predictors** | Model 1 | | | Model 2 | | |
| --- | --- | --- | --- | --- | --- | --- |
|  | Estimate | CI | p | Estimate | CI | p |
| Intercept | 16.56*** | 13.99–19.14 | **<0.001** | 16.23*** | 13.59–18.87 | **<0.001** |
| Survey wave | -0.17 | -0.34–0.00 | 0.051 | -0.17 | -0.34–0.00 | 0.053 |
| Gender | 1.66 | -0.26–3.57 | 0.091 | 1.69 | -0.26–3.64 | 0.090 |
| Age | -1.77** | -3.00– -0.54 | **0.005** | -1.65* | -2.90– -0.39 | **0.010** |
| Total time spent online | 0.00 | -0.01–0.01 | 0.971 |  |  |  |
| Daytime nighttime difference | -0.02 | -0.04–0.01 | 0.170 | -0.02 | -0.04–0.01 | 0.161 |
| Time spent on entertainment |  |  |  | 0.02 | -0.01–0.05 | 0.116 |
| Time spent on social media |  |  |  | 0.01 | -0.03–0.04 | 0.615 |
| Time spent on messaging |  |  |  | -0.01 | -0.05–0.02 | 0.502 |
| Time spent on games |  |  |  | 0.00 | -0.02–0.02 | 0.750 |
| Time spent on shopping |  |  |  | 0.03 | -0.06–0.13 | 0.509 |
| Time spent on productivity |  |  |  | -0.03 | -0.09–0.03 | 0.309 |
| Time spent on news |  |  |  | -0.01 | -0.11–0.09 | 0.836 |
| **Random Effects** | | | | | | |
| σ^2^ | 12.70 | | | 12.72 | | |
| τ_00_ | 31.07 _pid_ | | | 31.24 _pid_ | | |
| ICC | 0.71 | | | 0.71 | | |
| N | 164 _pid_ | | | 164 _pid_ | | |
| Observations | 663 | | | 663 | | |
| **** p<0.05 ** p<0.01 *** p<0.001*** | | | | | | |

**Table S40**. Results from mixed-effects models for Tier I, based on 30 days of desktop usage data.

| **Predictors** | Model 1 | | | Model 2 | | |
| --- | --- | --- | --- | --- | --- | --- |
|  | Estimate | CI | p | Estimate | CI | p |
| Intercept | 19.98*** | 15.28–24.68 | **<0.001** | 19.93*** | 15.24–24.62 | **<0.001** |
| Survey wave | 0.02 | -0.28–0.33 | 0.883 | 0.03 | -0.28–0.34 | 0.843 |
| Gender | -0.03 | -3.87–3.80 | 0.986 | 0.30 | -3.55–4.16 | 0.878 |
| Age | -0.88 | -3.06–1.30 | 0.427 | -1.15 | -3.37–1.06 | 0.307 |
| Total time spent online | -0.00 | -0.01–0.01 | 0.825 |  |  |  |
| Daytime nighttime difference | -0.01 | -0.04–0.02 | 0.491 | -0.00 | -0.03–0.03 | 0.796 |
| Time spent on entertainment |  |  |  | 0.01 | -0.03–0.04 | 0.650 |
| Time spent on adult content |  |  |  | -0.01 | -0.03–0.01 | 0.427 |
| Time spent on social media |  |  |  | -0.00 | -0.03–0.03 | 0.902 |
| Time spent on messaging |  |  |  | -0.08 | -0.37–0.20 | 0.565 |
| Time spent on games |  |  |  | 0.01 | -0.05–0.06 | 0.852 |
| Time spent on shopping |  |  |  | 0.07 | -0.03–0.17 | 0.160 |
| Time spent on productivity |  |  |  | -0.07* | -0.13– -0.01 | **0.030** |
| Time spent on the news |  |  |  | 0.28* | 0.05–0.50 | **0.015** |
| **Random Effects** | | | | | | |
| σ^2^ | 10.64 | | | 10.27 | | |
| τ_00_ | 49.24 _pid_ | | | 48.75 _pid_ | | |
| ICC | 0.82 | | | 0.83 | | |
| N | 59 _pid_ | | | 59 _pid_ | | |
| Observations | 196 | | | 196 | | |
| **** p<0.05 ** p<0.01 *** p<0.001*** | | | | | | |

**Table S41**. Results from mixed-effects models for Tier II, based on 30 days of desktop usage data.

| **Predictors** | Model 1 | | | Model 2 | | |
| --- | --- | --- | --- | --- | --- | --- |
|  | Estimate | CI | p | Estimate | CI | p |
| Intercept | 18.86*** | 15.18–22.54 | **<0.001** | 18.64*** | 14.85–22.44 | **<0.001** |
| Survey wave | 0.03 | -0.18–0.24 | 0.768 | 0.01 | -0.21–0.22 | 0.951 |
| Gender | 2.05 | -0.72–4.83 | 0.147 | 2.23 | -0.61–5.08 | 0.124 |
| Age | -1.38 | -2.91–0.16 | 0.079 | -1.36 | -2.95–0.23 | 0.093 |
| Total time spent online | -0.01 | -0.02–0.00 | 0.129 |  |  |  |
| Daytime nighttime difference | 0.01 | -0.02–0.03 | 0.496 | 0.01 | -0.02–0.03 | 0.530 |
| Time spent on entertainment |  |  |  | -0.01 | -0.05–0.03 | 0.560 |
| Time spent on adult content |  |  |  | -0.01 | -0.04–0.02 | 0.339 |
| Time spent on social media |  |  |  | -0.04 | -0.09–0.01 | 0.142 |
| Time spent on messaging |  |  |  | 0.01 | -0.05–0.07 | 0.725 |
| Time spent on games |  |  |  | -0.01 | -0.08–0.06 | 0.718 |
| Time spent on shopping |  |  |  | 0.01 | -0.07–0.10 | 0.753 |
| Time spent on productivity |  |  |  | -0.00 | -0.07–0.06 | 0.934 |
| Time spent on the news |  |  |  | -0.01 | -0.07–0.05 | 0.842 |
| **Random Effects** | | | | | | |
| σ^2^ | 10.40 | | | 10.50 | | |
| τ_00_ | 55.83 _pid_ | | | 57.12 _pid_ | | |
| ICC | 0.84 | | | 0.84 | | |
| N | 121 _pid_ | | | 121 _pid_ | | |
| Observations | 391 | | | 391 | | |
| **** p<0.05 ** p<0.01 *** p<0.001*** | | | | | | |

**Table S42**. Results from mixed-effects models for Tier III, based on 30 days of desktop usage data.

| **Predictors** | Model 1 | | | Model 2 | | |
| --- | --- | --- | --- | --- | --- | --- |
|  | Estimate | CI | p | Estimate | CI | p |
| Intercept | 16.76*** | 13.37–20.14 | **<0.001** | 16.66*** | 13.34–19.98 | **<0.001** |
| Survey wave | -0.09 | -0.31–0.13 | 0.407 | -0.10 | -0.32–0.13 | 0.403 |
| Gender | 3.04* | 0.72–5.36 | **0.010** | 2.75* | 0.40–5.11 | **0.022** |
| Age | -1.47* | -2.91– -0.02 | **0.047** | -1.33 | -2.79–0.13 | 0.074 |
| Total time spent online | 0.00 | -0.01–0.01 | 0.659 |  |  |  |
| Daytime nighttime difference | 0.00 | -0.02–0.02 | 0.688 | -0.00 | -0.02–0.02 | 0.925 |
| Time spent on entertainment |  |  |  | 0.00 | -0.01–0.02 | 0.689 |
| Time spent on adult content |  |  |  | -0.04 | -0.10–0.02 | 0.169 |
| Time spent on social media |  |  |  | -0.01 | -0.06–0.05 | 0.831 |
| Time spent on messaging |  |  |  | 0.09 | -0.17–0.36 | 0.497 |
| Time spent on games |  |  |  | 0.01 | -0.05–0.08 | 0.640 |
| Time spent on shopping |  |  |  | 0.03 | -0.04–0.10 | 0.431 |
| Time spent on productivity |  |  |  | 0.04 | -0.02–0.09 | 0.221 |
| Time spent on the news |  |  |  | -0.13 | -0.25–0.00 | 0.056 |
| **Random Effects** | | | | | | |
| σ^2^ | 12.93 | | | 12.92 | | |
| τ_00_ | 38.23 _pid_ | | | 38.06 _pid_ | | |
| ICC | 0.75 | | | 0.75 | | |
| N | 128 _pid_ | | | 128 _pid_ | | |
| Observations | 430 | | | 430 | | |
| **** p<0.05 ** p<0.01 *** p<0.001*** | | | | | | |

**Table S43**: Results from mixed-effects models for Tier IV, based on 30 days of desktop usage data.

| **Predictors** | Model 1 | | | Model 2 | | |
| --- | --- | --- | --- | --- | --- | --- |
|  | Estimate | CI | p | Estimate | CI | p |
| Intercept | 18.46*** | 15.21–21.71 | **<0.001** | 18.72*** | 15.44–22.01 | **<0.001** |
| Survey wave | -0.33* | -0.59– -0.07 | **0.014** | -0.34* | -0.61– -0.07 | **0.013** |
| Gender | 2.28 | -0.27–4.82 | 0.080 | 2.32 | -0.24–4.89 | 0.076 |
| Age | -2.33** | -3.76– -0.90 | **0.001** | -2.31** | -3.74– -0.88 | **0.002** |
| Total time spent online | 0.01 | -0.01–0.02 | 0.294 |  |  |  |
| Daytime nighttime difference | 0.02 | -0.01–0.04 | 0.180 | 0.02 | -0.01–0.05 | 0.202 |
| Time spent on entertainment |  |  |  | -0.03 | -0.08–0.02 | 0.199 |
| Time spent on adult content |  |  |  | -0.08 | -0.23–0.07 | 0.299 |
| Time spent on social media |  |  |  | 0.02 | -0.03–0.06 | 0.443 |
| Time spent on messaging |  |  |  | 0.09 | -0.33–0.52 | 0.659 |
| Time spent on games |  |  |  | -0.05 | -0.20–0.09 | 0.471 |
| Time spent on shopping |  |  |  | 0.08* | 0.02–0.15 | **0.016** |
| Time spent on productivity |  |  |  | -0.01 | -0.08–0.06 | 0.707 |
| Time spent on news |  |  |  | 0.02 | -0.06–0.10 | 0.643 |
| **Random Effects** | | | | | | |
| σ^2^ | 11.58 | | | 11.68 | | |
| τ_00_ | 29.08 _pid_ | | | 28.14 _pid_ | | |
| ICC | 0.72 | | | 0.71 | | |
| N | 87 _pid_ | | | 87 _pid_ | | |
| Observations | 286 | | | 286 | | |
| **** p<0.05 ** p<0.01 *** p<0.001*** | | | | | | |

**Table S444.** Results from mixed-effects models for Tier V, based on 30 days of desktop usage data.

| **Predictors** | Model 1 | | | Model 2 | | |
| --- | --- | --- | --- | --- | --- | --- |
|  | Estimate | CI | p | Estimate | CI | p |
| Intercept | 19.30*** | 16.48–22.12 | **<0.001** | 18.92*** | 16.08–21.75 | **<0.001** |
| Survey wave | -0.27** | -0.48– -0.07 | **0.010** | -0.24* | -0.44– -0.03 | **0.025** |
| Gender | 0.57 | -1.64–2.78 | 0.616 | 0.37 | -1.87–2.61 | 0.746 |
| Age | -2.03** | -3.38– -0.69 | **0.003** | -1.86** | -3.23– -0.48 | **0.008** |
| Total time spent online | -0.01* | -0.03– -0.00 | **0.025** |  |  |  |
| Daytime nighttime difference | -0.00 | -0.02–0.01 | 0.708 | 0.00 | -0.02–0.02 | 0.982 |
| Time spent on entertainment |  |  |  | -0.02 | -0.04–0.01 | 0.157 |
| Time spent on adult content |  |  |  | -0.05 | -0.15–0.06 | 0.389 |
| Time spent on social media |  |  |  | 0.06* | 0.01–0.12 | **0.026** |
| Time spent on messaging |  |  |  | 0.03 | -0.06–0.12 | 0.523 |
| Time spent on games |  |  |  | -0.07 | -0.16–0.03 | 0.172 |
| Time spent on shopping |  |  |  | -0.03 | -0.09–0.04 | 0.402 |
| Time spent on productivity |  |  |  | 0.01 | -0.03–0.06 | 0.577 |
| Time spent on news |  |  |  | -0.16*** | -0.25– -0.07 | **<0.001** |
| **Random Effects** | | | | | | |
| σ^2^ | 9.43 | | | 9.06 | | |
| τ_00_ | 34.62 _pid_ | | | 35.29 _pid_ | | |
| ICC | 0.79 | | | 0.80 | | |
| N | 131 _pid_ | | | 131 _pid_ | | |
| Observations | 410 | | | 410 | | |
| **** p<0.05 ** p<0.01 *** p<0.001*** | | | | | | |

**Table S45**. Results from mixed-effects models for Tier I income group, based on 2 days of mobile usage data.

| **Predictors** | Model 1 | | | Model 2 | | |
| --- | --- | --- | --- | --- | --- | --- |
|  | Estimate | CI | p | Estimate | CI | p |
| Intercept | 19.82*** | 14.17–25.46 | **<0.001** | 19.49*** | 13.75–25.23 | **<0.001** |
| Survey wave | 0.04 | -0.28–0.36 | 0.808 | 0.05 | -0.29–0.38 | 0.771 |
| Gender | -0.05 | -4.58–4.47 | 0.982 | 0.10 | -4.41–4.62 | 0.964 |
| Age | -0.56 | -2.88–1.75 | 0.633 | -0.57 | -2.87–1.74 | 0.631 |
| Total time spent online | 0.01 | -0.09–0.12 | 0.796 |  |  |  |
| Daytime nighttime difference | 0.07 | -0.14–0.28 | 0.497 | 0.05 | -0.17–0.27 | 0.647 |
| Time spent on entertainment |  |  |  | 0.00 | -0.35–0.35 | 0.992 |
| Time spent on social media |  |  |  | 0.04 | -0.41–0.50 | 0.855 |
| Time spent on messaging |  |  |  | -0.15 | -0.77–0.46 | 0.626 |
| Time spent on games |  |  |  | 0.02 | -0.18–0.22 | 0.846 |
| Time spent on shopping |  |  |  | 0.02 | -0.97–1.01 | 0.967 |
| Time spent on productivity |  |  |  | 0.27 | -0.70–1.25 | 0.584 |
| Time spent on news |  |  |  | 1.88 | -2.78–6.53 | 0.430 |
| **Random Effects** | | | | | | |
| σ^2^ | 10.45 | | | 10.91 | | |
| τ_00_ | 63.35 _pid_ | | | 62.20 _pid_ | | |
| ICC | 0.86 | | | 0.85 | | |
| N | 52 _pid_ | | | 52 _pid_ | | |
| Observations | 183 | | | 183 | | |
| **** p<0.05 ** p<0.01 *** p<0.001*** | | | | | | |

**Table S46**. Results from mixed-effects models for the Tier II income group, based on 2 days of mobile usage data.

| **Predictors** | Model 1 | | | Model 2 | | |
| --- | --- | --- | --- | --- | --- | --- |
|  | Estimate | CI | p | Estimate | CI | p |
| Intercept | 18.43*** | 15.39–21.46 | **<0.001** | 18.71*** | 15.63–21.78 | **<0.001** |
| Survey wave | -0.01 | -0.25–0.23 | 0.920 | 0.01 | -0.24–0.25 | 0.947 |
| Gender | 0.71 | -1.61–3.03 | 0.548 | 0.81 | -1.52–3.14 | 0.495 |
| Age | -1.09 | -2.38–0.20 | 0.097 | -1.26 | -2.57–0.05 | 0.059 |
| Total time spent online | 0.09 | -0.02–0.20 | 0.122 |  |  |  |
| Daytime nighttime difference | -0.02 | -0.19–0.16 | 0.858 | -0.03 | -0.21–0.15 | 0.723 |
| Time spent on entertainment |  |  |  | 0.04 | -0.33–0.40 | 0.849 |
| Time spent on social media |  |  |  | 0.00 | -0.23–0.23 | 0.998 |
| Time spent on messaging |  |  |  | 0.18 | -0.16–0.51 | 0.301 |
| Time spent on games |  |  |  | 0.21 | -0.02–0.43 | 0.072 |
| Time spent on shopping |  |  |  | -0.21 | -1.13–0.71 | 0.655 |
| Time spent on productivity |  |  |  | -0.11 | -0.99–0.77 | 0.805 |
| Time spent on the news |  |  |  | 1.58 | -1.25–4.41 | 0.273 |
| **Random Effects** | | | | | | |
| σ^2^ | 14.35 | | | 14.53 | | |
| τ_00_ | 39.97 _pid_ | | | 39.63 _pid_ | | |
| ICC | 0.74 | | | 0.73 | | |
| N | 136 _pid_ | | | 136 _pid_ | | |
| Observations | 426 | | | 426 | | |
| **** p<0.05 ** p<0.01 *** p<0.001*** | | | | | | |

**Table S47**. Results from mixed-effects models for the Tier III income group, based on 2 days of mobile usage data.

| **Predictors** | Model 1 | | | Model 2 | | |
| --- | --- | --- | --- | --- | --- | --- |
|  | Estimate | CI | p | Estimate | CI | p |
| Intercept | 16.10*** | 13.26–18.94 | **<0.001** | 15.75*** | 12.91–18.59 | **<0.001** |
| Survey wave | -0.07 | -0.27–0.12 | 0.453 | -0.06 | -0.25–0.14 | 0.570 |
| Gender | 4.49*** | 2.38–6.61 | **<0.001** | 4.33*** | 2.21–6.45 | **<0.001** |
| Age | -1.47* | -2.76– -0.18 | **0.025** | -1.29 | -2.58–0.00 | 0.051 |
| Total time spent online | 0.02 | -0.08–0.12 | 0.704 |  |  |  |
| Daytime nighttime difference | 0.08 | -0.07–0.22 | 0.311 | 0.05 | -0.10–0.20 | 0.496 |
| Time spent on entertainment |  |  |  | -0.00 | -0.27–0.26 | 0.990 |
| Time spent on social media |  |  |  | -0.04 | -0.36–0.29 | 0.829 |
| Time spent on messaging |  |  |  | 0.48** | 0.14–0.82 | **0.006** |
| Time spent on games |  |  |  | 0.09 | -0.09–0.27 | 0.343 |
| Time spent on shopping |  |  |  | -0.10 | -0.66–0.46 | 0.729 |
| Time spent on productivity |  |  |  | -0.22 | -0.57–0.12 | 0.207 |
| Time spent on the news |  |  |  | -0.75 | -1.58–0.09 | 0.081 |
| **Random Effects** | | | | | | |
| σ^2^ | 13.10 | | | 12.89 | | |
| τ_00_ | 40.40 _pid_ | | | 40.11 _pid_ | | |
| ICC | 0.76 | | | 0.76 | | |
| N | 159 _pid_ | | | 159 _pid_ | | |
| Observations | 548 | | | 548 | | |
| **** p<0.05 ** p<0.01 *** p<0.001*** | | | | | | |

**Table S48**. Results from mixed-effects models for the Tier IV income group, based on 2 days of mobile usage data.

| **Predictors** | Model 1 | | | Model 2 | | |
| --- | --- | --- | --- | --- | --- | --- |
|  | Estimate | CI | p | Estimate | CI | p |
| Intercept | 21.48*** | 18.82–24.14 | **<0.001** | 21.47*** | 18.78–24.15 | **<0.001** |
| Survey wave | -0.28** | -0.47– -0.09 | **0.004** | -0.28** | -0.48– -0.08 | **0.005** |
| Gender | 1.18 | -0.90–3.27 | 0.266 | 1.17 | -0.95–3.28 | 0.280 |
| Age | -2.70*** | -3.93– -1.47 | **<0.001** | -2.71*** | -3.95– -1.47 | **<0.001** |
| Total time spent online | -0.07 | -0.18–0.03 | 0.160 |  |  |  |
| Daytime nighttime difference | 0.18* | 0.01–0.34 | **0.038** | 0.18* | 0.00–0.36 | **0.044** |
| Time spent on entertainment |  |  |  | -0.14 | -0.49–0.22 | 0.446 |
| Time spent on social media |  |  |  | -0.04 | -0.31–0.24 | 0.800 |
| Time spent on messaging |  |  |  | -0.11 | -0.49–0.27 | 0.573 |
| Time spent on games |  |  |  | -0.08 | -0.30–0.15 | 0.494 |
| Time spent on shopping |  |  |  | -0.01 | -0.65–0.63 | 0.974 |
| Time spent on productivity |  |  |  | -0.14 | -0.92–0.63 | 0.716 |
| Time spent on the news |  |  |  | -0.19 | -1.54–1.16 | 0.784 |
| **Random Effects** | | | | | | |
| σ^2^ | 9.64 | | | 9.82 | | |
| τ_00_ | 33.58 _pid_ | | | 33.52 _pid_ | | |
| ICC | 0.78 | | | 0.77 | | |
| N | 133 _pid_ | | | 133 _pid_ | | |
| Observations | 452 | | | 452 | | |
| **** p<0.05 ** p<0.01 *** p<0.001*** | | | | | | |

**Table S49**. Results from mixed-effects models for Tier V income group, based on 2 days of mobile usage data.

| **Predictors** | Model 1 | | | Model 2 | | |
| --- | --- | --- | --- | --- | --- | --- |
|  | Estimate | CI | p | Estimate | CI | p |
| Intercept | 16.34*** | 13.73–18.96 | **<0.001** | 15.98*** | 13.35–18.60 | **<0.001** |
| Survey wave | -0.12 | -0.31–0.07 | 0.203 | -0.13 | -0.32–0.06 | 0.180 |
| Gender | 1.49 | -0.53–3.51 | 0.149 | 1.37 | -0.65–3.39 | 0.184 |
| Age | -1.80** | -3.12– -0.48 | **0.008** | -1.59* | -2.92– -0.26 | **0.019** |
| Total time spent online | -0.03 | -0.13–0.07 | 0.553 |  |  |  |
| Daytime nighttime difference | -0.02 | -0.15–0.12 | 0.819 | -0.02 | -0.16–0.12 | 0.792 |
| Time spent on entertainment |  |  |  | 0.21 | -0.06–0.48 | 0.127 |
| Time spent on social media |  |  |  | 0.13 | -0.18–0.43 | 0.417 |
| Time spent on messaging |  |  |  | -0.17 | -0.52–0.18 | 0.344 |
| Time spent on games |  |  |  | -0.19* | -0.37– -0.00 | **0.047** |
| Time spent on shopping |  |  |  | 0.11 | -0.58–0.80 | 0.757 |
| Time spent on productivity |  |  |  | 0.33 | -0.09–0.75 | 0.120 |
| Time spent on the news |  |  |  | -0.74 | -1.60–0.12 | 0.090 |
| **Random Effects** | | | | | | |
| σ^2^ | 11.54 | | | 11.42 | | |
| τ_00_ | 34.05 _pid_ | | | 33.83 _pid_ | | |
| ICC | 0.75 | | | 0.75 | | |
| N | 160 _pid_ | | | 160 _pid_ | | |
| Observations | 532 | | | 532 | | |
| **** p<0.05 ** p<0.01 *** p<0.001*** | | | | | | |

**Table S50**. Results from mixed-effects models for Tier I, based on 2 days of desktop usage data.

| **Predictors** | Model 1 | | | Model 2 | | |
| --- | --- | --- | --- | --- | --- | --- |
|  | Estimate | CI | p | Estimate | CI | p |
| Intercept | 22.08*** | 16.61–27.54 | **<0.001** | 21.92*** | 16.32–27.52 | **<0.001** |
| Survey wave | -0.09 | -0.47–0.28 | 0.622 | -0.07 | -0.46–0.31 | 0.711 |
| Gender | -0.64 | -4.53–3.25 | 0.747 | -1.12 | -5.14–2.91 | 0.586 |
| Age | -1.74 | -4.17–0.70 | 0.162 | -1.20 | -3.77–1.38 | 0.362 |
| Total time spent online | -0.02 | -0.15–0.12 | 0.811 |  |  |  |
| Daytime nighttime difference | 0.07 | -0.12–0.26 | 0.448 | 0.05 | -0.16–0.25 | 0.651 |
| Time spent on entertainment |  |  |  | 0.09 | -0.13–0.32 | 0.424 |
| Time spent on adult content |  |  |  | -0.02 | -0.34–0.30 | 0.910 |
| Time spent on social media |  |  |  | -0.15 | -0.51–0.21 | 0.419 |
| Time spent on messaging |  |  |  | -2.86 | -18.44–12.72 | 0.719 |
| Time spent on games |  |  |  | -0.37 | -0.94–0.21 | 0.214 |
| Time spent on shopping |  |  |  | -0.15 | -0.95–0.64 | 0.706 |
| Time spent on productivity |  |  |  | -0.16 | -0.91–0.58 | 0.674 |
| Time spent on news |  |  |  | -0.87 | -3.76–2.01 | 0.552 |
| **Random Effects** | | | | | | |
| σ^2^ | 12.27 | | | 12.36 | | |
| τ_00_ | 47.27 _pid_ | | | 49.06 _pid_ | | |
| ICC | 0.79 | | | 0.80 | | |
| N | 56 _pid_ | | | 56 _pid_ | | |
| Observations | 153 | | | 153 | | |
| **** p<0.05 ** p<0.01 *** p<0.001*** | | | | | | |

**Table S51**. Results from mixed-effects models for Tier II, based on 2 days of desktop usage data.

| **Predictors** | Model 1 | | | Model 2 | | |
| --- | --- | --- | --- | --- | --- | --- |
|  | Estimate | CI | p | Estimate | CI | p |
| Intercept | 18.78*** | 14.77–22.79 | **<0.001** | 18.42*** | 14.37–22.46 | **<0.001** |
| Survey wave | 0.14 | -0.12–0.40 | 0.304 | 0.17 | -0.10–0.44 | 0.225 |
| Gender | 1.73 | -1.16–4.61 | 0.241 | 1.79 | -1.12–4.69 | 0.228 |
| Age | -1.33 | -2.91–0.24 | 0.098 | -1.27 | -2.87–0.33 | 0.119 |
| Total time spent online | -0.14 | -0.32–0.04 | 0.123 |  |  |  |
| Daytime nighttime difference | 0.15 | -0.07–0.36 | 0.189 | 0.13 | -0.10–0.37 | 0.258 |
| Time spent on entertainment |  |  |  | 0.08 | -0.31–0.47 | 0.684 |
| Time spent on adult content |  |  |  | -0.18 | -0.55–0.19 | 0.347 |
| Time spent on social media |  |  |  | 0.18 | -0.41–0.78 | 0.552 |
| Time spent on messaging |  |  |  | -0.60 | -1.52–0.33 | 0.208 |
| Time spent on games |  |  |  | -0.19 | -0.91–0.52 | 0.593 |
| Time spent on shopping |  |  |  | -0.41 | -1.23–0.40 | 0.321 |
| Time spent on productivity |  |  |  | -0.34 | -1.01–0.34 | 0.329 |
| Time spent on the news |  |  |  | -0.19 | -0.77–0.38 | 0.510 |
| **Random Effects** | | | | | | |
| σ^2^ | 10.40 | | | 10.66 | | |
| τ_00_ | 52.64 _pid_ | | | 52.17 _pid_ | | |
| ICC | 0.83 | | | 0.83 | | |
| N | 109 _pid_ | | | 109 _pid_ | | |
| Observations | 287 | | | 287 | | |
| **** p<0.05 ** p<0.01 *** p<0.001*** | | | | | | |

**Table S52**. Results from mixed-effects models for Tier III, based on 2 days of desktop usage data.

| **Predictors** | Model 1 | | | Model 2 | | |
| --- | --- | --- | --- | --- | --- | --- |
|  | Estimate | CI | p | Estimate | CI | p |
| Intercept | 16.80*** | 13.39–20.22 | **<0.001** | 17.16*** | 13.75–20.57 | **<0.001** |
| Survey wave | -0.04 | -0.30–0.21 | 0.741 | -0.04 | -0.30–0.23 | 0.775 |
| Gender | 2.38 | -0.00–4.76 | 0.050 | 2.19 | -0.23–4.61 | 0.076 |
| Age | -1.47* | -2.94– -0.00 | **0.050** | -1.48 | -2.96–0.00 | 0.051 |
| Total time spent online | 0.05 | -0.08–0.17 | 0.460 |  |  |  |
| Daytime nighttime difference | -0.08 | -0.28–0.12 | 0.438 | -0.06 | -0.28–0.16 | 0.612 |
| Time spent on entertainment |  |  |  | 0.08 | -0.06–0.23 | 0.260 |
| Time spent on adult content |  |  |  | -0.24 | -1.08–0.60 | 0.572 |
| Time spent on social media |  |  |  | -0.03 | -0.64–0.59 | 0.932 |
| Time spent on messaging |  |  |  | 0.44 | -10.53–11.40 | 0.938 |
| Time spent on games |  |  |  | -0.14 | -0.92–0.63 | 0.721 |
| Time spent on shopping |  |  |  | 0.09 | -0.59–0.76 | 0.799 |
| Time spent on productivity |  |  |  | 0.17 | -0.46–0.80 | 0.590 |
| Time spent on the news |  |  |  | -1.13 | -2.33–0.06 | 0.063 |
| **Random Effects** | | | | | | |
| σ^2^ | 10.52 | | | 10.67 | | |
| τ_00_ | 41.15 _pid_ | | | 40.89 _pid_ | | |
| ICC | 0.80 | | | 0.79 | | |
| N | 130 _pid_ | | | 130 _pid_ | | |
| Observations | 298 | | | 298 | | |
| **** p<0.05 ** p<0.01 *** p<0.001*** | | | | | | |

**Table S53**. Results from mixed-effects models for Tier IV, based on 2 days of desktop usage data.

| **Predictors** | Model 1 | | | Model 2 | | |
| --- | --- | --- | --- | --- | --- | --- |
|  | Estimate | CI | p | Estimate | CI | p |
| Intercept | 20.32*** | 17.03–23.62 | **<0.001** | 20.57*** | 17.21–23.92 | **<0.001** |
| Survey wave | -0.55*** | -0.85– -0.24 | **<0.001** | -0.46** | -0.77– -0.14 | **0.004** |
| Gender | 2.75* | 0.41–5.10 | **0.022** | 2.64* | 0.24–5.04 | **0.031** |
| Age | -3.41*** | -4.83– -2.00 | **<0.001** | -3.44*** | -4.88– -2.00 | **<0.001** |
| Total time spent online | 0.23* | 0.04–0.42 | **0.019** |  |  |  |
| Daytime nighttime difference | 0.23 | -0.00–0.46 | 0.050 | 0.20 | -0.09–0.48 | 0.176 |
| Time spent on entertainment |  |  |  | 0.12 | -0.32–0.56 | 0.603 |
| Time spent on adult content |  |  |  | -0.35 | -1.28–0.57 | 0.455 |
| Time spent on social media |  |  |  | 0.60 | -0.13–1.33 | 0.105 |
| Time spent on messaging |  |  |  | -3.03 | -12.37–6.32 | 0.525 |
| Time spent on games |  |  |  | 0.62 | -1.25–2.49 | 0.515 |
| Time spent on shopping |  |  |  | 0.44 | -0.31–1.20 | 0.248 |
| Time spent on productivity |  |  |  | -0.06 | -0.65–0.53 | 0.848 |
| Time spent on the news |  |  |  | -0.19 | -1.70–1.31 | 0.800 |
| **Random Effects** | | | | | | |
| σ^2^ | 10.21 | | | 10.67 | | |
| τ_00_ | 24.52 _pid_ | | | 24.74 _pid_ | | |
| ICC | 0.71 | | | 0.70 | | |
| N | 88 _pid_ | | | 88 _pid_ | | |
| Observations | 206 | | | 206 | | |
| **** p<0.05 ** p<0.01 *** p<0.001*** | | | | | | |

**Table S54**. Results from mixed-effects models for Tier V, based on 2 days of desktop usage data.

| **Predictors** | Model 1 | | | Model 2 | | |
| --- | --- | --- | --- | --- | --- | --- |
|  | Estimate | CI | p | Estimate | CI | p |
| Intercept | 18.71*** | 15.45–21.97 | **<0.001** | 18.65*** | 15.40–21.91 | **<0.001** |
| Survey wave | -0.24 | -0.52–0.04 | 0.090 | -0.26 | -0.54–0.02 | 0.072 |
| Gender | 0.14 | -2.33–2.62 | 0.909 | 0.20 | -2.29–2.69 | 0.876 |
| Age | -2.01** | -3.53– -0.49 | **0.009** | -2.03** | -3.57– -0.50 | **0.009** |
| Total time spent online | -0.08 | -0.23–0.07 | 0.295 |  |  |  |
| Daytime nighttime difference | -0.05 | -0.22–0.12 | 0.560 | -0.03 | -0.21–0.15 | 0.741 |
| Time spent on entertainment |  |  |  | -0.12 | -0.41–0.16 | 0.394 |
| Time spent on adult content |  |  |  | -0.49 | -1.31–0.32 | 0.235 |
| Time spent on social media |  |  |  | 0.25 | -0.40–0.91 | 0.448 |
| Time spent on messaging |  |  |  | -0.44 | -1.47–0.60 | 0.409 |
| Time spent on games |  |  |  | -0.37 | -1.25–0.51 | 0.410 |
| Time spent on shopping |  |  |  | -0.45 | -1.06–0.16 | 0.149 |
| Time spent on productivity |  |  |  | 0.64* | 0.07–1.21 | **0.027** |
| Time spent on the news |  |  |  | -1.04** | -1.76– -0.32 | **0.005** |
| **Random Effects** | | | | | | |
| σ^2^ | 10.19 | | | 9.48 | | |
| τ_00_ | 36.73 _pid_ | | | 37.33 _pid_ | | |
| ICC | 0.78 | | | 0.80 | | |
| N | 118 _pid_ | | | 118 _pid_ | | |
| Observations | 263 | | | 263 | | |
| **** p<0.05 ** p<0.01 *** p<0.001*** | | | | | | |
